# The Combined Loss of Triose Phosphate and Xylulose 5-Phosphate/Phosphate Translocators Leads to Severe Growth Retardation and Impaired Photosynthesis in *Arabidopsis thaliana tpt/xpt* Double Mutants

**DOI:** 10.3389/fpls.2018.01331

**Published:** 2018-10-02

**Authors:** Elke J. A. Hilgers, Mark Aurel Schöttler, Tabea Mettler-Altmann, Stephan Krueger, Peter Dörmann, Michael Eicks, Ulf-Ingo Flügge, Rainer E. Häusler

**Affiliations:** ^1^Department of Biology, Cologne Biocenter, Botanical Institute II and Cluster of Excellence on Plant Sciences, University of Cologne, Cologne, Germany; ^2^Max-Planck-Institut für Molekulare Pflanzenphysiologie, Potsdam, Germany; ^3^Plant Biochemistry, Heinrich Heine University Düsseldorf, Düsseldorf, Germany; ^4^Molecular Biotechnology and Biochemistry, Universität Bonn, Bonn, Germany; ^5^Berufskolleg Kartäuserwall, Cologne, Germany

**Keywords:** transport, chloroplasts, photosynthesis, pentose phosphates, signaling

## Abstract

The xylulose 5-phosphate/phosphate translocator (XPT) represents the fourth functional member of the phosphate translocator (PT) family residing in the plastid inner envelope membrane. In contrast to the other three members, little is known on the physiological role of the XPT. Based on its major transport substrates (i.e., pentose phosphates) the XPT has been proposed to act as a link between the plastidial and extraplastidial branches of the oxidative pentose phosphate pathway (OPPP). As the XPT is also capable of transporting triose phosphates, it might as well support the triose phosphate PT (TPT) in exporting photoassimilates from the chloroplast in the light (‘day path of carbon’) and hence in supplying the whole plant with carbohydrates. Two independent knockout mutant alleles of the XPT (*xpt-1* and *xpt-2*) lacked any specific phenotype, suggesting that the XPT function is redundant. However, double mutants generated from crossings of *xpt-1* to different mutant alleles of the TPT (*tpt-1* and *tpt-2*) were severely retarded in size, exhibited a high chlorophyll fluorescence phenotype, and impaired photosynthetic electron transport rates. In the double mutant the export of triose phosphates from the chloroplasts is completely blocked. Hence, precursors for sucrose biosynthesis derive entirely from starch turnover (‘night path of carbon’), which was accompanied by a marked accumulation of maltose as a starch breakdown product. Moreover, pentose phosphates produced by the extraplastidial branch of the OPPP also accumulated in the double mutants. Thus, an active XPT indeed retrieves excessive pentose phosphates from the extra-plastidial space and makes them available to the plastids. Further metabolic profiling revealed that phosphorylated intermediates remained largely unaffected, whereas fumarate and glycine contents were diminished in the double mutants. The assessment of C/N-ratios suggested co-limitations of C- and N-metabolism as possible cause for growth retardation of the double mutants. Feeding of sucrose partially rescued the growth and photosynthesis phenotypes of the double mutants. Immunoblots of thylakoid proteins, spectroscopic determinations of photosynthesis complexes, and chlorophyll *a* fluorescence emission spectra at 77 Kelvin could only partially explain constrains in photosynthesis observed in the double mutants. The data are discussed together with aspects of the OPPP and central carbon metabolism.

## Introduction

Phosphate translocators (PT) of the plastidial inner envelope membrane belong to the super-family of nucleotide sugar transporters (Knappe et al., 2003; Orellana et al., 2016). They play profound roles in the exchange of phosphorylated metabolic intermediates between the plastid stroma and the surrounding cytosol (Flügge, 1999). The most prominent member of the PT family is the triose phosphate/PT (TPT, Fliege et al., 1978), which represents the major exporter of photoassimilates in the light, i.e. the ‘day path of carbon’. Up to now three more members of the PT family have been characterized based on their substrate specificities (e.g., Fischer, 2011; Flügge et al., 2011). The phosphoenolpyruvate (PEP)/PT (PPT; Fischer et al., 1997) is encoded by two genes in *A. thaliana*. The proposed role of the PPT is the provision of PEP to chloroplasts and other plastid types that lack a complete plastidial glycolysis (Streatfield et al., 1999; Flügge et al., 2011). PEP and erythrose 4-P (E-4-P) are the initial substrates for the plastid-localized shikimate pathway, from which aromatic amino acids and a plethora of downstream products derive (Tzin and Galili, 2010). The glucose 6-phosphate (Glc6P)/PT (GPT; Kammerer et al., 1998) is also encoded by two genes in *A. thaliana*. Both *GPT* genes are mainly expressed in non-green tissues and the corresponding GPT transporters provide plastids with carbon skeletons in form of Glc6P for starch biosynthesis and/or the oxidative pentose phosphate pathway (OPPP).

Here we report on the functional characterization of the fourth member of the PT family, the xylulose 5-phosphate Xu5P/PT (XPT), which was initially isolated and kinetically characterized by Eicks et al. (2002). As a single copy gene in *A. thaliana*, the *XPT* is ubiquitously expressed, but its metabolic role in green and non-green tissues is still unclear. The original proposal, i.e., that the XPT might link plastidial with extra-plastidial branches of the OPPP by exchanging pentose phosphates, has been widely accepted (e.g., Kruger and von Schaewen, 2003; Facchinelli and Weber, 2011). However, so far experimental evidence in support of this view is still missing. In non-green plastids and chloroplasts at night the OPPP consumes Glc6P to provide both reducing power in form of NADPH for anabolic pathways, like *de novo* fatty acids synthesis, and metabolic precursors, like E-4-P for the shikimate pathway or ribose 5-P for nucleotide biosynthesis (Kruger and von Schaewen, 2003). Various isoenzymes of the initial, non-reversible steps of the OPPP (i.e., Glc6P-dehydrogenase [G6PDH], 6-P-gluconolactonase, [6PGL] and 6-P-gluconate-dehydrogenase [PGD]) are also localized in the cytosol and, at least temporarily, in the peroxisomes (Reumann et al., 2004; Xiong et al., 2009; Meyer et al., 2011; Hölscher et al., 2014, Hölscher et al., 2016). There is increasing evidence for the involvement of a cytosolic thioredoxin, and hence the redox-state of the cells, in targeting OPPP enzymes either to plastids or peroxisomes (Meyer et al., 2011; Hölscher et al., 2014, Hölscher et al., 2016). The requirement for enhanced NADPH generation by the OPPP in the different compartments of the cell might be triggered by various stresses (e.g., Scharte et al., 2009, and references therein).

Transport studies revealed that the XPT is capable of transporting TPs and PEP besides of Xu5P (Eicks et al., 2002). Hence the functional role of the XPT might also be closely linked to the TPT in photosynthetic tissues, or the PPT in both green and non-green parts of the plants. In this report we will focus on possible functional connections between the XPT and TPT by using mutant and transgenic approaches.

The consequences of a loss in TPT function or diminished activity have been described extensively over the last two decades. Mutant and transgenic plants defective in the TPT can almost completely compensate for the block in the ‘day path of carbon’ by an increased mobilization of transitory starch both in the dark and light (Häusler et al., 1998; Schneider et al., 2002; Walters et al., 2004; Schmitz et al., 2012). This ‘night path of carbon’ is more complex compared to the ‘day path’. Here starch is degraded by the action of β-amylase, isoamylase and disproportionating enzyme (DPE1), yielding maltose and glucose (Glc) as end products (Weise et al., 2004; Zeeman et al., 2010). Furthermore, both sugars are exported from the chloroplasts by the maltose exporter, (MEX, Niittylä et al., 2004) and the Glc transporter (GlcT, Weber et al., 2000). Finally, in the cytosol, maltose is metabolized via DPE2 (Lu and Sharkey, 2004, 2006) a cytosolic heteroglycan (Fettke et al., 2008) and cytosolic glucan phosphorylase (in *A. thaliana* PSH2; Fettke et al., 2007) resulting in Glc and Glc1P, which enter further metabolism. The importance of starch turnover for survival in the absence of the TPT has been addressed by crossing of *tpt* mutants to starch-free mutants (e.g., with a defect in the catalytic subunit of ADPglucose pyrophosphorylase [AGPase, *adg1-1*; Lin et al., 1988]). Surprisingly the double mutants were viable, but severely retarded in size, showed impaired photosynthesis (Schneider et al., 2002; Schmitz et al., 2012), and a high chlorophyll fluorescence (HCF)-phenotype in the dark-adapted state. (Häusler et al., 2009; Schmitz et al., 2012). The HCF-phenotype was mainly based on diminished levels of core components of both photosystems, and highly abundant light harvesting complex (LHC) proteins that were not attached to their core components (Schmitz et al., 2012). It was hypothesized that malfunctions in retrograde signaling coordinating the expression of nuclear- and plastome-encoded photosynthesis genes were responsible for the observed imbalance between antennae and reaction centers in *adg1-1/tpt-2* (Häusler et al., 2014a, and references therein).

In this report the consequences of a loss of XPT function on plant performance and metabolism will be analyzed. Neither independent knockout mutant alleles of the XPT (*xpt-1* and *xpt-2*) nor transgenic plants overexpressing an artificial microRNA construct against the *XPT* showed any phenotype during vegetative development. However, double mutants impaired in both the TPT and XPT were severely retarded in growth and showed a HCF-phenotype similar, but not identical, to the *adg1-1/tpt-1[2]* double mutant. The underlying reasons for these unexpected similarities between both double mutants will be addressed and discussed.

## Materials and Methods

### Plant Material and Growth Conditions

Seeds of *A. thaliana* ecotypes Ws-2 and Col-0 were obtained from the Nottingham Arabidopsis Stock Centre (NASC). In addition, the following mutant lines defective in the gene indicated were used: *gpt2-1* (At1g61800; Niewiadomski et al., 2005), *tpt-1* and *tpt-2* (At5g46110; Schneider et al., 2002; Schmitz et al., 2012). The mutations in the *TPT*-gene are located in different backgrounds (i.e., *tpt-1* in Ws-2 and *tpt-2* in Col-0) and represent either a knockdown (*tpt-1*; Schneider et al., 2002) or a knockout (*tpt-2*; Schmitz et al., 2012) of the TPT. As an additional control, the *adg1-1/tpt-2* double mutant (Schmitz et al., 2012) was used in most experiments. It derives from crossings of the starch-free mutant *adg1-1* (Lin et al., 1988) with *tpt-2*. In cases when two alleles of a mutant are referred to in the text, tables, or figures (e.g., *tpt-1* and *tpt-2*) they are combined to e.g., *tpt-1[2]*.

If not stated otherwise, plants were germinated and grown in soil (Einheitserde, Type Minitray, Gebr. Patzer KG, Sinntal-Jossa) mixed with 30% Vermiculite for approximately two to three weeks in a growth chamber (Johnson Control) equipped with eight dimmable fluorescence tubes (Osram L18W/840) either in the long-day (16 h light/8 h dark) or short-day (8 h light/16 h dark) at day/night temperatures of 21°C/18°C. Under long-day conditions plants were grown at three different photon flux densities (PFD) at rosette leaf level, i.e., at low light (LL) 30 μmol⋅m^-2^⋅s^-1^, standard light (SL) 150 μmol⋅m^-2^⋅s^-1^, and high light (HL) 300 μmol⋅m^-2^⋅s^-1^. In the short-day plants were grown under SL-conditions. The humidity was kept at approximately 60%. For growth analyses time point zero refers to ‘days after sowing’ (DAS) excluding a 48 h period of stratification at 4°C. For selection with BASTA (phosphinothricin), the plants (i.e., *xpt-1*, *xpt-2*, and *xpt-3;* see below) were grown in a temperature controlled greenhouse. For selection with kanamycin or for feeding experiments, plants were grown on 1/2 strength Murashige-Skoog (MS) agar supplemented with modified vitamins (Duchefa M0245) in a growth cabinet (Percival, CLF Plant Climatics GmBH, Wertingen, Germany, model AR-36L3/HIL) at a light/dark cycle of 16 h/8 h, a day/night temperature of 22°C/18°C and at relative humidity of 40%. Each of the three levels of the growth cabinet was equipped with 14 fluorescence tubes (Osram L18W/840), which could be dimmed to the desired PFD. Feeding experiments with exogenous carbohydrates, or amino acids were performed after surface-sterilized seeds were germinated and grown on sterile 1/2 MS agar with or without 50 mM Suc and/or 2 mM Gln for up to three weeks under HL-conditions in the long-day.

### Determination of the Area Growth of Leaf Rosettes

The area growth of leaf rosettes was determined non-destructively from pictures taken beween days 6 and 21 after sowing of plants grown in soil or on 1/2 MS agar plates. The areas were calculated using ImageJ^1^ by comparing to a known area. The areas were not corrected for overlapping leaves (occuring at later stages of development). Shaded leaves contribute less to photosynthesis as compared to leaves exposed to light. In some cases rosettes were harvested at the same time intervals and the fresh or dry weights determined destructively.

### Isolation of the *xpt-1, xpt-2* and *xpt-3* Mutant Alleles and Generation of Double and Triple, Mutants

Three T-DNA insertions in the *XPT* gene (At5g17630) were identified in this study. The *xpt-1* mutant allele (Ws-2 background) was isolated from the T-DNA insertion pool P39F3 obtained from the Arabidopsis Knockout Facility (Madison, Wisconsin, United States). The pool confers BASTA resistance as a selection marker. The *xpt-2* and *xpt-3* mutant alleles were obtained from NASC (N817418 [Sail_378_C01, Col-3 background] and N836198 [Sail_819_D09, Col-0 background]). As a visual marker the *xpt-2* line contains a secondary mutation in a pectin esterase *quartet1* (*qrt1-2*) leading to mature pollen tetrades (Francis et al., 2006). The *xpt-1*, *xpt-2*, *xpt-3* mutant alleles were screened by standard PCR on genomic DNA using *XPT-1* (f; r), *XPT-2* (f; r), or *XPT-3* (f; r) primers in combination with T-DNA border primers (**Supplementary Table 1**).

**Table 1:** Generative growth characteristics of wild-type, mutant, and amiRNA plants defective in the TPT and/or XPT.

Plant line	Inflorencence height	Number of siliques	Seed weight per plant	Seed weight per 100 seeds	Seed number per plant	Seed area	
					
	(cm)		(g)	(mg)		(mm^2^)	*n*
Ws-2	43.9 ± 1.3	322.8 ± 15.6	0.194 ± 0.016	1.63 ± 0.02	11822 ± 986	0.0954 ± 0.0003	894
*xpt-1*	51.0 ± 0.4	408.4 ± 31.0	0.165 ± 0.014	2.82 ± 0.09	5873 ± 556	0.1416 ± 0.0006	740
Col-0	42.4 ± 0.6	223.8 ± 16.4	0.131 ± 0.018	1.63 ± 0.05	7959 ± 1035	0.0959 ± 0.0004	1082
*tpt-2*	42.7 ± 1.2	206.2 ± 9.3	0.117 ± 0.004	1.74 ± 0.02	6715 ± 161	0.1007 ± 0.0003	1035
*tpt-2/xpt-1*	32.1 ± 1.2*	94.8 ± 7.2*	0.034 ± 0.006	1.58 ± 0.03	2140 ± 382	0.0936 ± 0.0004	952
amiRNA:*XPT tpt-2* #3	41.8 ± 0.6	194.6 ± 8.3	0.101 ± 0.007	1.74 ± 0.02	5814 ± 414	0.0985 ± 0.0003	1037
amiRNA:*XPT tpt-2* #4	40.2 ± 1.4	182.2 ± 9.6	0.060 ± 0.009	1.79 ± 0.15	3356 ± 446	0.1035 ± 0.0006	773


*Inflorescence heights and number of siliques were determined for plants grown in soil in a temperature controlled growth cabinet under SL-conditions (PFD = 150 μmol⋅m^-*2*^⋅s^-*1*^) in the long- for 56 days after sowing. Seed weights and numbers as well as seed areas were measured after ripening and drying of the plants. The data represent the mean ± SE of *n* = 5 or *n* = 4^∗^ determinations. The numbers of replicates for seed area determinations are given in the table. A statistical analysis of growth parameters is contained in **Doc S2, Table 1**.*

The *xpt-1* mutant line was crossed to *tpt-1*, *tpt-2*, and *gpt2-1.* Furthermore *gpt2-1/tpt-2/xpt-1* triple mutants were obtained by crossing the homozygous double mutant *tpt-2/xpt-1* to *gpt2-1/xpt-1*. For crossings immature flowers of homozygous single mutants were emasculated and manually cross-pollinated. For double mutants the mutant mentioned first has been the female parent. The progenies of the crosses were screened by PCR on genomic DNA (**Supplementary Table 1**).

### Generation of amiRNA:*XPT* in Wild-Type and *tpt-2* Lines

Ecotype-independent reduction of *XPT* expression was achieved by transformation with artificial microRNA (amiRNA). The appropriate amiRNA sequence was obtained from the ‘Web microRNA designer’ at Weigelworld^2^. The amiRNA constructs for the *XPT* gene were generated according to Schwab et al. (2006) under control of the CaMV 35S promoter. For the *XPT* two vector specific primers (pRS300A und pRS300B) as well as four primers containing elements of the vector and the amiRNA-sequence (*XPT*, miR-s I-IV) were used in a Gateway L/R system (Nakagawa et al., 2007; **Supplementary Table 1**). The resulting destination vector was transformed via *Agrobacterium tumefaciens* mediated gene transfer into wild-type and mutant *A. thaliana* plants by the ‘floral dip’ method (Clough and Bent, 1998). The presence of the amiRNA:*XPT* construct in the plants was tested by PCR on genomic DNA using CaMV 35S and pRS300B (r) primers (see **Supplementary Table 1**).

### DNA and RNA Extraction and PCR Methods

Genomic DNA was extracted according to Edwards et al. (1991). RNA was extracted in hot phenol as described in Collart and Oliviero (2001). After treatment with RNase-free DNase (Ambion), oligo(dT)-primed cDNA of total RNA was synthesized using the Bioscript reverse transcriptase (Bioline). PCR with cDNA or gDNA was performed using *Taq* polymerase (Thermo Fisher Scientific) according to Mullis et al. (1986) at between 26 and 32 cycles. Amplified DNA was separated on 1.0% agarose gels in the presence of Tris-acetate buffer and 5 μg⋅ml^-1^ ethidium bromide.

### Protein Separation and Immunoblot Analyses

Samples for protein isolation and subsequent immunoblot analyses were taken directly in the Percival growth cabinet or the phytochamber under the respective conditions after 8 h of illumination.

Total leaf protein was extracted and separated by discontinuous sodium dodecyl sulfate-polyacrylamide gel electrophoresis (SDS–PAGE) according to Laemmli (1970) on 15% separation gels. For immunological detection, proteins were transferred from the SDS-gels to polyvinylidene fluoride (PVDF)-membranes (BioRad, Munich) by electroblotting according to Jungblut and Seifert (1990) using a ‘semi-dry’ blotting apparatus (Carboglass; Schleicher and Schüll).

The membranes were incubated for 2 h in a casein containing blocking solution and probed overnight at 4°C with primary antibodies against photosynthesis-associated proteins supplied by Agrisera (Vännäs, Sweden). Following incubation with the secondary antibody, i.e. horseradish peroxidase conjugate (Sigma, St. Louis, MO, United States), the proteins were detected by chemoluminescence following the application of SuperSignal West substrate (Thermo Fisher Scientific) in an ImageQuant LAS-4000 Luminescent Analyser (GE Healthcare).

### Carbohydrate and Amino Acid Determinations

Carbohydrates: For the extraction of starch and soluble sugars lyophilized leaf material was treated as described in (Lin et al., 1988) and determined with a coupled enzymatic assay (Stitt et al., 1989) in a Spectrafluor Plus plate reader (TECAN, Austria) in the absorbance mode.

Amino acids: Lyophilized tissue (2–8 mg) was homogenized by a TissueLyser (Quiagen) at 23 Hz for 30 s and subsequently extracted with 300 μl of 80% EtOH at 4°C for 20 min on an orbital shaker. The mixture was centrifuged at 21000 × *g* for 5 min at 4°C and the supernatants were transferred to fresh 2 ml test tubes, while the debris was extracted with 200 μl 96% EtOH and again incubated at 4°C for 20 min on an orbital shaker. Following centrifugation at 21000 × *g* for 5 min at 4°C the second supernatants were unified with the first ones in the 2 ml test tubes. The analysis of free amino acid contents was carried out as outlined in Krueger et al. (2017) by reversed phase HPLC using a HyperClone^TM^ 5 μm ODS (C18) column (Phenomenex). The samples were precolumn-derivatized with o-phthalaldehyde according Lindroth and Mopper (1979).

### Fatty Acid Analysis

Total fatty acids in seeds were quantified by gas chromatography after derivatization to fatty acid methyl esters using pentadecanoic acid (15:0) as internal standard (Browse et al., 1986).

### C and N Elemental Analyses

For the elemental analysis of C and N, dried leaf material was analyzed using an Isoprime 100 isotope ratio mass spectrometer coupled to an elemental analyzer (ISOTOPE cube; Elementar Analysensysteme, Germany). The elemental analyzer was calibrated every 30 samples to an acetanilide standard (*C* = 71.09%; *N* = 10.36%) to assure accuracy.

### Metabolite Profiling via GC-MS and LC-MS/MS

Samples were harvested by directly dropping into liquid nitrogen. For each sample, 40 mg of fresh weight was extracted according to Arrivault et al. (2009). The aqueous phase was lyophilized, resuspended in HPLC-grade water, and the same extracts were used for GC-MS and LC-MS/MS analyses (see below). Further evaluations were done using Microsoft Excel and R statistics software (R version 3.4.3 provided by the CRAN project^3^).

For GC-MS, extracts were dried and derivatized for metabolite analysis by gas chromatography-mass spectrometry (GC-MS) according to Fiehn et al. (2000) using a 7200 GC-QTOF (Agilent, United States). Data analysis was conducted with the MassHunter Quantification software (Agilent).

LC-MS/MS was performed on an Agilent 1200 HPLC system coupled to a 6490 triple quadrupole (Agilent) equipped with an electrospray ionization interface. The HPLC was operated as described by Arrivault et al. (2009) with a different buffer A and slight modifications of the HPLC gradient. Chromatographic separation was performed by passing aliquots through a Gemini (C18) 4 mm × 2 mm precolumn (Phenomenex, United States), before separation on a Gemini (C18) 150 mm × 2 mm inner diameter, 5 μm/110Å particle column (Phenomenex) at 35°C using a multistep gradient with online-degassed eluent A (95% 3 mM dihexylammonium acetate in water, 5% methanol) and eluent B (100% isopropanol). The same gradient as in Mettler et al. (2014) was used: 0–5 min, 100% A; 5–15 min, 100–95% A; 15–22 min, 95–90% A; 22–37 min, 90–85% A; 37–40 min, 85–70% A, and maintained for 3 min; 43–47 min, 70–45% A, and maintained for 3 min; 50 min, 10% A, and maintained for 8 min; 58 min, 100% A, and maintained for 8 min. The flow rate was 0.2 ml⋅min^-1^ and was increased to 0.3 ml⋅min^-1^ between 22 and 58 min.

After separation, compounds were ionized by electrospray ionization and detected by a triple quadrupole that was operated in negative ion mode with selected reaction monitoring, using an ion spray voltage of 3000 V, a gas temperature of 210°C and a sheath gas temperature of 400°C. The MassHunter Application software (Agilent) was used for instrument control and data acquisition. Metabolites were quantified by comparison of the integrated MS-Q3 signal peak area with a calibration curve obtained using authentic standards by the MassHunter Quantification software (Agilent).

### Pigment and Protein Determinations

Photosynthesis-associated pigments were extracted from freshly harvested leaf material in ice cold 100% Methanol overnight at 4°C in the dark. Chl *a*, *b*, and carotenoid contents were determined as described in Wellburn and Lichtenthaler (1984) at wavelengths of 470, 653, and 666 nm.

Proteins were extracted from frozen leaf material in a medium containing 50 mM Hepes-KOH (pH 7.5), and 0.1% (v/v) Triton-X-100 with a Heidolph homogenizer and determined according to Bradford (1976) using BSA as a standard.

Proteins in samples for immunoblot analyses were quantified by the amido black method according to Schaffner and Weissmann (1973) and Wieczorek et al. (1990).

### Photosynthesis Measurements

Photosynthesis parameters at PSII were determined fluorometrically according to Schreiber et al. (1986) and Genty et al. (1989) with an Imaging PAM (M-series, Maxi version, Walz, Effeltrich, Germany). Induction kinetics of photosynthesis and light saturation curves have been assessed according to the routines implemented in the Imaging PAM software. The plants were dark-adapted for 30 min prior to induction with actinic light for 5 min. Care was taken that the light saturation curves of photosynthesis were measured immediately after induction. The duration at each PFD was 20 s starting with the lowest PFD. From the quantum efficiency of PSII photochemistry (ΦPSII) electron transport rates (ETR) can be estimated according to Genty et al. (1989). Since neither leaf absorptance nor antenna cross sections of the photosystems and excitation energy distribution between them were determined, ETR is expressed in relative terms.

### Thylakoid Membrane Isolation, Quantification of Photosynthetic Complexes, and Chl-*a* Fluorescence Emission Analysis at 77K

Thylakoid membranes were isolated according to Schöttler et al. (2004). The contents of PSII and of the Cyt b_6_/f complex per Chl were determined from difference absorption signals of Cytochromes b_559_, f and b_6_ as described in Schmitz et al. (2012). Difference absorbance spectra were deconvoluted using reference spectra and difference extinction coefficients as in Kirchhoff et al. (2002). PSII contents were calculated from the sum of the high- and low-potential difference absorbance signals of Cyt b_559_ (Lamkemeyer et al., 2006). The content of redox-active PSI per Chl was determined from light-induced difference absorption changes of P_700_, the PSI reaction center Chl-*a* dimer. Isolated thylakoids equivalent to 50 μg Chl⋅ml^-1^ were solubilized with 0.2% (w/v) n-dodecyl-β-D-maltoside in the presence of 10 mM sodium ascorbate as electron donor and of 100 μM methylviologen as electron acceptor. P_700_ was oxidized by the application of a saturating light pulse (2000 μmol photons⋅m^-2^⋅s^-1^ red light, 300 ms duration). Measurements were done using the Dual-PAM instrument (Walz, Effeltrich, Germany) in its PC-P_700_ version (Schöttler et al., 2007). Afterwards, based on the known Chl content per leaf area, all complex contents were re-normalized to a leaf area basis.

Chl *a* fluorescence emission spectra at 77K were measured on thylakoids equivalent to 10 μg Chl⋅ml^-1^ using an F-6500 fluorometer (Jasco GmbH, Groß-Umstadt, Germany). The samples were excited at 430 nm wavelength (10 nm bandwidth), and fluorescence emission spectra were recorded between 655 and 800 nm with a bandwidth of 1 nm and a scanning speed of 200 nm. Ten spectra were averaged and corrected for the instrumental response of the photomultiplier.

### Statistical Evaluation of Experimental Data

The data are expressed as mean values ± standard error of the mean (SE) of the indicated number of independent measurements. Significant differences between more than two physiological data sets were analyzed using one-way ANOVA combined with the *post hoc* Tukey-Kramer test, which allows the comparison of unequal sample sizes and identifies values which are significantly different (Ludbrook, 1998). For data plotting and fitting, SigmaPlot10.0 for Windows (Systat Software Inc.) was used.

## Results

### Knockout Mutants of the XPT Lack Any Visible Phenotype

**FIGURE 1:**
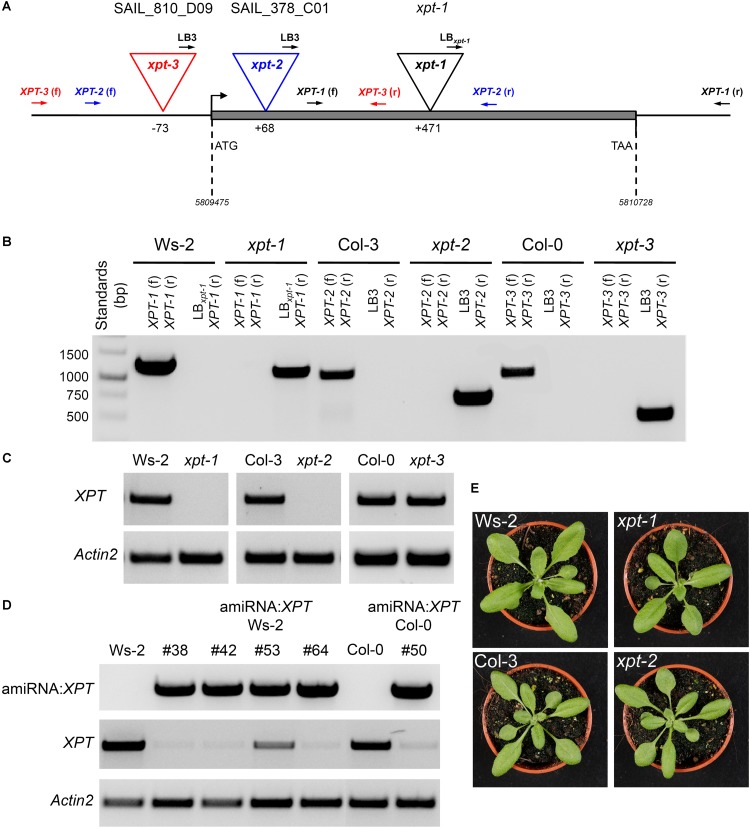
Molecular characterization of *XPT* T-DNA insertion mutants and amiRNA:*XPT* lines in the Ws-2 or Col-0 background. **(A)** *XPT* gene structure with the individual T-DNA insertions as well as the locations of primer pairs for the identification of the inserts. **(B)** Amplification pattern of genomic DNA fragments using gene specific and/or T-DNA left border (LB) primers as indicated. **(C)** *XPT* expression based on RT-PCR fragments generated from RNA isolated from wild-type plants or T-DNA insertion mutants as indicated. As a control, *Actin2* was used. **(D)** Molecular analyses of amiRNA:*XPT* insertion lines in the Ws-2 or Col-0 background. The upper panel shows fragments of the amiRNA:*XPT* construct amplified by PCR on genomic DNA of the individual lines. In the lower panels RT-PCR fragments of the *XPT* or *actin2* (control) are displayed for the individual lines. **(E)** Compared to the respective wild-type plants the *xpt-1* and *xpt-2* mutant alleles lacked any visible phenotype when grown under SL-conditions (PFD = 150 μmol⋅m^-2^⋅s^-1^) in the long-day.

Three mutant alleles with T-DNA insertions in the *XPT* gene of *A. thaliana* have been isolated (**Figure 1A**). The *xpt-1* mutant allele (Ws-2 background) carries a T-DNA insertion at position 471 downstream of the start ATG. Both Southern blot analyses and backcrosses to Ws-2 wild-type plants indicated a single insertion in the genome of *xpt-1* (Eicks, 2004*).* Furthermore two SAIL-lines were found that contain T-DNA insertions at position 68 downstream and at position 73 upstream of the start ATG (*xpt-2* and *xpt-3*; **Figure 1A**). The *xpt-2* line has been established in the Col-3 background and carries, in addition to the insertion in the *XPT*-gene, point mutations in a pectin esterase gene (*QUARTET; QRT*; At5g55590) resulting in pollen tetrades even after maturation of male gametophytes without affecting vegetative or generative development of the plants (Preuss et al., 1994; Francis et al., 2006). Finally the *xpt-3* mutant allele (Col-0 background) carries a T-DNA insertion in the promoter/5’ UTR region of the *XPT* gene, 73 bp upstream of the start codon. The homozygous T-DNA insertions in the *XPT*-gene were confirmed by PCR on genomic DNA using a variety of gene-specific primers as well as T-DNA border primers (**Figure 1B** and **Supplementary Table 1**). Qualitative RT-PCR revealed the absence of *XPT* transcripts only in the *xpt-1* and *xpt-2* mutant alleles, but not in *xpt-3* (**Figure 1C**). In order to obtain plants with largely diminished *XPT* expression amiRNA:*XPT* lines have been generated in the background of WS-2 as well as Col-0 (**Figure 1D**). These lines have been deployed instead of *xpt-3* for further studies.

According to the ‘electronic fluorescent protein browser’ (*efp*-browser^4^; Winter et al., 2007) and data by Eicks et al. (2002), the XPT is ubiquitously expressed in *A. thaliana*. Although the XPT represents a single copy gene, transgenic lines carrying a knockout of the *XPT* gene by T-DNA insertion or a knockdown by amiRNA:*XPT* lack any pronounced phenotype in the vegetative state (**Figure 1E**).

However, in the generative state, the initially isolated *xpt-1* mutant in the Ws-2 background developed larger seeds in shorter siliques, which was accompanied by an increase in seedling size. Neither *xpt-2* nor amiRNA:*XPT* lines in the Ws-2 or Col-0 background showed any similar changes in seed development suggesting that this generative phenotype is specific for *xpt-1* and cannot be generalized. **Supplementary Document 1 (Doc S1)** comprises detailed analyses of vegetative and generative development of XPT knockout and amiRNA:*XPT* plants, including productivity parameters such as seed oil and protein contents and compositions. Apart from *xpt-1* neither of the other lines with impaired XPT function showed marked differences in any of these parameters.

### A Double Knockout of the XPT and the TPT Leads to Growth Retardation and Impaired Photosynthesis

As the XPT exhibits substantial TP/P_i_ transport capacities (Eicks et al., 2002) it might, together with the TPT, contribute to the export of photoassimilates from chloroplasts in the light. In order to test this hypothesis and to block both ways of TP transport, the *xpt-1* mutant was crossed to both alleles of the *tpt* mutants (i.e., *tpt-1* and *tpt-2*). It ought to be mentioned here, that *tpt-1* represents a knockdown mutant, which contains residual transcripts of the *TPT* (Schneider et al., 2002), whereas in *tpt-2* expression of the *TPT* is abolished (Schmitz et al., 2012). Moreover, the same amiRNA:*XPT* construct used for the repression of *XPT* expression in wild-type plants was also transferred into the *tpt-2* mutant allele.

**FIGURE 2:**
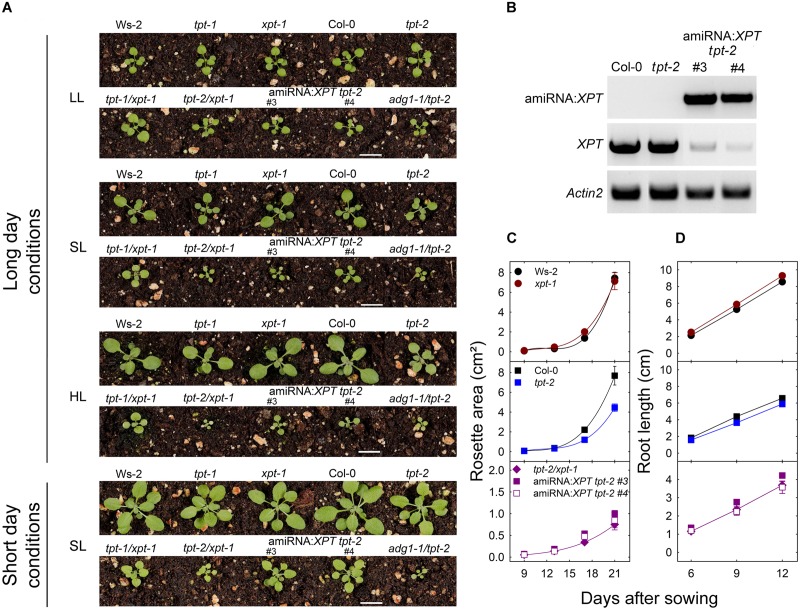
Characterization of mutant and amiRNA plants with defects in the TPT and XPT. In **(A)** phenotypes of wild-type plants, single or double mutants impaired in the TPT and XPT, as well as amiRNA:*XPT* overexpressors in the *tpt-2* background are shown. As an additional control *adg1-1/tpt-2* plants are displayed. The plants were grown either under LL- (PFD = 30 μmol⋅m^-2^⋅s^-1^), SL- (PFD = 150 μmol⋅m^-2^⋅s^-1^), or HL-conditions (PFD = 300 μmol⋅m^-2^⋅s^-1^) in the long-day, or under SL-conditions in the short-day. **(B)** RT-PCR-amplified transcripts of amiRNA:*XPT* (upper panel), *XPT* (middle panel), and, as a control, *Actin2* (lower panel) in Col-0 wild-type plants, the *tpt-2* single mutant, and the amiRNA:*XPT* overexpressing lines #3 and #4 in the *tpt-2* background. The space bar represents 1 cm. In **(C,D)** growth rates of leaf rosettes and roots, respectively, are shown for Ws-2 (closed black circles), Col-0 (closed black squares), *xpt-1* (closed dark red circles), *tpt-2* (closed blue squares), *tpt-2/xpt-1* (closed purple diamonds), and the amiRNA*:XPT* lines in the *tpt-2* background #3 (closed purple squares) and #4 (open purple squares). Note the different scales of the y-axis in the lower part of the graphs. The plants in **(C)** were grown in soil under SL-conditions in the long-day. Root growth in **(D)** was assessed on 1/2MS agar plates under SL-conditions in the-long day. A statistical analysis of the growth parameters is contained in **Doc S1, Table 1**.

**FIGURE 3:**
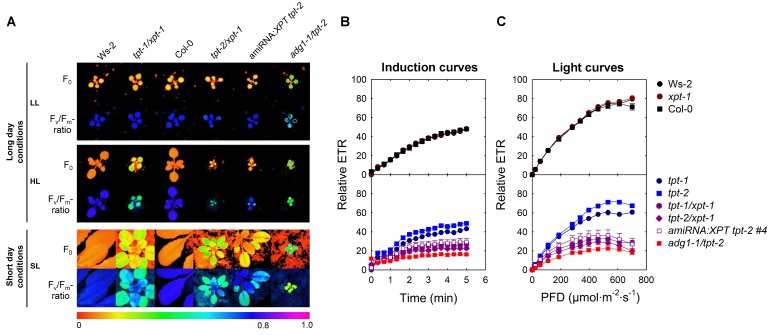
Photosynthetic characteristics of wild-type (Ws-2, Col-0) and mutant plants defective in the TPT and XPT. In **(A)** false-colour images of F_o_ and the F_v_/F_m_-ratios are displayed for plants grown for 20 days under LL- (PFD = 30 μmol⋅m^-2^⋅s^-1^) or for 12 days under HL-conditions (PFD = 300 μmol⋅m^-2^⋅s^-1^) in the long-day, or for 43 days under SL-conditions (PFD = 150 μmol⋅m^-2^⋅s^-1^) in the short-day. For the latter details of the fluorescence distribution or of F_v_/F_m_-ratios are depicted (lower panel). In **(B,C)** induction and light curves, respectively, of relative ETR are shown for wild-type plants, the *xpt-1*, *tpt-1*, and *tpt-2* single mutants as well as for *tpt-1/xpt-1*, and *tpt-2/xpt-1* double mutants and an amiRNA:*XPT* overexpressor in the *tpt-2* background grown under HL-conditions. The actinic light during the induction of photosynthesis was set to a PFD of 281 μmol⋅m^-2^⋅s^-1^. As an additional control the *adg1-1/tpt-2* double mutant was included in this experiment.

To our great surprise *tpt-1[2]/xpt-1* and the amiRNA:*XPT tpt-2* plants exhibited pronounced growth and photosynthesis phenotypes (**Figures 2**, **3**) similar to those described for *adg1-1/tpt-1[2]*. The latter has been extensively analyzed over the past decade (Häusler et al., 2009; Heinrichs et al., 2012; Schmitz et al., 2012, 2014; Häusler et al., 2014a; Hölscher et al., 2016). Hence we included *adg1-1/tpt-2* in most experiments as an additional control. Furthermore the *tpt-1* and *tpt-2* single mutants that have been characterized by Schneider et al. (2002) and Schmitz et al. (2012) will also serve as additional controls, as they were used for crosses with *xpt-1*.

As is shown in **Figure 2A** the dramatic growth retardation of the *tpt-1[2]/xpt-1* double mutants was dependent on the PFD of the growth light and occurred only under HL- or SL-, but not under LL-conditions. Two amiRNA:*XPT tpt-2* lines with a large reduction in *XPT-*specific transcripts (**Figure 2B**) showed a similar growth behavior as the *tpt-1[2]/xpt-1* double mutants indicating that combined deficiencies in TPT and XPT functions are indeed responsible for this phenotype.

Growth properties of shoots (**Figure 2C**) and roots (**Figure 2D**) were quantitatively assessed. Plants with a combined defect in TPT and XPT exhibited a clearly diminished shoot growth by approximately 90% when grown under HL-conditions in the long-day (**Figure 2C**). In addition, the *tpt-2* mutant also showed slightly diminished growth rates as compared to Col-0. When both TPT and XPT were impaired, root growth was inhibited between 40 and 70% (**Figure 2C**). A statistical analysis of the growth analyses is contained in **Supplementary Document S2 (Doc S2) Table 1**.

Similar to growth limitations during the vegetative phase, generative development was also severely impaired in plants with combined lesions in TPT and XPT (**Table 1**). For instance, inflorescences were about 25% shorter in height in the *tpt-2/xpt-1* double mutant as compared to Col-0. There were 57% or 70% less siliques compared to Col-0 or Ws-2, respectively, and a severely diminished seed weight or seed number per plant in the double mutant, whereas the specific seed weight (i.e., per 100 seeds) or the seed area was not affected. Similar to the vegetative development, constraints in generative growth were less pronounced in the amiRNA:*XPT tpt-2* lines (**Table 1**), which might point at a leakiness in *XPT* expression (compare **Figure 2B**). A statistical analysis of the data is contained in **Doc S2, Table 1**.

Besides of growth retardation, the *tpt-1[2]/xpt-1* double mutants and amiRNA:*XPT tpt-2* plants exhibited a HCF-phenotype in the dark-adapted state when grown under HL- or SL-conditions, whereas the single mutants were not affected (**Figure 3A**). The increase in F_o_ was accompanied by a concomitant decrease in the F_v_/F_m_-ratio, similar to the *adg1-1/tpt-2* double mutant, and was also reflected in a lower relative ETR (**Figure 3B**), in particular at high PFDs (**Figure 3C**, **Supplementary Table S2**).

The differences in photosynthetic performance between the lines was supported by the composition of photosynthetic pigments, leaf content of protein, and specific leaf fresh weights (fw) of HL-grown plants (**Table 2**). Contents of Chl and protein dropped to the lowest levels relative to the wild type in *tpt-2/xpt-1* (i.e., a decrease by 44 and 36% for Chl and protein, respectively). Protein levels were thus less affected than Chl contents, but in general, their changes correlated closely with each other. Moreover, contents of carotenoids were also less diminished compared to Chl contents, suggesting an enhanced requirement for energy dissipation in form of heat. Furthermore, the decrease in the specific leaf fw might point at lower water contents and/or a lighter general structure, such as cell walls, vasculature or thinner leaves.

**Table 2:** Pigment and protein contents as well as specific leaf fresh weights of wild-type, mutant, and amiRNA plants defective in the TPT and/or XPT.

Plant line	Chl content	Carotenoid content	Chl *a/b*-ratio	Protein content	Specific fw
			
	(mg⋅m^-2^)			(g⋅m^-2^)	
Ws-2	224.0 ± 9.6	33.73 ± 1.44	3.48 ± 0.05	3.47 ± 0.13	210.69 ± 4.86
*xpt-1*	244.8 ± 8.1	32.99 ± 1.29	3.32 ± 0.05	3.51 ± 0.14	251.02 ± 10.70
*tpt-1*	187.3 ± 6.8	31.50 ± 0.40	3.38 ± 0.06	3.22 ± 0.14	179.76 ± 3.87
*tpt-1/xpt-1*	147.9 ± 5.3	32.27 ± 0.62	3.27 ± 0.17	2.76 ± 0.11	168.18 ± 2.85
Col-0	238.1 ± 7.5	32.18 ± 1.54	3.42 ± 0.05	3.05 ± 0.16	232.10 ± 5.29
*tpt-2*	206.3 ± 4.3	34.41 ± 0.89	3.45 ± 0.09	2.88 ± 0.14	203.03 ± 3.53
*tpt-2/xpt-1*	134.0 ± 2.8	27.66 ± 0.80	2.95 ± 0.05	1.95 ± 0.13	112.77 ± 5.46
amiRNA:*XPT tpt-2* #3	161.1 ± 18.0	28.31 ± 1.82	3.01 ± 0.10	2.24 ± 0.06	128.35 ± 12.14
amiRNA:*XPT tpt-2* #4	163.7 ± 14.8	31.00 ± 1.37	3.25 ± 0.06	2.56 ± 0.17	131.36 ± 7.04
*adg1-1/tpt-2*	108.4 ± 4.9	25.35 ± 0.73	2.54 ± 0.07	1.92 ± 0.10	135.51 ± 3.21


*The plants were grown for 15 days after sowing under HL-conditions (PFD = 300 μmol m^-*2*^ s^-*1*^) in the long-day. Pigment and protein contents are expressed on a leaf area basis. The specific leaf fresh weight allows a conversion from a leaf area to a leaf weight basis. For the Chl *a/b* ratios Chl *a* and *b* were determined separately and the quotient calculated for each replicate. The data represent the mean ± SE of *n* = 4 to 20 replicates. For Chl and carotenoid contents of HL-grown amiRNA *XPT tpt-2* lines the number of replicates was lower (*n* = 2 to 3). A statistical analysis of the data is contained in **Doc S2, Table 3**.*

The Chl *a/b*-ratio which provides information on the relative abundance of PSII versus PSI, in particular of their LHCs (Ort, 1986), was slightly decreased from 3.42 in Col-0 to 2.95 in *tpt-2/xpt-1*, but not in *tpt-1/xpt-1* or the amiRNA:*XPT tpt-2* lines. The most pronounced decrease in the Chl *a/b*-ratio to a value of 2.54 was observed for *adg1-1/tpt-2.* Under LL-conditions differences between the plants were either less marked or absent (**Supplementary Table 3**). A statistical analysis of the data is contained in **Doc S2, Table 3B**.

### What Are the Underlying Reasons for Similar Growth- and Photosynthesis Phenotypes Between *tpt-1[2]/xpt-1* and *adg1-1/tpt-1[2]*?

In order to find satisfactory explanations for the phenotypical similarities in both double mutants, here we intend to dissect various aspects of metabolism and photosynthesis in a holistic way. These analyses will start from primary effects on metabolism, like (i) changes in starch and sugar levels during the course of a day, (ii) perturbations in the metabolome including phosphorylated intermediates of primary metabolism and amino acids. Finally (iii) a rescue of the growth and photosynthesis phenotypes of *tpt-1[2]/xpt-1* by externally fed sugar and (iv) aspects of the HCF-phenotype will be addressed. In most cases the *adg1-1/tpt-2* double mutant will serve as an additional control. Unless stated otherwise, all of these analyses were performed on plants grown under HL-conditions in the long-day.

### Diurnal Changes in Starch and Sugar Levels Suggest Accelerated Day-Time Starch Turnover in *tpt-1[2]/xpt-1* Double Mutants

Perturbations in carbohydrate metabolism would be expected as direct consequences when the ‘day path of carbon’ is completely blocked. Diurnal assessments of starch and sugar levels are hence the most appropriate way to test this assumption.

**FIGURE 4:**
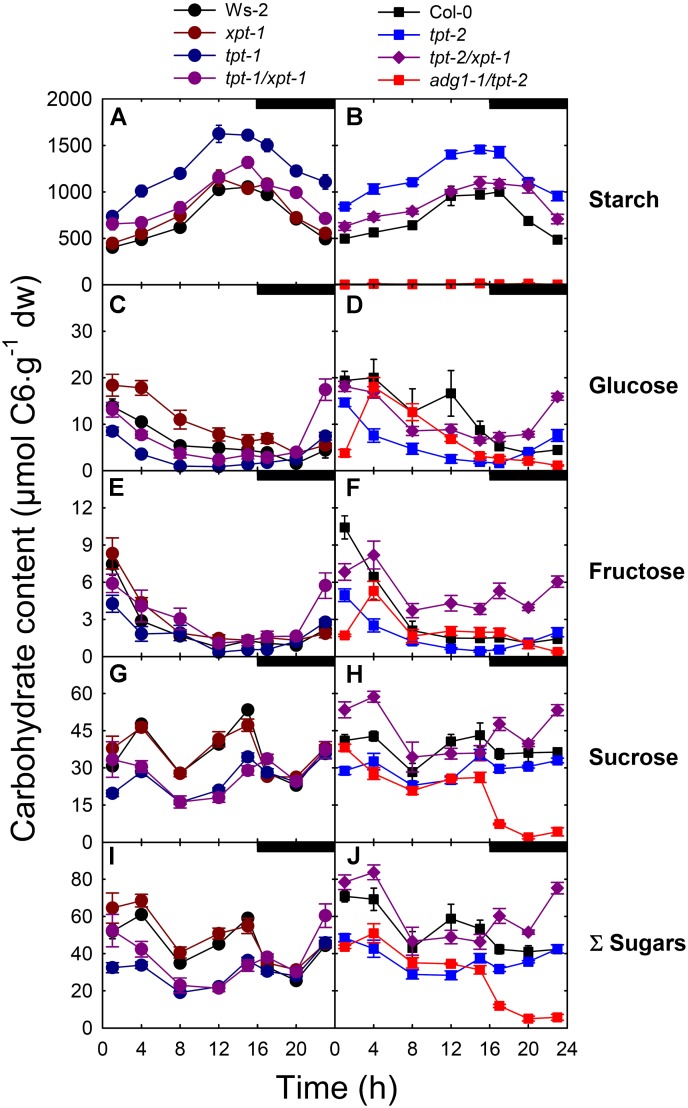
Diurnal changes in carbohydrate contents in leaves of wild-type and mutant plants. Contents of starch **(A,B)**, the soluble sugars Glc **(C,D)**, Fru **(E,F)**, Suc **(G,H)** were determined and the sum the soluble sugars **(I,J)** calculated for Ws-2 (closed black circles), *tpt-1* (closed dark blue circles), *xpt-1* (closed dark red circles), *tpt-1/xpt-1* (closed dark purple circles), Col-0 (closed black squares), *tpt-2* (closed blue squares), *tpt-2/xpt-1* (closed dark purple diamonds), and *adg1-1/tpt-2* (closed red squares). The plants were grown in soil under HL-conditions (PFD = 300 μmol⋅m^-2^⋅s^-1^) in the long-day. The data represent the mean ± SE of *n* = 5 replicates. A statistical analysis is contained in **Doc S2, Table 4**.

Apart from the line *adg1-1/tpt-2*, starch accumulated steadily from the beginning towards the end of the light period in the other lines investigated (**Figures 4A,B**). As expected from previous reports (Schneider et al., 2002; Schmitz et al., 2012), starch levels in both *tpt-1[2]* single mutant alleles were almost doubled for most of the day and night compared to the respective wild-type backgrounds. Neither *xpt-1* nor the *tpt-1[2]/xpt-1* lines showed large differences in starch contents compared to wild-type plants, suggesting that in the double mutants starch is either mobilized more efficiently during the day or that its biosynthesis is limited due to diminished photosynthesis rates.

The limitation on TP export in both *tpt-1[2]* mutant alleles corresponded well with lower levels of Glc, Fru and Suc during the light period (**Figures 4C–H**). In *tpt-1[2]* soluble sugar levels recovered slightly starting from the middle of the light period onwards to the end of the dark period, probably due to starch mobilization (**Figures 4I,J**) and thus resembled earlier observations (Schneider et al., 2002; Schmitz et al., 2012). Hence, the starch levels were inversely correlated with sugar contents in both *tpt-1[2]* single mutants. The *xpt-1* single mutant exhibited an increase in Glc contents during the first half of the light period, while the time courses for Fru and Suc contents were identical to the wild type (**Figures 4C,E,G**). The time courses of individual soluble sugars were more complex in the *tpt-1[2]/xpt-1* double mutants compared to the wild-type or single mutant plants, and appeared to be dependent on the strength of the allele. A common feature of both double mutant lines was the steep increase in soluble sugar contents towards the end of the dark period (**Figures 4I,J**), suggesting an enhanced mobilization of transitory starch. In *adg1-1/tpt-2* soluble sugar levels dropped rapidly during the dark period (**Figure 4J**), because of the lack of transitory starch, but were also replenished rapidly during the early light period. This rapid replenishment was more pronounced for Suc (**Figure 4H**) as compared to Glc (**Figure 4F**) or Fru (**Figure 4D**).

In summary, with the exception of Glc, soluble sugar levels in *tpt-1[2]/xpt-1* were higher than in *adg1-1/tpt-2* for most of the light period, and particularly in the dark. Hence a limitation on sugar contents as reason for the common growth- and HCF-phenotype of *tpt-1[2]/xpt-1* and *adg1-1/tpt-2* appears less likely from this experiment. Moreover, lower starch levels as compared to the *tpt-1[2]* mutants and the steep increase in soluble sugars during the dark period suggest an increased starch turnover in the light and dark to compensate for the deficiency in TP export. A statistical analysis of the data is contained in **Doc S2, Table 4**.

### Metabolite Profiles Reveal Accumulations of Pentose Phosphates and Maltose as Well as Perturbations in Organic Acids in *tpt-1[2]/xpt-1* Double Mutants

**FIGURE 5:**
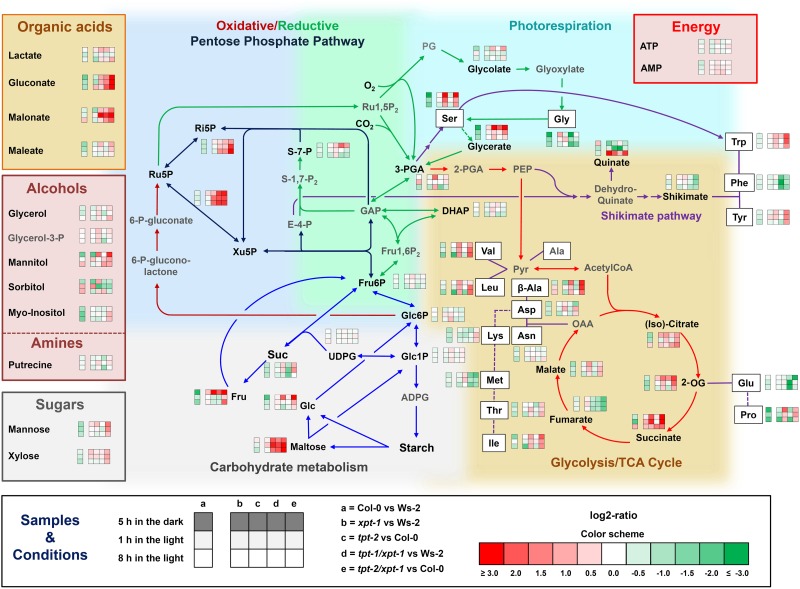
Metabolome analyses of wild-type and mutants plants. Wild-type and mutant plants impaired in the TPT and/or XPT were grown for 3 weeks under HL-conditions (PFD = 300 μmol⋅m^-2^⋅s^-1^) in the long-day. Samples for metabolite extractions were taken either at about the middle of the dark period (i.e., 5 h in the dark), at the beginning of the light period (i.e., 1 h in the light), or in the middle of the light period (i.e., 8 h in the light). The small heatmaps for each metabolite and the color scheme for the log2-ratios are defined under ‘Samples and Conditions’. The background colors in the metabolic sketch highlight aspects of various pathways, i.e., light green, reactions specific for the Calvin-Benson cycle including initial reactions of photorespiration; light blue, reactions of the OPPP and the overlap with the Calvin-Benson cycle; light cyan, reactions of photorespiration; light brown, glycolysis and TCA cycle as well as aspects of amino acid biosynthesis; light grey, carbohydrate metabolism. The colors of the arrows incidate more specifically reactions of individual pathways or groups of pathways, i.e., green, Calvin-Benson cycle and photorespiration; dark red, oxidative part of the OPPP; dark blue, regenerative part of the OPPP and Calvin-Benson cycle; blue, carbohydrate metabolism; red glycolysis and TCA; purple, amino acid metabolism including the shikimate pathway. The metabolites in black letters have been determined by GC-MS or LC-MS/MS whereas the grey-lettered metabolites indicate important intermediates that have not been determined. All phosphorylated metabolites, ATP and AMP, as well as Asn and Trp were determined by LC-MS/MS, all remaining metabolites were determined by GC-MS. Please note that, for the sake of clarity, compartmentation has been omitted. The data represent the mean ± SE of *n* = 3 to 5 replicates. The metabolite contents at each time point of the day are contained in **Supplementary Tables 4**, **5** with a statistical analysis in **Doc S2, Tables 5**, **6**.

The assessment of metabolic pattern in wild-type and mutant plants impaired in the TPT and XPT can provide information on perturbations in certain branches of metabolism and might hence allow drawing conclusions on the functional role of the XPT. By means of GC-MS and LC-MS/MS a total of 48 individual intermediates of primary metabolism were identified (**Figure 5** and **Supplementary Tables 4**, **5**). Of these metabolites only a small portion was significantly affected in mutant compared to wild-type plants, when three time points of harvest during the course of the day were compared (i.e., about the middle of the night [5 h in the dark], beginning of the day [1 h in the light], and middle of the day [8 h in the light]).

The LC-MS/MS data revealed a dramatic increase in the content of the Calvin-Benson Cycle intermediates Xu5P/ribulose 5-P (Ru5P) in both double mutants, which was most prominent towards the middle of the light period, suggesting a limitation on transport from one compartment to the other, or a decrease in pentose 5-P consumption. Surprisingly, during the light period the pentose phosphates showed a trend of an increase also in *tpt-2*, but not, as might be expected, in *xpt-1*. Changes in further Calvin-Benson Cycle intermediates, such as 3-PGA, DHAP, and sedoheptulose 7-P (S-7-P) were not apparent for *tpt-1[2]/xpt-1*. There was, however, a significant increase in 3-PGA in *tpt-2* and S-7-P in *tpt-1/xpt-1* at the end of the dark period, and of ribose 5-P (Ri5P) in *tpt-2/xpt-1* in the middle of the light period (**Figure 5** and **Supplementary Tables 4A,C**). A statistical analysis of the data is contained in **Doc S2, Table 5**.

Of the soluble sugars the disaccharide maltose, which derives entirely from starch degradation via β-amylase, was significantly increased in both double mutants and in *tpt-2* in the middle of the dark period, suggesting high rates of starch mobilization and/or a limitation on maltose export from the chloroplast including further metabolism (**Figure 5** and **Supplementary Table 5**). The most pronounced significant increase in maltose levels was observed in the *tpt-2/xpt-1* double mutant, containing the strong *tpt-2* allele, at the beginning of the light period. Relative to Col-0 the content of maltose was increased by a factor of 35. Although maltose levels dropped towards the middle of the light period, they were still markedly enhanced by a factor of 17.3 in *tpt-2/xpt-1* compared to Col-0. At the end of the dark period there was also an increase in Glc and Fru in *tpt-2/xpt-1*, supporting the enzymatic sugar determinations depicted in **Figure 4**.

Contents of organic acids like gluconate or malonate were increased in both double mutant alleles and in *tpt-2* (at least at one time point of harvest; **Figure 5**). It is conceivable that gluconate is a derivitization-degradation product of the OPPP intermediate 6-P-gluconate (Baxter et al., 2007). The tricarbonic acid (TCA) cycle intermediates 2-oxoglutarate (2-OG) and succinate responded strongly with significantly increased contents only in *tpt-2/xpt-1* and, for succinate, in *tpt-2*. In contrast fumarate, the downstream product of succinate, exhibited a significant decrease *tpt-2/xpt-1* at all three time points of harvest and also in *tpt-2* at the middle of the light period.

Amino acids lacked consistent pattern of changes in the double mutants compared to the single mutants, in particular if the relatively large differences between the two wild-type accessions Ws-2 and Col-0 were also considered. The accumulation of 2-OG, which represents the precursor for *de novo* amino acid synthesis via the GS/GOGAT cycle was not accompanied by a similar increase in Glu.

Contents of other metabolites remained either unchanged or showed inconsistent pattern of changes.

### Amino Acid Profiles Reveal a Drop in Gly in *tpt-1[2]/xpt-1* and *adg1-1/tpt-2* Double Mutants

**FIGURE 6:**
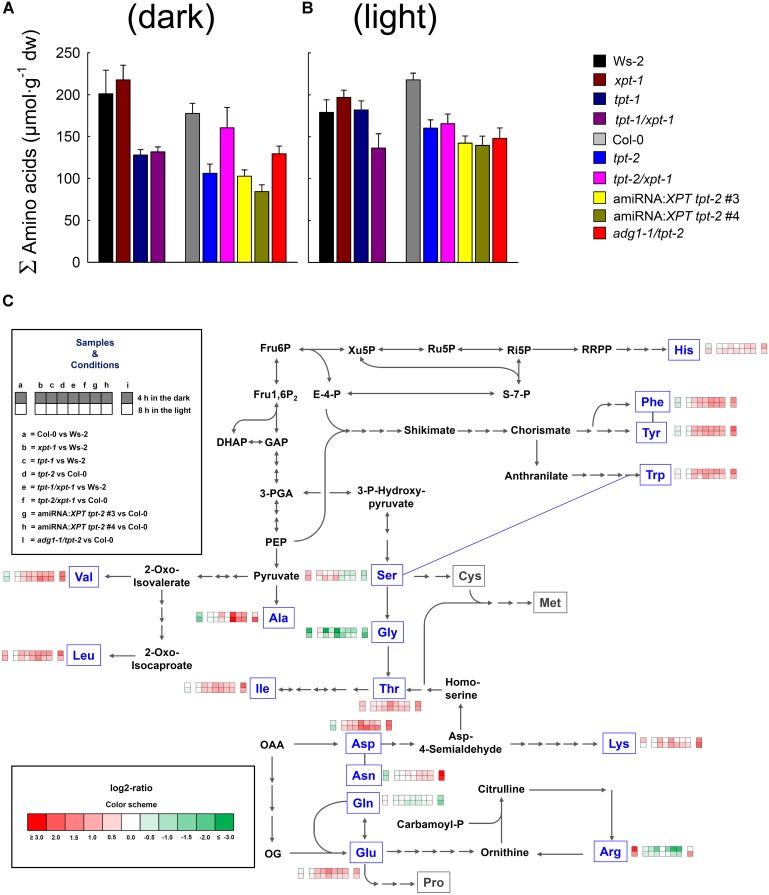
Contents of soluble amino acids in leaves of wild-type and mutant plants. Amino acid contents were determined by HPLC in samples extracted from rosette leaves harvested after 4 h in the dark (i.e., the middle of the dark period) or 8 h in the light (i.e., the middle of the light period). The plants were grown in soil under HL-conditions (PFD = 300 μmol⋅m^-2^⋅s^-1^) in the long-day. Contents of total amino acids (expressed as sum of the individual amino acids) are shown in **(A)** and **(B)** for the middle of the dark- and light period, respectively. In the metabolic sketch **(C)** the major paths of amino acid biosynthesis are illustrated. The framed amino acids in blue letters have been determined by HPLC and contain heatmaps based on log2-ratios (indicated by the color sheme) of relative changes in their content (i.e., as percent of the summed amino acids) compared to wild-type plants. The heatmap pattern is defined under ‘Samples and Conditions’. The data represents the mean ± SE of *n* = 4 to 5 independent replicates. A detailed list of absolute and relative amino acid contents is contained in **Supplementary Table 6**. A statistical analysis is contained in **Doc S2, Table 7**.

Like carbohydrates, amino acids are major constituents in plant cells and fulfill a large spectrum of different tasks (Häusler et al., 2014b; Pratelli and Pilot, 2014; Hildebrandt et al., 2015; Galili et al., 2016). Perturbations in their contents might hence point at metabolic constraints. The patterns of proteinogenic amino acids were assessed with the aid of reversed phase HPLC in rosette leaves harvested from all relevant mutant, wild-types, and amiRNA:*XPT tpt-2* lines 4 h in the dark or 8 h in the light (**Figure 6** and **Supplementary Table 6**). The metabolic sketch is based essentially on amino acid biosynthesis pathways implemented in the KEGG data base^5^.

**FIGURE 7:**
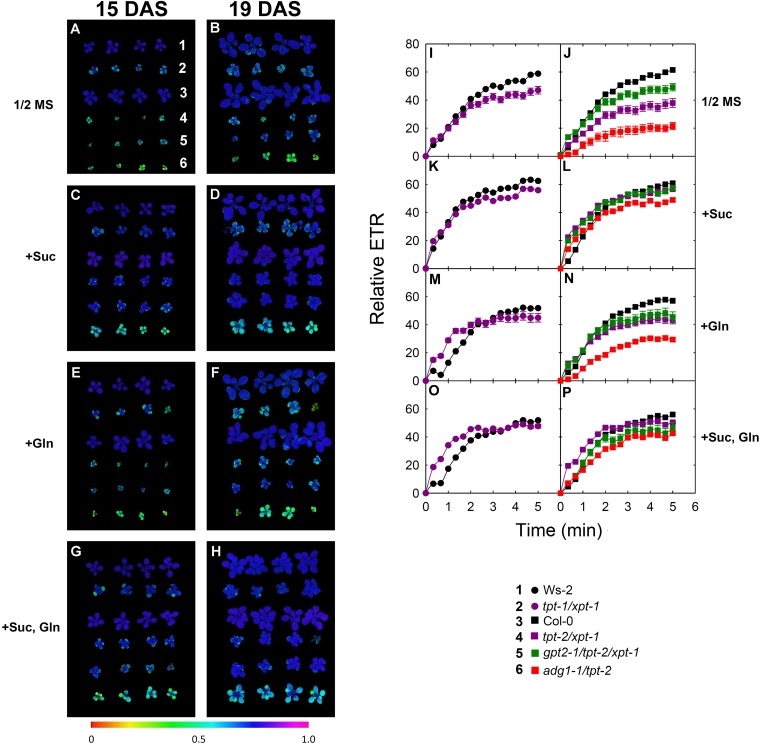
Photosynthesis parameters of wild-type and mutant plants grown on agar with or without substrates. Wild-type plants and double mutants impaired in the TPT and XPT as well as triple mutants with an additional defect in GPT2 were grown on 1/2 strength MS agar in the absence or presence of 50 mM Suc, 2 mM Gln, either alone or in combination. The plants were grown under HL-conditions (PFD = 300 μmol⋅m^-2^⋅s^-1^) in the long-day. As an additional control the *adg1-1/tpt-2* double mutant was included in this experiment. The left panel **(A–H)** shows images of the F_v_/F_m_-ratio after the plants were dark-adapted for 30 min at day 15 **(A,C,E,G)** or day 19 **(B,D,F,G)** after sowing. The numbers in **(A)** indicate the individual lines with Ws-2 (1), *tpt-1/xpt-1* (2), Col-0 (3), *tpt-2/xpt-2* (4), *gpt2-1/tpt-2/xpt-1* (5), and *adg1-1/tpt-2* (6). The right panel shows induction kinetics of photosynthetic ETR of both wild types **(I,K,M,O)** and the individual mutant lines **(J,L,N,P)** at day 19 after sowing. The data represent the mean ± SE of *n* = 5 replicates. In some cases error bars were smaller than the symbol size. The color-code for the individual lines is given in the Figure. Additional information on this experiment is contained in **Supplementary Figures 5**, **6**. A statitical analysis is contained in **Doc S2, Tables 11**, **12**.

The contents of total amino acids showed some variation not only between the lines, but also between both time points of harvest (**Figures 6A,B**). After 4 h in the dark, amino acid contents in both *tpt-1[2]* mutants as well as in *tpt-1/xpt-1* and both amiRNA:*XPT tpt-2* lines were almost halved compared to the respective wild-type plants (**Figure 6** and **Supplementary Table 6A**). Surprisingly, in the *tpt-2/xpt-1* double mutant, amino acids recovered almost to wild-type levels compared to the *tpt-2* single mutant. A slight increase in amino acid levels relative to *tpt-2* was also evident for *adg1-1/tpt-2*. For *tpt-2* and both amiRNA:*XPT tpt-2* lines as well as for the *adg1-1/tpt-2* double mutant statistically significant differences emerged for a drop in total amino acid contents in the middle of the day (**Figure 7B**, **Supplementary Table 6B** and **Doc S2, Table 7B**). Larger differences in amino acid contents between both wild-type backgrounds were, however, not significant.

The above data suggest that a lesion in the TPT results in lower total amino acid levels, predominantly during the dark (**Figure 6A**). Their recovery observed for the strong double mutant allele *tpt-2/xpt-1* at the same time was probably due to a massive retardation in growth, similar to *adg1-1/tpt-2*. In the middle of the light period amino acid contents recovered to wild-type levels in the weak *tpt-1* allele, but stayed low in the strong *tpt-2* allele, both *tpt-1[2]/xpt-1* double mutants, the amiRNA:*XPT tpt-2* lines as well as *adg1-1/tpt-2*.

The levels of individual amino acids were either expressed in absolute (i.e., per g dry weight; **Supplementary Tables 6A,B**) or relative terms (i.e., as percentage of the summed amino acid content; **Figure 6C** and **Supplementary Tables 6C,D**). In both cases Gly levels were severely depleted in the light and dark in *tpt-1[2]/xpt-1* as well as in the amiRNA:*XPT tpt-2* lines. Interestingly, the *tpt-1[2]/xpt-1* mutants had this feature in common with *adg1-1/tpt-2*. In addition, both *tpt-1[2]* mutant alleles exhibited diminished Gly contents only in the dark period, whereas Gly contents recovered to wild-type like levels in the light. As Gly and Ser play prominent roles in the photorespiratory carbon and nitrogen cycle, it was tested whether the average rate of relative ETR determined for the individual lines correlated with the contents of both amino acids. Indeed, there was a positive correlation of relative ETR with Gly-, but not with Ser contents (**Supplementary Figure 1**).

Of the major amino acids Asn levels were strongly increased in *adg1-1/tpt-2* (**Figure 6C** and **Supplementary Tables 6A,B**). Changes in the contents of other individual amino acids between the lines were often masked by similar differences between both wild-type accessions. For instance, Arg contents were increased 3- to 5-fold in Col-0 relative to Ws-2, but also dropped relative to Col-0 in *tpt-2/xpt-1* and the amiRNA:*XPT tpt-2* lines, but not in *adg1-1/tpt-2*. Other amino acids were altered only in individual lines; i.e., increased Ala contents in *tpt-2/xpt-1*. Again this effect might be due to depletion in Ala in Col-0 relative to Ws-2.

Most minor amino acids (including aromatic and branched-chain amino acids as well as Lys), showed a general trend of an increase in the mutant and transgenic lines, with the exception of *xpt-1*, relative to the wild-type plants (**Supplementary Tables 6A,B**). This trend was even more marked, when the amino acids contents were compared on a percentage basis (**Figure 6C** and **Supplementary Tables 6C,D**). A statistical analysis of the data is contained in **Doc S2, Table 7**.

### Carbon/Nitrogen-Ratios Were Increased in Both *tpt-1[2]* Mutants, but Recovered in *tpt 1[2]/xpt-1* Double Mutants

An elemental analysis focusing on total C- and N-levels in leaves of the individual lines ought to provide insights into the relative distribution of both major elements beyond carbohydrate or amino acid levels (**Table 3**). The C/N-ratio in the *tpt-1[2]* single mutants was significantly increased by about 15 to 20% compared to the respective wild-type plants. In both cases the increase was due to a depletion in the abundance of N rather than to an accumulation of C. Interestingly, in the *tpt-1[2]/xpt-1* double mutants the C/N-ratios recovered to levels very similar to the respective wild-type plants. However, a closer inspection of the relative abundance of both elements in the double mutants revealed a slight decrease in C and a small increase in N relative to *tpt-1[2]*, which both contribute to the wild-type like C/N-ratios. As might be expected, the C/N-ratio in the *adg1-1/tpt-2* double mutants dropped by almost 30%, which could be attributed to a substantial loss in C and a gain in N. Moreover, the fw/dw-ratio was markedly increased in *adg1-1/tpt-2*, indicating higher water contents and/or thinner cell walls and the complete loss of starch accumulation (see also Schmitz et al., 2012), whereas fw/dw-ratios of the other mutant and transgenic lines lacked any consistent pattern or major changes. A statistical analysis of the data is contained in **Doc S2, Table 8**.

**Table 3:** Relative nitrogen (N) and carbon (C) contents as well as C/N-ratios in wild-type (Col-0, Ws-2), single, and double mutant plants defective in the TPT and/or XPT.

Plant line	fw/dw-ratio	N (%)	C (%)	C/N-ratio
Ws-2	9.65 ± 0.46	6.49 ± 0.24	40.59 ± 0.29	6.30 ± 0.26
*xpt-1*	9.57 ± 0.37	6.77 ± 0.14	40.26 ± 0.29	5.95 ± 0.12
*tpt-1*	8.07 ± 0.12	5.50 ± 0.20	39.74 ± 0.17	7.26 ± 0.25
*tpt-1/xpt-1*	10.58 ± 0.66	5.95 ± 0.08	36.92 ± 0.34	6.21 ± 0.09
Col-0	9.39 ± 0.17	6.17 ± 0.22	39.91 ± 0.08	6.50 ± 0.23
*tpt-2*	9.02 ± 0.41	5.20 ± 0.18	39.82 ± 0.19	7.69 ± 0.25
*tpt-2/xpt-1*	8.64 ± 0.33	6.04 ± 0.05	38.93 ± 0.15	6.45 ± 0.08
*adg1-1/tpt-2*	11.00 ± 0.25	7.32 ± 0.06	36.60 ± 0,15	5.00 ± 0.05


*Plant were grown in soil under HL-conditions (PFD = 300 μmol⋅m^-*2*^⋅s^-*1*^) in the long-day. Samples were harvested in the middle of the light period. The data represent the mean ± SE (*n* = 5). A statistical analysis of the data is contained in **Doc S2, Table 8**.*

### Feeding of Suc, but Not of Gln Partially Rescued the Growth and Photosynthesis Phenotype of *tpt-1[2]/xpt-1* Double Mutants

Metabolome data, in particular the assessment of carbohydrate and amino acid contents as well as the C/N-ratios suggests that the growth phenotype of *tpt-2[1]/xpt-1* might be based on co-limitations of C- and N-metabolism. The abundance of both elements was only little affected in the double mutants despite of severe growth retardation, indicating that, on the whole plant level, both elements must be dramatically depleted.

We addressed this hypothesis of co-limitation by growing double mutant and wild-type plants on 1/2 MS agar plates supplemented with 50 mM Suc or 2 mM Gln (as carbon and/or nitrogen source), either individually or in combination. Feeding with higher amino acid concentrations was avoided because even wild-type plants showed impaired growth and lesions on the leaves. As GPT2 can be induced in leaves by exogenously fed sugars (see Schmitz et al., 2012, and references therein) we also deployed a *gpt2-1/tpt-2/xpt-1* triple mutant in this experiment, in order to exclude any effect based on increased GPT2 activity.

The growth behavior of the plants was assessed from rosette areas determined at four time points after sowing (**Supplementary Figure 2**) and the average growth rates of each line were compared in absolute and relative terms (**Supplementary Figure 3**). Furthermore at two time points of the feeding experiment photosynthesis parameters were determined by PAM fluorometry. The F_v_/F_m_-ratios and the induction kinetics of ETR are shown in **Figure 7**. Feeding with Suc, but not with Gln, partially rescued both the growth and photosynthesis phenotype of the *tpt-1[2]/xpt-1* double mutant and of *adg1-1/tpt-2* (**Figure 7**, **Supplementary Figure 3**). The rescue of *adg1-1/tpt-2* by feeding carbohydrates has already been demonstrated by Schmitz et al. (2012) and Heinrichs et al. (2012). Moreover the combined feeding with Suc and Gln resulted in a further significant increase in the growth rates of *adg1-1/tpt-2*, an effect that was also present as a trend in *tpt-1[2]/xpt-1* (**Supplementary Figure 3**). A statistical analysis of the feeding experiment is summarized in **Doc S2, Tables 9** (for comparisons between the lines) and **Doc S2, Tables 10** (for comparisons between treatments).

The analysis of the photosynthetic parameters revealed some more details. The visual impressions of the F_v_/F_m_-images shown in **Figure 7** (A-H) were supported and refined by a comparisons of Chl *a* fluorescence parameters (**Supplementary Figure 4**) and the induction of relative ETR (**Figures 7I–P**). After induction of photosynthesis with HL (i.e., at a PFD of 315 μmol⋅m^-2^⋅s^-1^) the depression of relative ETR in *tpt-1[2]/xpt-1* and *adg1-1/tpt-2* double mutants (**Figures 7I,J**) recovered markedly when Suc was fed to the plants (**Figures 7K,L** and **Supplementary Figure 4**). This recovery was, however, weakened when Gln was fed in combination with Suc (**Figures 7O,P** and **Supplementary Figure 4**). Similarly, relative ETR was also slightly diminished in both wild-type accessions under the same conditions. Feeding of Gln alone also resulted in a small enhancement of relative ETR in *tpt-2/xpt-1* and *adg1-1/tpt-2*, but not in the *gpt2-1/tpt-2/xpt-1* triple mutant (**Figures 7M,N**). Like HCF in the dark-adapted state, relative ETR was less responsive to Suc and Gln feeding in the triple mutant (**Supplementary Figure 4**). Strikingly feeding of Gln resulted in a delay of relative ETR induction in both wild-type plants, which was, however, less marked in the double and triple mutants, in particular when Gln was fed in combination with Suc (**Figures 7M–P**). A statistical analysis of the data is contained in **Doc S2, Table 11** (for comparisons between the lines) and **Doc S2, Table 12** (for comparisons between treatments).

### Components of the Photosynthetic Electron Transport Chain Are Less Abundant in Thylakoids From *tpt-1[2]/xpt-1* Double Mutants

**FIGURE 8:**
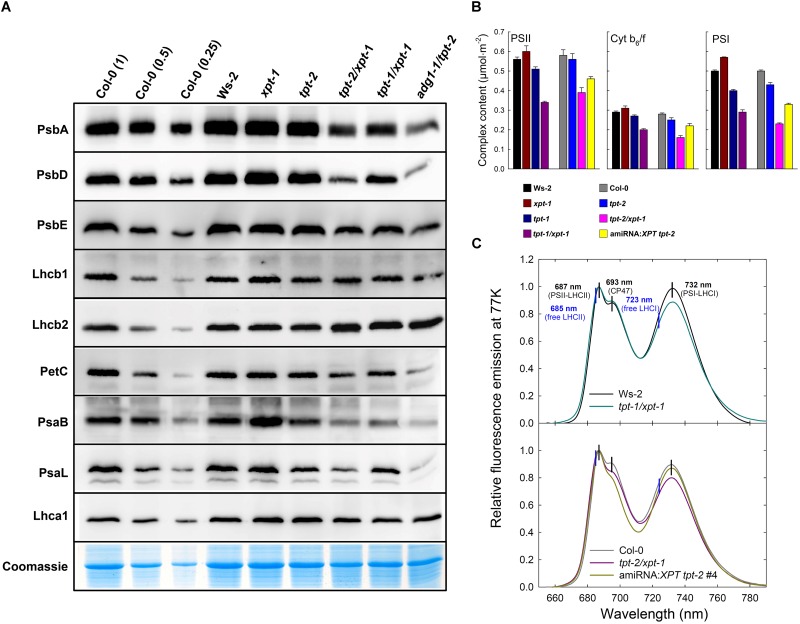
Immunoblots of thylakoid proteins, spectroscopic determinations of functional PS components, and fluorescence emission spectra of Chl *a* at 77 K. **(A)** Immunoblots of thylakoid proteins associated with photosynthesis after separation of total proteins isolated from leaves of HL-grown Col-0 and Ws-2 wild-type plants, the *xpt-1* and *tpt-2* single mutants as well as the *tpt-1/xpt-1*, *tpt-2/xpt-1*, and *adg1-1/tpt-2* double mutants on SDS–PAGE. All gels for the blots were loaded on an equal total leaf protein basis (approximately 10 μg per lane) and a dilution series of Col-0 was used for semi-quantitative analyses. **(B)** Spectroscopic determinations of functional components of PSII, PSI and the cyt b_6_/f complex. The data represent the mean ± SE of *n* = 3 experiments. A statistical analysis of the data is contained in **Doc S2, Table 13**. **(C)** Relative fluorescence emission spectra of Chl in isolated thylakoids from Ws-2 and Col-0 wild-type plants as well as *tpt-1/xpt-1* and *tpt-2/xpt-1* double mutants and the amiRNA:*XPT tpt-2* #4 line determined at 77K. The spectra were normalized for the peak intensity at 687 nm. The vertical black lines indicate absorption maxima of PSII and PSI components, whereas the blue vertical lines point at expected absorption maxima of free LHCII or LHCI not attached to the core components of both photosystems.

In a previous report we could show that the HCF-phenotype in *adg1-1/tpt-2* was accompanied by changes in the abundance of thylakoid proteins involved in the photosynthetic light reaction (Schmitz et al., 2012). A similar response was observed here for the *tpt-1[2]/xpt-1* double mutants grown under HL-conditions albeit less marked as compared to *adg1-1/tpt-2* (**Figure 8A**). The abundance of the PSII core components PsbA (D1), PsbD (D2) and the α-subunit of Cyt b_559_ (PsbE) was diminished by approximately 50% in the *tpt-2/xpt-1* double mutant compared to about 80-90% in *adg1-1/tpt-2*. Again, in *adg1-1/tpt-2*, PsbE accumulation seemed to be far less diminished than that of the other PSII reaction center subunits (compare Schmitz et al., 2012). Cyt b_559_ can accumulate independently of the other PSII subunits, for example in etioplasts, and might function as a plastoquinol oxidase in the dark (Bondarava et al., 2010). The abundance of the plastome-encoded PSI core component, PsaB, was even diminished by about 70% in the *tpt-1[2]/xpt-1* double mutants. Interestingly, the nuclear-encoded PSI inner antennae protein, PsaL, showed similar changes as PsaB. In contrast, contents of nuclear-encoded LHC2 proteins of PSII (Lhcb1 and Lhcb2) remained nearly unchanged both in the *tpt-1[2]/xpt-1* and *adg1-1/tpt-2* double mutants, whereas Lhca1 of PSI was slightly diminished in *tpt-1[2]/xpt-1*. PetC (the essential nuclear-encoded Rieske protein), which was used as diagnostic subunit of the Cyt b_6_/f complex was also diminished in its abundance. The composition of thylakoid membrane proteins in the *tpt-1[2]/xpt-1* double mutants appeared to be altered in the same direction as in *adg1-1/tpt-2* albeit with a much less marked amplitude.

**FIGURE 9:**
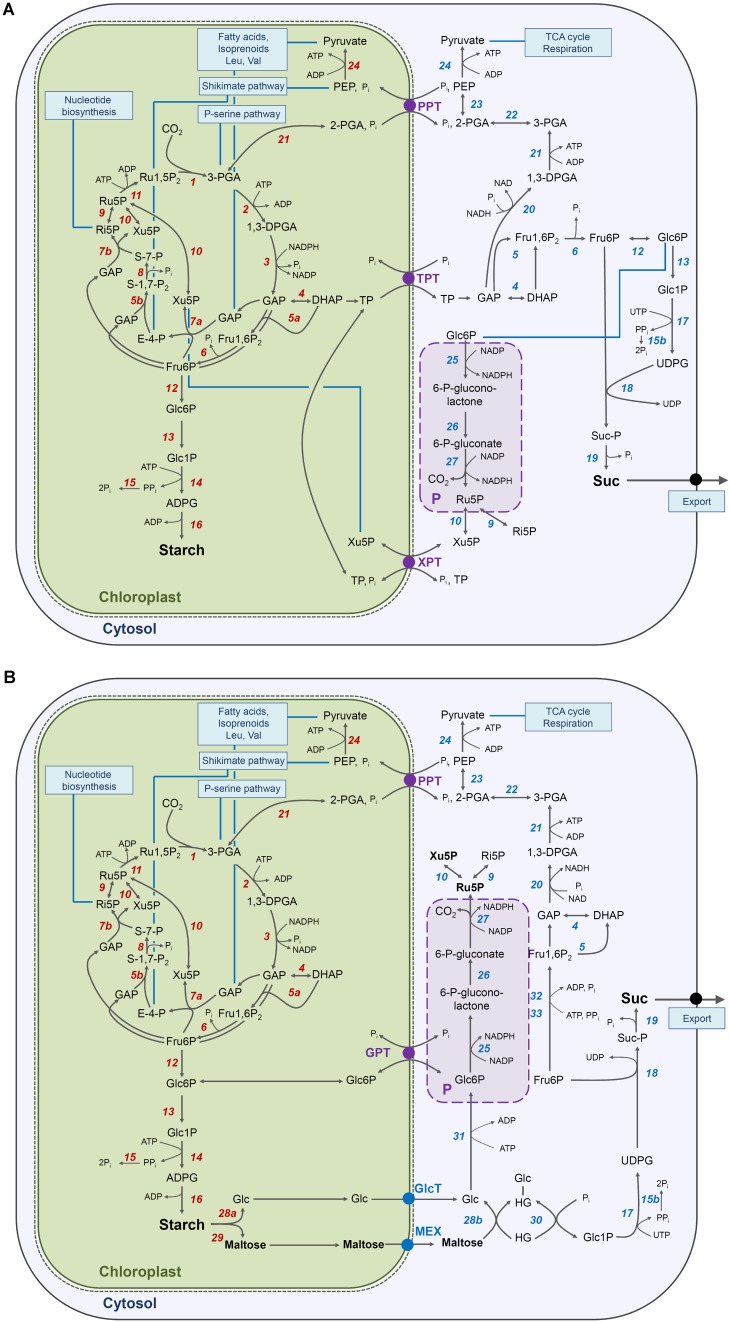
Metabolic sketches of carbohydrate metabolism in a mesophyll cell of wild-tpye and mutant plants defective in the TPT and XPT during illumination. In **(A)** the major path of carbon from CO_2_ assimilation in the Calvin-Benson cycle to starch in the stroma and Suc in the cytosol as well as glycolysis are illustrated for wild-type plants, whereas in **(B)** the diversion of carbon flow via starch turnover (i.e., the ‘night path of cabon’) and the utilization of Glc and maltose for Suc biosynthesis is shown for *tpt-1[2]/xpt-1*. Furthermore the extraplastidial branch of the OPPP, which might be localized in the cytosol or peroxisomes (P) leads to an accumulation of pentose phosphates only in the double mutant. A GPT has been included in **(B)** in order to demonstrate that, in principle, an exchange of Glc6P across the envelope would be possible. The numbers in italics denote the enzymes: ***1*** ribulose-1,5-bisphosphate carboxylase/oxygenase (RubisCO), ***2*** phosphoglycerate kinase (PGK), ***3*** NADP glyceraldehyde-3-phosphate dehydrogenase (NADP-GAPDH), ***4*** triosephosphate isomerase (TIM), ***5a,b*** (trans)aldolase, ***6*** fructose-1,6-bisphosphatase (FbPase), ***7a,b*** transketolase, ***8*** sedoheptulose-1,7-bisphosphatase (SbPase), ***9*** ribose-5-phosphate isomerase, ***10*** ribulose-5-phosphate epimerase, ***11*** phosphoribulokinase (PRK), ***12*** phosphoglucose isomerase (GPI), ***13*** phosphoglucomutase (PGM), ***14*** ADPglucose pyrophosphorylase (ADG), ***15*** inorganic pyrophosphatase (PP_i_ase), ***15b*** PP_i_ase and/or PP_i_-dependent tonoplast proton pump and/or PFP (see ***33***), ***16*** starch synthases (both soluble and glranule-bound), ***17*** UDPglucose pyrophosphorylase (UDG), ***18*** sucrose phosphate synthase (SPS), ***19*** sucrose phosphate phosphatase (SPP), ***20*** NAD-GAPDH, ***21*** glycolytic PGK, ***22*** phosphoglycero mutase (PGyM), ***23*** enolase, ***24*** pyruvate kinase, **25** glucose 6-phosphate dehydrogenase (G6PDH), **26** 6-phosphoglucono-lactonase, **27** 6-phosphogluconate dehydrogenase, ***28*** isoamylase and disproportionating enzyme1 (DPE1) (***a***) or 2 (DPE2) (***b***), ***29*** β-amylase, ***30*** cytosolic glucane phosphorylase (PSH2). ***31*** hexokinase (HK), ***32*** phosphofructokinase (PFK), ***33***, pyrophosphate-dependent phosphofructokinase (PFP; see 15b).

The immunological data were supported by spectroscopic quantifications of PSII, PSI and the Cyt b_6_/f complex (**Figure 9B**). On a leaf area basis, all three complexes were significantly diminished in plants with a combined defect in TPT and XPT. The most pronounced decrease was observed in *tpt-2/xpt-1* followed by *tpt-1/xpt-1* (leaky for TPT expression), and one of the amiRNA:*XPT tpt-2* lines (leaky for XPT expression). However, compared to *adg1-1/tpt-2* (Schmitz et al., 2012) the decrease in thylakoid complexes was less marked in plants with a combined lesion in the TPT and XPT.

As total Chl contents were substantially decreased in the *tpt-1[2]/xpt-1* double mutants (**Table 1**), the composition of PS complexes showed a different pattern when expressed on a Chl basis (**Supplementary Figure 5**) compared to the leaf area basis (**Figure 8B**). Instead of the common decrease in the contents of all three complexes in the *tpt-1[2]/xpt-1* double mutants, a more diverse picture emerged showing the most pronounced differences for the *tpt-2/xpt-1* double mutant allele. The content of PSII was slightly increased compared to Col-0, whereas the PSI content was significantly decreased resulting in a drop of the PSI/PSII-ratio from 0.86 in Col-0 to 0.59 in *tpt-2/xpt-1*. The content of the Cytb_6_/f complex remained unchanged compared to both wild types. Moreover, there seemed to be a direct correlation between PSI contents and Chl *a/b*-ratios, which was also lower in *tpt-2/xpt-1*. A statistical analysis of the data is contained in **Doc S2, Table 13**.

The energetic coupling between LHCs and the photosystems can be estimated from determinations of the Chl *a* fluorescence emission spectra at 77K. The *adg1-1/tpt-2* double mutant exhibited characteristic shifts in the emission spectra and thus revealed that a high portion of LHCs were detached from the PSII and PSI core components leading to a substantial increase in F_o_ (Schmitz et al., 2012). For the *tpt-1[2]/xpt-1*double mutants and the amiRNA:*XPT tpt-2* line similar shifts, i.e., from 687 to 685 nm in PSII or from 732 nm to 723 nm in PSI, were missing (**Figure 8C**), indicating an intact energetic coupling between LHCs and reaction centers. Differences in the relative peak heights of PSII and PSI between wild-type and the double mutant plants underline the observed changes in PSII and PSI abundance (**Figures 8A,B**).

## Discussion

The present study reveals two major unexpected findings, (1) the lack of any consistent visible or biochemical phenotype of *xpt* single mutants (see **Doc S1**), and (2) the dramatic growth retardation, impaired photosynthesis, and perturbed primary metabolism in double mutants with combined lesions in the TPT and XPT. The second finding will be particularly discussed with reference to central carbon metabolism and its regulation in the leaf mesophyll, as well as its feedback on amino acid metabolism and photosynthesis.

### The Complete Diversion of Photosynthetic Carbon Flow to Sucrose Production via Starch Turnover Leads to a Massive Maltose Accumulation

When the ‘day path of carbon’ is restricted the ‘night path’ can take over and is also active in the light, as has already been shown for plants with a defect in the TPT (Häusler et al., 1998; Schneider et al., 2002; Walters et al., 2004; Schmitz et al., 2012, 2014), the cytosolic phosphoglucose isomerase (PGI, Kunz et al., 2014), and cytosolic fructosebisphosphatase (FbPase; Rojas-González et al., 2015). Remarkably, for the *tpt-1[2]* single mutants the bypass via starch turnover had only little consequences on growth and photosynthesis. Only if starch synthesis was inhibited in combination with TP export in the *adg1-1/tpt-1[2]* double mutants, a harsh growth and photosynthesis phenotype occurred (Schneider et al., 2002; Schmitz et al., 2012), which was interpreted as a consequence of carbohydrate deficiencies and an imbalance in retrograde signaling (Heinrichs et al., 2012; Schmitz et al., 2014; Häusler et al., 2014a). Interestingly, in rice, which forms only low amounts of transitory starch, a sole deficiency in the TPT already leads to marked growth retardation (Lee et al., 2014).

In the *tpt-1[2]/xpt-1* double mutants the carbon flow in the light is most likely entirely diverted via starch turnover (**Figure 9B**). However, in contrast to *adg1-1/tpt-2*, the *tpt-1[2]/xpt-1* double mutants contain substantial amounts of carbohydrates, both during the light and dark period. Hence, diminished carbohydrate levels as major cause for growth retardation in *tpt-1[2]/xpt-1* appeared less likely. Still, as shown for *adg1-1/tpt-2* (Schmitz et al., 2012), feeding with Suc could at least partially rescue the growth and photosynthesis phenotype of the *tpt-1[2]/xpt-1* double mutants, an observation that seemed to be contradictory at the first glance. However, considering the following two assumptions, i.e. (1) energy costs and (2) maltose accumulation, the beneficial effects of Suc feeding to *tpt-1[2]/xpt-1* might become explainable. (1) When the ‘day’- and ‘night path of carbon’ are compared, the energy costs per molecule Suc synthesized increase from eight to at least 11 ATP equivalents (**Figures 9A,B**). In the cytosol the additional ATP requirement might be covered by glycolysis; and/or respiration. However, ATP availability seemed unaffected in the light and dark, ruling out a restriction on energy supply (compare **Figure 5**). (2) As maltose in leaves derives entirely from starch breakdown, the ‘night path of carbon’ was underlined by steeply increased maltose levels in *tpt-2* and even more so in *tpt-2/xpt-1* in the dark and light. Interestingly, constraints in maltose transport (i.e., *mex-1* Niittylä et al., 2004) or its cytosolic turnover (Lu and Sharkey, 2004), resulted in dwarfish and chlorotic phenotypes of the respective mutants. In the *mex1* mutant maltose contents were increased 40-fold relative to the wild type, which compares nicely to the 35-fold increase in maltose levels in *tpt-2/xpt-1* at the beginning of the light period, in particular as this double mutant contains the strong knockout allele of the TPT. Maltose accumulation in *tpt-1[2]/xpt-1* might hence be directly linked to the observed growth retardation of the double mutant. Increased maltose levels in *tpt-1[2]/xpt-1* reflect high rates of starch degradation probably combined with limitations on export and/or maltose metabolism in the cytosol via DPE2 and PSH2 (**Figure 9B**). Again these constraints are probably relieved by feeding with Suc.

### Pentose Phosphate Accumulation in *tpt-1[2]/xpt-1* Point at an Impaired Re-Import of Extraplastidial OPPP End Products

One of the most prominent effects on metabolite levels was the massive accumulation of the Calvin-Benson cycle and OPPP intermediates Xu5P and Ru5P in the *tpt-1[2]/xpt-1* lines (**Figure 5**). As the pentose phosphates accumulated more prominently in the light than in the dark, the chloroplast stroma might by assumed as possible site of accumulation. Yet, according to critical evaluations of mathematical models aiming at describing kinetic properties of the Calvin-Benson cycle (Jablonsky et al., 2011), such an accumulation would lead to an ultimate and rapid breakdown of the cycle and hence the inhibition of photosynthesis. Moreover other Calvin-Benson cycle intermediates like 3-PGA, DHAP, Fru6P, or S-7-P would also be massively affected. However, they remained relatively unchanged in *tpt-1[2]/xpt-1* compared to wild-type and single mutant plants (**Figure 5**). Furthermore the equilibrium of the phosphoribulokinase (PRK) reaction is far on the side of the products ADP and Ru1,5P_2_ and would hence drain the pools of monophosphates dramatically unless PRK was inhibited (e.g., Lescovac et al., 2008). Hence, an extraplastidial accumulation of pentose phosphates deriving from the OPPP is more likely.

The extraplastidial OPPP, which is localized in the cytosol and, under certain conditions, also in peroxisomes (Meyer et al., 2011; Hölscher et al., 2014; Hölscher et al., 2016), is restricted to the oxidative steps and lacks the regenerative part of its plastidial counterpart (Kruger and von Schaewen, 2003). Ru5P as product of the oxidative decarboxylation of 6-P-gluconate, is in equilibrium with the other pentose phosphates via specific epimerases and isomerases (**Figure 9**). However, a further metabolism via transaldolase or transketolase is not possible, as these enzymes are found exclusively in plastids (e.g., Aramemnon data base, Schwacke et al., 2003). Thus, the massive accumulation of pentose phosphates observed in *tpt-1[2]/xpt-1* supports the view that, in the wild-type, the XPT retrieves excessive pentose phosphates from the extraplastidial space and makes it available for further metabolism in the plastid. However, it is intriguing that pentose phosphates appeared to accumulate also in *tpt-2* (albeit less marked compared to *tpt-1[2]/xpt-1*) rather than in *xpt-1* in the light (**Figure 5**). It is tempting to speculate that day-time starch mobilization, which even occurs in wild-type plants under long-day conditions (Fernandez et al., 2017), generally promotes the cytosolic branch of the OPPP. For potato plants there are indications that externally fed glucose activates specifically the cytosolic Glc6PDH (Hauschild and von Schaewen, 2003). However, it remains to be shown whether increased Glc levels deriving directly from starch mobilization or indirectly from maltose metabolism in *tpt-1[2]* or *tpt-1[2]/xpt-1* trigger a similar activation, in particular as leaf contents of Glc in these lines were not significantly different compared to the wild type for most of the day and night (compare **Doc2, Table 4C**).

### The Complete Block in TP Export From Chloroplasts Affects Levels of Tricarbonic Acid Cycle Intermediates and Amino Acids as Well as C/N-Ratios

The *de novo* biosynthesis of amino acids relies on the provision of the TCA cycle intermediate 2-OG (e.g., Lam et al., 1996; Pratelli and Pilot, 2014). In leaves pyruvate, required as substrate for the TCA cycle, derives from glycolysis starting from TPs exported from the chloroplasts in the light. It is hence conceivable that an impaired TP dependent glycolysis restricts amino acid biosynthesis, unless a sufficient glycolytic flux can be maintained starting from Fru6P via ATP- or PPi-dependent phosphofructokinase (PFK or PFP; **Figure 9B**).

Of the TCA intermediates fumarate contents dropped substantially in the *tpt-2/xpt-1* double mutant allele compared to wild-type or single mutant plants, whereas succinate exhibited a slight relative increase towards the middle of the light period. Chia et al. (2000) proposed that fumarate, besides of its roles in the TCA cycle, might act as major storage form of assimilated carbon in several plant species including *A. thaliana*. There are further indications that fumarate is required for rapid nitrogen assimilation, when plants are grown on high nitrogen (Pracharoenwattana et al., 2010). However, a comparison of steady state levels of organic acid or other metabolic intermediates are not sufficient to interpret changes in metabolic fluxes. In this contexts it ought to be considered that catabolism of amino acids also leads to the release of organic acid, amongst them 2-OG, fumarate, or succinate (Hildebrandt et al., 2015).

Apart from *tpt-2/xpt-1*, total amino acid levels in the middle of the dark period were lower in lines affected in the TPT alone or in combination with the XPT, but recovered towards the middle of the light period. These changes will be discussed together with altered C/N-ratios (see below). Of the individual amino acids, Gly contents were decreased in both *tpt-1[2]/xpt-1* double mutants in the light and dark. Interestingly, in *tpt-1[2]* single mutants Gly levels were also diminished, however, most prominently in the middle of the dark period. Gly can be formed in the photorespiratory cycle (Dellero et al., 2016) and is involved in Ser formation via Ser-hydroxymethyl-transferase (SHM). Thereby, Gly plays also a role in C1-metabolism (Christensen and MacKenzie, 2006). However, if photorespiration were involved in the depletion of Gly, changes in its content ought to be more prominent in the light- rather than the dark period. A support for the contribution of photorespiration in the depletion of Gly came from the direct correlation of Gly levels with the relative ETR determined at a PFD similar to the growth light of the plants. However, Ser which can derive from Gly as a direct precursor remained unaffected by ETR. In addition, Gly might also directly derive from Ser produced by the plastidial phosphoserine pathway (Ros et al., 2014). For this pathway of Ser biosynthesis, the substrate 3-PGA presumably needs to be imported into chloroplasts by the TPT in the dark, and Gly might be generated from Ser by a plastidial SHM isoenzyme (SHM3) or probably by mitochondrial SHM2 (Engel et al., 2011). However, in the absence of the TPT, the night-time import of 3-PGA into chloroplasts would be blocked. In other metabolic pathways Gly is, for instance, required for *de novo* purine biosynthesis in plastids and mitochondria at the step from phosphoribosylamine to glycineamide ribonucleotide (Zrenner et al., 2006). Gly is also a constituent of glutathione and thereby involved in oxidative stress responses (Hasanuzzaman et al., 2017). At this stage it is not clear, whether the depletion of Gly observed in *tpt-1[2]/xpt-1* double mutants is governed by its consumption and/or its synthesis. Future flux determinations will help to clarify this point. The relative increase in minor amino acids, in particular those deriving from imported PEP or from pyruvate (i.e., the aromatic amino acids as well as Val and Leu), suggests that glycolytic PEP production seems not to be impaired severely in *tpt-1[2]/xpt-1* (Compare **Figures 9A,B**).

Moreover, the assessment of C/N-ratios provided valuable information on constraints in primary metabolism. In both *tpt-1[2]* single mutants C/N-ratios were significantly higher based on a drop in N compared to the respective wild-type lines, suggesting that a disturbed carbohydrate metabolism feeds back on N-assimilation and distribution. This notion is supported by a severe decrease in free amino acids in the middle of the dark period in *tpt-1[2]* single mutants. Interestingly, C/N-ratios in the *tpt-1[2]/xpt-1* double mutants recovered due to an increase in N rather than a decrease in C. It is tempting to speculate that the recovery in C/N-ratios is a result of growth retardation in the *tpt-1[2]/xpt-1* double mutants, probably to compensate for an overall loss in N. Remarkably contents of free proteinogenic amino acids remained either unchanged or were decreased in the *tpt-1[2]/xpt-1* double mutants on a dry weight basis. Hence a restriction in N-metabolism might substantially contribute to the growth inhibition of the double mutants. However, in contrast to *adg1-1/tpt-2* feeding of sucrose in combination with Gln to the *tpt-1[2]/xpt-1* double mutants could not significantly improve growth as compared to feeding with Suc alone (compare **Supplementary Figure 3** and **Doc S2, Table 10**).

### A Block in TP Export From Chloroplasts Feeds Back on Photosynthesis

The *tpt-1[2]/xpt-1* double mutant showed similar constraints in photosynthesis as *adg1-1/tpt-1[2]* (Schmitz et al., 2012), albeit much less pronounced. The mechanistic basis behind the HCF-phenotype in *adg1-1/tpt-1[2]* has been studied to some extent by Häusler et al. (2009) and Schmitz et al. (2012) and will not be discussed in depth here. Similar to *adg1-1/tpt-2* immunoblots indicate a decreased abundance of PSII and PSI core proteins in *tpt-1[2]/xpt-1*, which is again much less pronounced compared to *adg1-1/tpt-2* (Schmitz et al., 2012). In contrast to the core components of both photosystems, the abundance of antennae proteins remained unaffected and resulted in the accumulation of free, uncoupled LHC proteins of both photosystems, as evidenced by characteristic shifts in the 77K Chl *a* fluorescence emission spectra (Schmitz et al., 2012). However, these characteristic shifts in fluorescence emission spectra at 77K are missing in *tpt-1[2]/xpt-1*, which is in line with the less severe reductions in the abundance of the reaction centers. Hence excitonic coupling between LHCs and reaction centers is intact in *tpt-1[2]/xpt-1*. The decreased abundance in core components of both photosystems and especially of the cytochrome b_6_f complex, which is usually rate-limiting for photosynthetic electron transport (reviewed by Anderson, 1992; Schöttler and Tóth, 2014), is reflected in diminished relative ETR compared to wild-type or single mutant plants.

The molecular basis of the diminished photosynthetic complex accumulation is still not completely understood and has been interpreted recently for the *adg1-1/tpt-2* double mutant as impaired or re-directed retrograde signaling that co-ordinates the expression of nuclear- and plastome-encoded photosynthesis genes (Häusler et al., 2014a, and references therein). Hence perturbations in central carbon metabolism might impair retrograde or other signaling mechanisms, that are involved for instance in acclimation responses (e.g., Schöttler and Tóth, 2014; Schöttler et al., 2015).

Moreover, the mild HCF-phenotype in plants with impaired TPT and XPT could not be resolved in this initial study and more refined methods are required involving studies on the capacity of electron acceptors or on the abundance of additional components like NAD(P)H dehydogenase complexes.

## Conclusion and Outlook

Based on the first part of this study the functional role of the XPT in metabolism and development of *A. thaliana* is still obscure. Although the XPT is ubiquitously expressed in *A. thaliana* the single mutant lacks any major phenotype under the growth conditions applied here. In contrast to *tpt-2*, in *xpt-1* single mutant not even pentose phosphates accumulate, as one of the major transport substrates of the XPT. It remains to be shown, whether harsher environmental conditions, or abiotic/biotic stresses trigger any different responses in *xpt* single mutants compared to the wild type. In particular conditions ought to be tested that require high rates of NADPH production by the extraplastidial branches of the OPPP such as the reactive oxygen species (ROS) burst after pathogen attacks (e.g., Scharte et al., 2009, and references therein). Diminished NADPH generation in the peroxisomes might also restrict the formation of nitric oxide and jasmonic acid as signals (Hölscher et al., 2016), which again play a major role in plant defense responses. Moreover, studies with the *tpt-1[2]/xpt-1* double mutant clearly showed that a major function of the XPT seems to be the retrieval of extraplastidial pentose phosphates in wild type plants. Hence we have verified this proposed function. However, more refined experiments involving ^13^C- and ^15^N-based flux analyses are required to determine perturbations in metabolism in more detail. Furthermore, the complete block of TP export and the diversion of carbon flow via starch have a number of additional implications and pose open questions that have not yet been experimentally addressed. For instance, can substantial quantities of Fru1,6P_2_ be formed in the cytosol when TP export from the chloroplasts is completely blocked? Is cytosolic FbPase as key regulatory step in Suc biosynthesis bypassed? How would the depletion in cytosolic TPs feed back on the level of the cytosolic regulator metabolite Fru2,6P_2_ (Stitt, 1990) and its inhibition of cytosolic FbPase or activation of PFP. Is a substantial glycolytic flux in the direction of pyruvate formation via PFP or PFK starting from Fru6P instead from C3 compounds possible in the light (**Figure 9B**)? Finally various aspects of modified signaling pathways ought to be addressed in the future.

## Author Contributions

EH conducted most experiments in the lab. MS analyzed Chl *a* fluorescence emission spectra at 77 K and determined contents of thylakoid complexes involved in photosynthetic eletron transport. Metabolome and C/N-elemental analyses were conducted in the lab of TM-A. SK was involved in the analysis of amino acid spectra. PD analyzed fatty acid contents and composition of seeds. ME initially characterized the XPT and isolated the *xpt-1* mutant allele. U-IF provided helpful advice and co-supervised the project. RH was the supervisor of the project and wrote the manuscript.

## Conflict of Interest Statement

The authors declare that the research was conducted in the absence of any commercial or financial relationships that could be construed as a potential conflict of interest.

## Footnotes

1 https://imagej.nih.gov/ij/

2 http://weigelworld.org/

3 http://www.r-project.org

4 http://bar.utoronto.ca/efp/cgi-bin/efpWeb.cgi

5 https://www.genome.jp/kegg-bin/show_pathway?map01230

## Supplementary Material

DATA SHEET S1 Contains 5 Supplementary Figures.

DATE SHEET S2 Contains 6 Supplementary Tables.

DATA SHEET S3 Contains Supplementary Document 1, which describes the phenotypic analysis of *xpt-1[2]* single mutants and amiRNA:*XPT* lines in the Ws-2 or Col-0 background. It comprises 4 Figures and 5 Tables including 1 Table with a statistical analysis.

DATE SHEET S4 Contains Supplementary Document 2 which comprises 13 Tables with statistical analyses of the data.
