# Supplementary material for: The Combined Loss of Triose Phosphate and Xylulose 5-Phosphate/Phosphate Translocators Leads to Severe Growth Retardation and Impaired Photosynthesis in Arabidopsis thaliana tpt/xpt Double Mutants
Source: Front Plant Sci. 2018 Oct 2;9:1331. doi: 10.3389/fpls.2018.01331 (PMC6175978; doi:10.3389/fpls.2018.01331)
Supplement: DATA SHEET S1 — Contains 5 Supplementary Figures. [file Data_Sheet_1.pdf]

Supplementary Figures

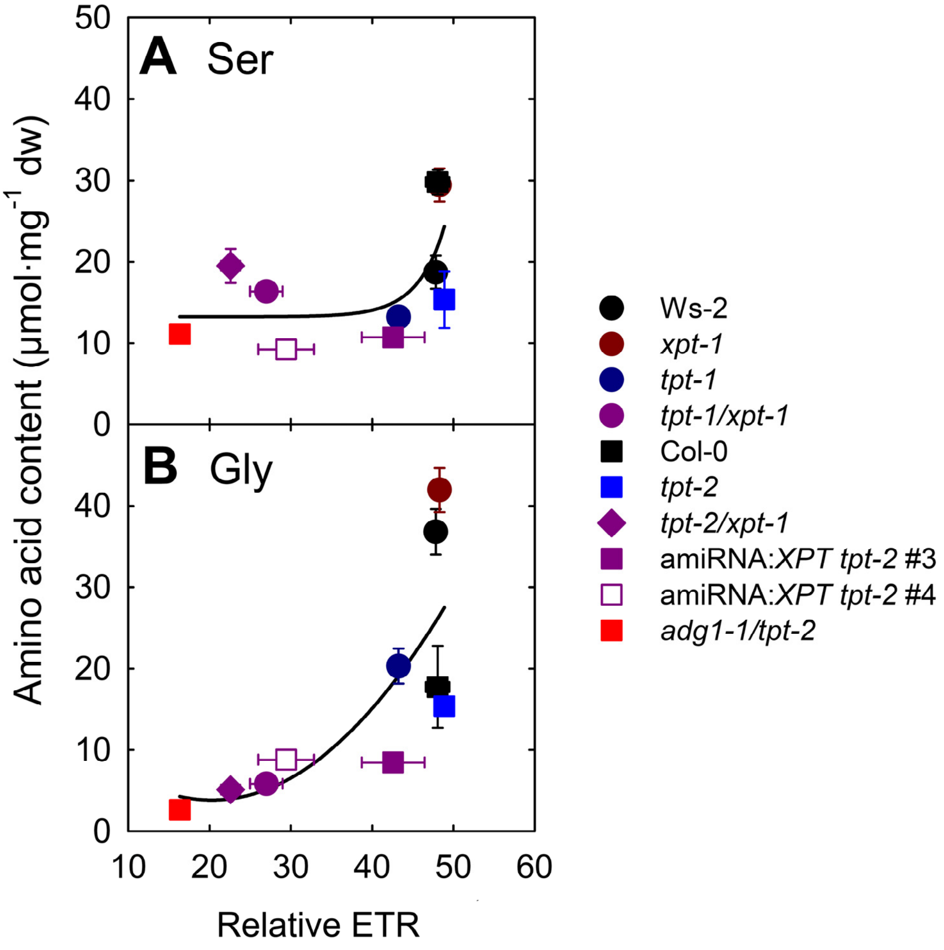

Supplementary Figure 1. Ser and Gly contents as a function of relative ETR determined for wild-type and mutant plants.

The absolute contents of Ser and Gly in wild-type and mutant plants impaired in XPT and/or TPT derived from Supplementary Table 6B were plotted against the average relative ETR determined under the same conditions for the same set of lines (see Figure 3B, main article). Plants were grown for three weeks under HL-conditions (PFD = 300 µmol·m^{-2}·s^{-1}) in the long-day. The curves were fitted to the data points by second polynomial regression.

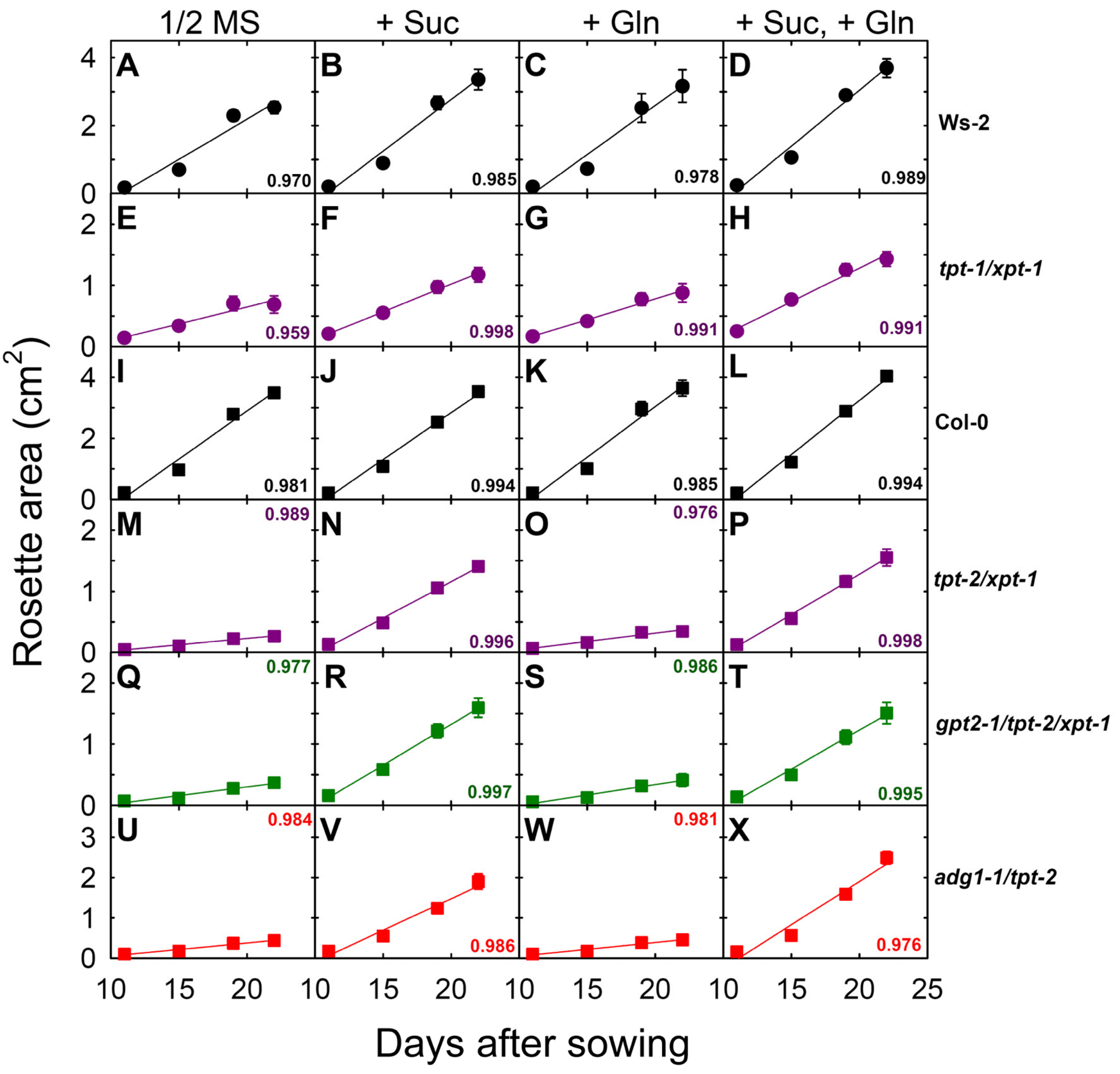

Supplementary Figure 2. Rosette growth rates of wild-type and mutant plants grown on agar plates supplemented with Suc and/or Gln.

Plants were grown under HL-conditions (PFD = 300 µmol·m^{-2}·s^{-1}) in the long-day on ½ MS agar plates with or without the supplement of 50 mM Suc and/or 2 mM Gln added either individually or in combination. The data are the mean ± SE of n = 5 replicates. The numbers in the subfigures indicate r^2-values supporting an almost linear correlation of the slopes. A statistical analysis of the data is contained in Doc S2, Tables 9 and 10.

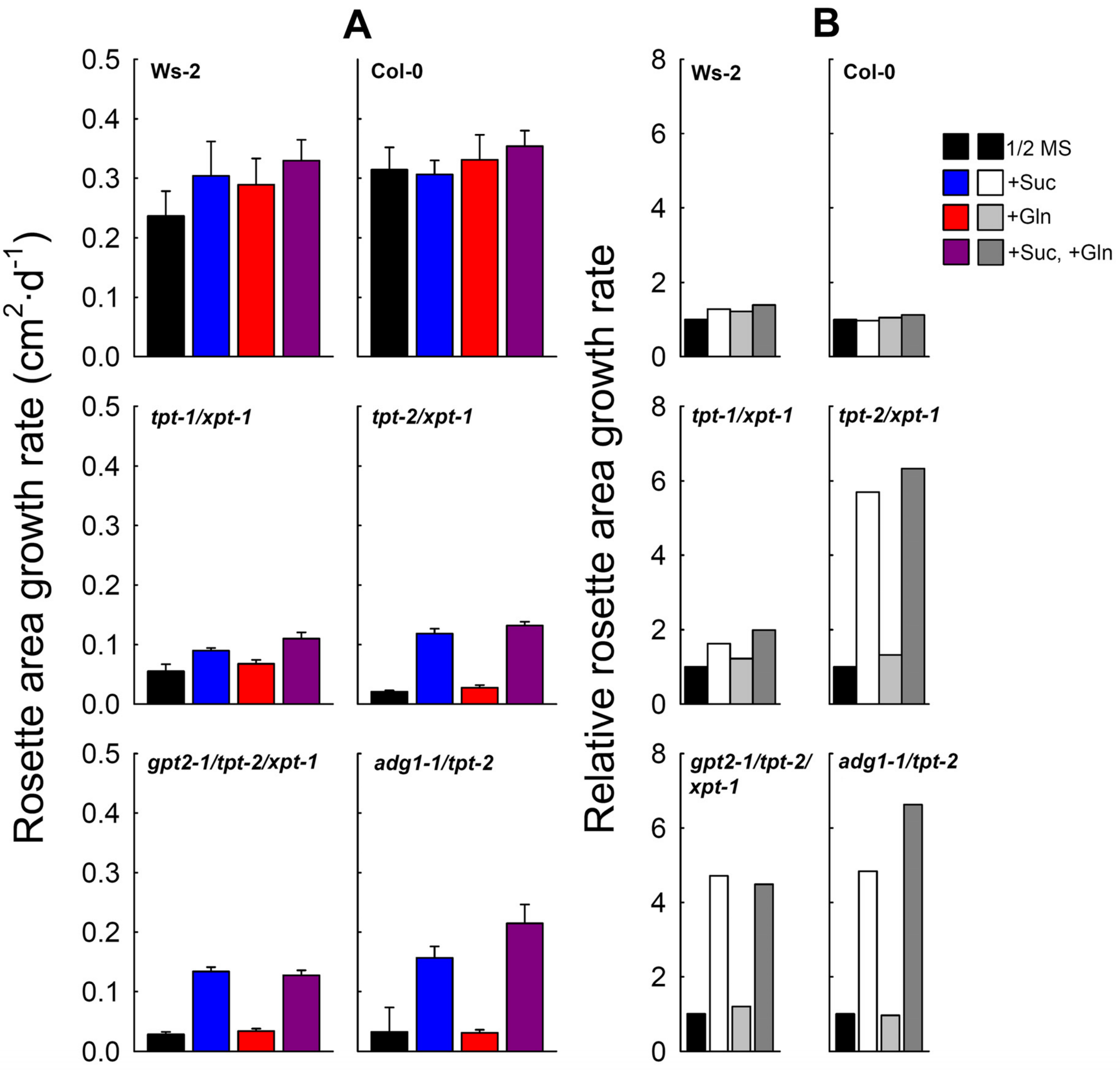

Supplementary Figure 3. Growth rates of rosette areas in the presence or absence of externally fed substrates.
Absolute (**A**) or relative (**B**) growth rates were calculated from the linear functions of rosette area increase *vs* time of growth between days 10 and 25 after sowing (Supplementary Figure 2). Plants were grown under HL-conditions (PFD = 300 $\mu mol \cdot m^{-2} \cdot s^{-1}$) in the long-day on 1/2MS agar either in the absence and presence of 50 mM Su, 2 mM Gln, added either individually or in combination. The data represent the mean ± SE of n = 5 replicates. A statistical analysis is contained in Doc S2, Tables 9 and 10.

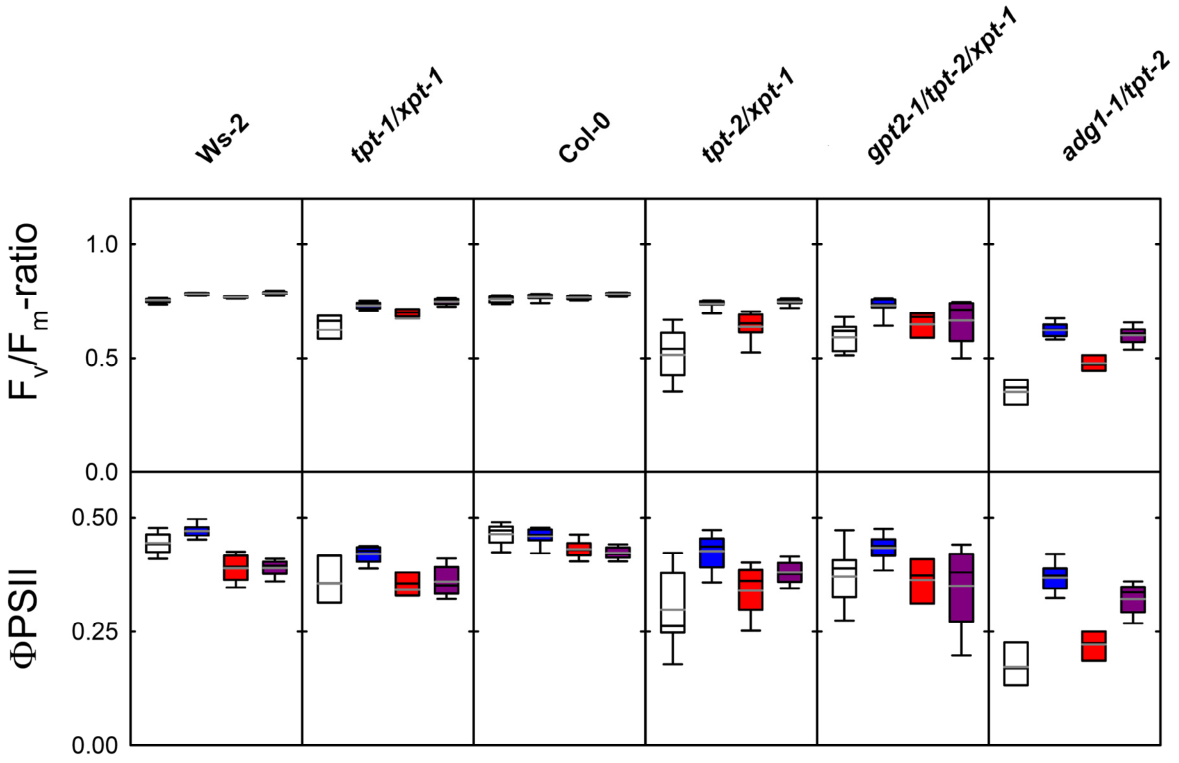

Supplementary Figure 4. Photosynthesis parameters of wild-type and mutant plants grown on agar plates supplemented with Suc and/or Gln.

Plants were grown under HL-conditions (PFD = 300 µmol·m^{-2}·s^{-1}) in the long-day on ½MS agar plates in the absence or presence of 50 mM Suc and/or 2 mM Gln added either individually or in combination. The plants were dark-adapted for 30 min and F_v/F_m-ratios determined. After induction of photosynthesis with a PFD of 316 µmol·m^{-2}·s^{-1}, ΦPSII was determined at steady state. The box colour indicate, white = ½MS control, blue = 50 mM Suc, red = 2 mM Gln, dark purple = 50 mM Suc and 2 mM Gln. The data represent the mean ± SE of n = 9 to 18 replicates. A statistical analysis of the data is contained in Doc S2 11 and 12.

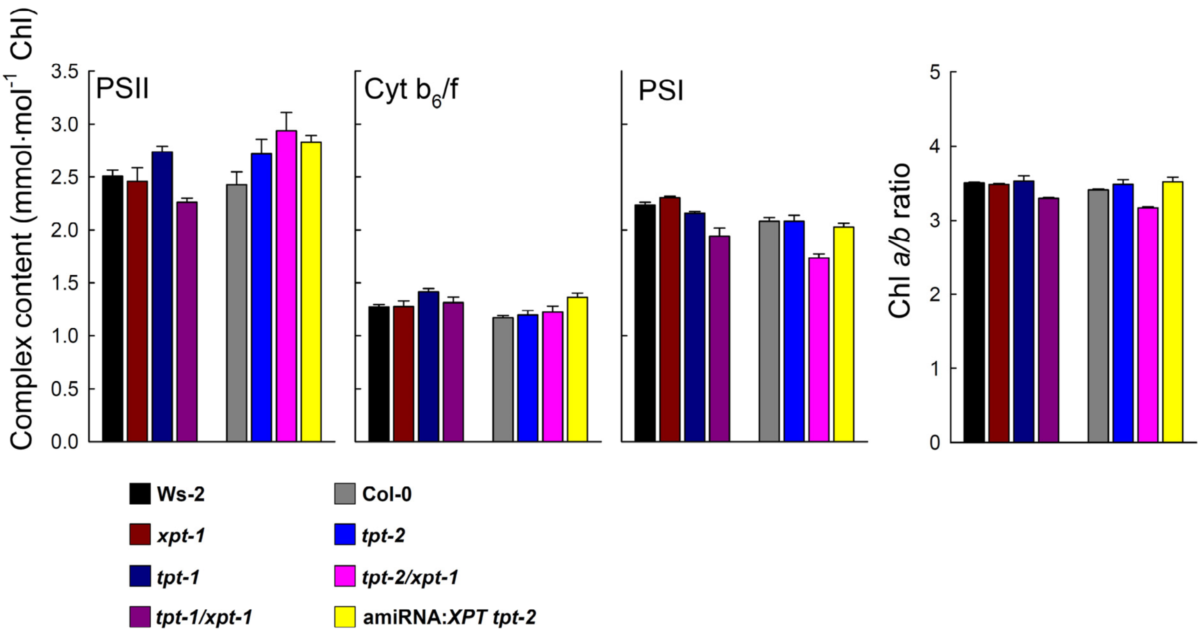

Supplementary Figure 5. Spectroscopic analysis of thylakoid membranes isolated from wild-type and mutants plants.

Plants were grown in soil under HL-conditions (PFD = 300 µmol·m^{-2}·s^{-1}) in the long-day. Functional components of PSII, PSI and the Cyt b_6/f complex were determined by spectroscopic methods using thylakoids isolated from the plants. The data represent the mean ± SE of n = 3 experiments. A statistical analysis of the data is contained in Doc S2 Table 13.
