# Supplementary material for: The Combined Loss of Triose Phosphate and Xylulose 5-Phosphate/Phosphate Translocators Leads to Severe Growth Retardation and Impaired Photosynthesis in Arabidopsis thaliana tpt/xpt Double Mutants
Source: Front Plant Sci. 2018 Oct 2;9:1331. doi: 10.3389/fpls.2018.01331 (PMC6175978; doi:10.3389/fpls.2018.01331)
Supplement: DATE SHEET S2 — Contains 6 Supplementary Tables. [file Data_Sheet_2.pdf]

Supplementary Tables

Supplementary Table 1. Oligonucleotide Primers used for mutant screening (A) and verification (B). The sequences are given from the 5´ to 3´ end.

A Screening

	***gpt2-1* mutant**
GPT2-1 (f)	GTCGGACCAAACTTTGTCTGGT
GPT2-1 (r)	GGTCTGATCAAGAAATGACACTGA
LB_{gpt2-1} (f)	ATATTGACCATCATACTCATTGC
	***tpt-1* mutant**
TPT-1 (f)	GTAACTTACGAGTAAACTGGCTAC
TPT-1 (r)	AGCAGCCGCATTGAAGAATGGCTC AA
LB_{tpt-1} (f)	GATGCACTCGAAATCAGCCAATTTTAGAC
	***tpt-2* mutant**
TPT-2 (f)	GTAACTTACGAGTAAACTGGCTAC
TPT-2 B (f)	GACCATTAACCCTACCATAACTCC
TPT-2 (r)	TGACTAGCCATGGATACTTGGCGAGGA
LB_{tpt-2} (f)	GTCCGCAATGTGTTATTAAGTTGTC
	***xpt-1* mutant**
XPT-1 (f)	GGCTTTCACCGATTCCCAA
XPT-1 (r)	ATCAAGTAGACGAGGTCAAGAACTAAGTA
LB_{xpt-1} (f)	CATTTTATAATAACGCTGCGGACATCTAC
	***xpt-2* mutant**
XPT-2 (f)	AAAAGACAAATGATGGCATCG
XPT-2 (r)	TAACGGATACGAATCACCGAG
	***xpt-3* mutant**
XPT-3 (f)	CTTTTTCTCATCTCCTGCGTG
XPT-3 (r)	TCTGGATTTGAATCCGATGAG
LB3 (pCSA110)	TAGCATCTGAATTTCATAACCAATCTCGATACAC
	**amiRNA:*XPT* construction and verification**
XPT, miR-s I	GATAATGGCTTTATGGTAACCTCTCTCTCTTTTGTATTCC
XPT, miR-a II	GAGAGGTTACCATAAAGCCATTATCAAAGAGAATCAATGA
XPT, miR*s III	GAGAAGTTACCATAATGCCATTTTCACAGGTCGTGATAT G
XPT, miR*a IV	GAAAATGGCATTATGGTAACTTCTCTACATATATATTCC T
pRS300A TOPO (f)	*CACC* CTGCAAGGCGATTAAGTTGGGTAAC
pRS300B (r)	GCGGATAACAATTTCACACAGGAAACAG
CaMV 35S (f)	GCAAGACCCTTCCTCTATATAAG

B RT-PCR

RT_*XPT* (f)*	*CACC* ATGATCTCCCTGAATCTATCTCCT
RT_*XPT* (r)	TTAGTTCTTCTTATCACCTCCCACTTCAATC
RT_Actin2 (f)	TTGGTAGGCCAAGACATCAT
RT_Actin2 (r)	GGAGCCTCGGTAAGAAGAAC

*The same primer was used for TOPO cloning. (f) = forward; (r) = reverse

Supplementary Table 2. Chl *a* fluorescence parameters of wild-type and mutant plants impaired in XPT and TPT as well as starch biosynthesis.

Biotype	F_v/F_m	$\Phi PSII_{(281)}$	Relative $ETR_{(700)}$
Ws-2	0.778 ± 0.001	0.405 ± 0.005	79.53 ± 1.35
xpt-1	0.787 ± 0.002	0.409 ± 0.002	80.67 ± 0.92
tpt-1	0.743 ± 0.008	0.366 ± 0.010	60.71 ± 2.45
tpt-1/xpt-1	0.544 ± 0.022	0.229 ± 0.017	28.19 ± 2.93
Col-0	0.769 ± 0.005	0.407 ± 0.012	71.32 ± 3.40
tpt-2	0.751 ± 0.003	0.414 ± 0.008	67.39 ± 1.32
tpt-2/xpt-1	0.492 ± 0.022	0.192 ± 0.010	19.64 ± 2.77
amiRNA:*XPT tpt-2* #3	0.701 ± 0.041	0.361 ± 0.033	58.56 ± 6.29
amiRNA:*XPT tpt-2* #4	0.654 ± 0.041	0.249 ± 0.029	27.08 ± 6.15
adg1-1/tpt-2	0.344 ± 0.017	0.139 ± 0.009	18.03 ± 1.31

The plants were grown in soil for three weeks in a temperature-controlled growth cabinet under HL-conditions (PFD = 300 $\mu mol \cdot m^{-2} \cdot s^{-1}$) in the long-day. The Chl *a* fluorescence parameters were determined after plants were darkened for about 30 min by applying a saturating light flash. Photosynthesis was induced with actinic light at a PFD of 281 $\mu mol \cdot m^{-2} \cdot s^{-1}$ for 5 min. ΦPSII was determined at the PFD used for the induction of photosynthesis. $ETR_{(700)}$ was determined at a PFD of 700 $\mu mol \cdot m^{-2} \cdot s^{-1}$.The data represent the mean ± SE of n = 9 measurements. A statistical analysis of the data is contained in Doc S2, Table 3B.

Supplementary Table 3. Pigment and protein contents as well as specific leaf fresh weights of wild-type, mutant, and amiRNA plants defective in the TPT and/or XPT.

Plant line	Chl content	Carotenoid content	Chl *a/b*-ratio	Protein content	Specific fw
	($mg \cdot m^{-2}$)				($g \cdot m^{-2}$)
Ws-2	137.8 ± 4.6	20.28 ± 1.38	3.38 ± 0.04	1.28 ± 0.10	98.98 ± 2.22
xpt-1	139.2 ± 5.2	20.13 ± 1.47	3.31 ± 0.06	1.62 ± 0.13	115.15 ± 3.33
tpt-1	131.1 ± 5.5	20.41 ± 1.44	3.40 ± 0.06	1.61 ± 0.08	99.96 ± 3.10
tpt-1/xpt-1	127.1 ± 6.1	20.48 ± 1.55	3.47 ± 0.09	1.28 ± 0.21	107.23 ± 4.09
Col-0	136.6 ± 1.2	18.49 ± 0.72	3.40 ± 0.08	1.61 ± 0.16	120.81 ± 5.02
tpt-2	134.5 ± 4.4	20.50 ± 0.78	3.52 ± 0.03	1.54 ± 0.29	118.90 ± 12.76
tpt-2/xpt-1	127.8 ± 1.2	21.26 ± 0.35	3.62 ± 0.03	1.54 ± 0.07	114.58 ± 16.23
amiRNA:*XPT tpt-2* #3	136.6 ± 5.1	19.50 ± 1.40	3.35 ± 0.02	1.18 ± 0.11	106.45 ± 2.34
amiRNA:*XPT tpt-2* #4	132.3 ± 4.6	20.46 ± 1.30	3.36 ± 0.03	1.52 ± 0.16	107.64 ± 3.58
adg1-1/tpt-2	110.6 ± 5.0	19.97 ± 0.82	2.96 ± 0.12	1.31 ± 0.10	101.44 ± 3.87

The plants were grown for for 33 days after sowing under LL-conditions (i.e. 30 $\mu mol \cdot m^{-2} \cdot s^{-1}$) in the long-day. Pigment and protein contents are expressed on a leaf area basis. The specific leaf fresh weight allows a conversion from a leaf area to a leaf weight basis. For the Chl *a/b*-ratios, Chl *a* and *b* were determined separately and the quotient calculated for each replicate. The data represent the mean ± SE of n = 4 to 20 replicates. A statistical analysis of the data is contained in Doc S2 Table 3.

Supplementary Table 4. Metabolite contents in leaves of wild-type and mutant plants based on LC-MS/MS analyses.

A Middle of the dark period (5 h in the dark)

**Metabolite content**	**Ws-2**	***xpt-1***	***tpt-1/xpt-1***	**Col-0**	***tpt-2***	***tpt-2/xpt-1***
5 h in the dark			nmol·g^{-1} fw			
**3-PGA**	67.8 ± 23.5	56.5 ± 49.6	58.6 ± 16.4	69.9 ± 26.3	**246.3 ± 60.6**	90.8 ± 57.4
DHAP	8.0 ± 3.1	6.3 ± 3.4	10.4 ± 1.0	8.2 ± 1.6	9.8 ± 4.0	7.2 ± 4.4
**S-7-P**	19.7 ± 8.4	21.5 ± 9.4	**71.5 ± 15.5**	13.5 ± 1.6	20.4 ± 2.6	29.3 ± 15.6
Ri5P	3.5 ± 0.3	4.1 ± 0.7	6.2 ± 0.5	2.1 ± 0.6	2.9 ± 1.0	4.9 ± 2.3
**Xu5P+Ru5P**	5.2 ± 1.7	7.1 ± 2.7	**27.5 ± 3.0**	4.0 ± 1.1	6.6 ± 1.1	**26.9 ± 6.3**
Fru6P	46.5 ± 10.7	45.3 ± 14.9	37.4 ± 16.7	40.3 ± 9.5	53.5 ± 6.3	43.3 ± 11.3
Glc6P	207.0 ± 74.5	153.2 ± 85.3	159.0 ± 13.1	193.6 ± 35.9	179.2 ± 73.0	141.7 ± 47.0
Glc1P	37.3 ± 3.4	33.9 ± 2.0	36.1 ± 3.0	30.5 ± 5.9	40.6 ± 5.5	35.4 ± 5.0
UDPG	137.2 ± 10.9	123.7 ± 9.1	125.1 ± 39.6	130.5 ± 14.6	143.4 ± 17.7	118.4 ± 18.8
Glycerol-3-P	9.0 ± 2.5	9.5 ± 2.5	8.3 ± 3.9	10.0 ± 2.8	16.4 ± 3.6	13.1 ± 5.5
ATP	16.1 ± 2.5	12.1 ± 3.6	13.4 ± 5.6	12.6 ± 5.2	16.8 ± 3.8	12.3 ± 3.7
AMP	23.4 ± 3.0	24.5 ± 5.0	23.6 ± 5.3	15.3 ± 6.3	18.6 ± 3.3	16.8 ± 1.3
**Asn**	134.3 ± 18.5	107.6 ± 25.3	101.1 ± 19.5	83.2 ± 23.1	84.8 ± 14.0	**73.3 ± 16.4**
**Phe**	30.6 ± 7.0	26.8 ± 3.3	22.7 ± 3.3	20.1 ± 2.6	25.4 ± 4.7	**46.1 ± 2.4**
**Tyr**	4.1 ± 1.6	4.1 ± 1.2	4.9 ± 1.6	3.1 ± 0.8	4.2 ± 0.8	**11.7 ± 1.3**
**Trp**	3.0 ± 1.0	3.3 ± 1.2	4.7 ± 1.8	2.2 ± 0.8	3.9 ± 1.0	**9.7 ± 4.0**

B Beginning of the light period (1 h in the light)

**Metabolite content**	**Ws-2**	***xpt-1***	***tpt-1/xpt-1***	**Col-0**	***tpt-2***	***tpt-2/xpt-1***
1 h in the light			nmol·g^{-1} fw			
3-PGA	48.4 ± 34.3	51.1 ± 14.8	47.2 ± 28.9	90.1 ± 31.7	84.3 ± 26.7	112.4 ± 69.8
DHAP	35.3 ± 15.5	30.8 ± 14.1	34.9 ± 10.2	34.3 ± 13.9	49.3 ± 11.2	26.9 ± 14.7
S-7-P	116.6 ± 34.2	101.1 ± 37.2	118.8 ± 27.5	98.1 ± 25.3	120.0 ± 39.7	68.4 ± 42.9
Ri5P	4.9 ± 1.0	4.4 ± 0.1	7.7 ± 4.4	3.0 ± 1.4	4.6 ± 1.8	16.7 ± 11.5
**Xu5P+Ru5P**	32.3 ± 12.3	29.0 ± 9.5	**135.8 ± 40.4**	22.5 ± 4.4	73.8 ± 15.6	**102.6 ± 56.3**
Fru6P	62.9 ± 17.4	55.8 ± 15.9	51.3 ± 16.8	58.5 ± 9.4	62.4 ± 9.3	48.3 ± 17.8
Glc6P	121.2 ± 59.5	148.8 ± 12.1	124.6 ± 46.6	121.2 ± 51.9	153.2 ± 32.3	97.0 ± 63.7
Glc1P	29.9 ± 1.5	25.5 ± 6.3	24.6 ± 3.0	26.7 ± 5.0	30.6 ± 7.5	31.4 ± 6.1
UDPG	116.3 ± 5.3	102.4 ± 22.2	99.3 ± 29.9	103.8 ± 19.6	113.4 ± 17.1	123.8 ± 39.6
Glycerol-3-P	10.7 ± 3.4	10.7 ± 3.5	8.5 ± 3.5	11.0 ± 1.1	15.8 ± 2.2	13.0 ± 4.8
ATP	17.6 ± 5.4	13.8 ± 3.9	15.6 ± 5.4	18.9 ± 5.3	18.9 ± 4.9	24.4 ± 17.1
AMP	25.4 ± 0.2	28.5 ± 6.9	31.0 ± 4.5	18.0 ± 7.2	24.2 ± 5.8	21.2 ± 11.4
Asn	119.4 ± 29.1	128.7 ± 39.7	128.2 ± 18.9	94.9 ± 32.1	93.7 ± 16.0	81.6 ± 41.3
**Phe**	62.8 ± 6.4	48.4 ± 11.2	**31.1 ± 3.5**	54.0 ± 11.3	47.7 ± 10.5	57.0 ± 10.4
**Tyr**	5.8 ± 1.0	5.3 ± 1.5	5.4 ± 1.3	5.3 ± 1.1	6.7 ± 1.3	**15.4 ± 4.2**
**Trp**	4.2 ± 0.7	3.9 ± 1.5	4.1 ± 0.9	3.2 ± 1.2	5.5 ± 1.4	**13.4 ± 4.3**

Supplementary Table 4 (continued)

C Middle of the light period (8 h in the light)

**Metabolite content**	**Ws-2**	***xpt-1***	***tpt-1/xpt-1***	**Col-0**	***tpt-2***	***tpt-2/xpt-1***
Middle of the day			$nmol \cdot g^{-1}$ fw			
3-PGA	147.2 ± 71.4	80.8 ± 46.0	47.2 ± 38.4	75.4 ± 35.5	147.0 ± 53.0	50.5 ± 50.0
DHAP	46.7 ± 23.0	29.7 ± 14.7	25.9 ± 17.8	34.6 ± 10.4	44.1 ± 21.0	22.5 ± 12.4
S-7-P	166.2 ± 62.8	101.3 ± 36.3	81.7 ± 53.1	109.8 ± 21.6	144.1 ± 56.4	59.9 ± 27.6
**Ri5P**	4.6 ± 2.3	3.5 ± 1.8	11.6 ± 8.4	2.8 ± 1.0	6.9 ± 2.6	**25.0 ± 9.9**
**Xu5P+Ru5P**	33.8 ± 10.8	30.4 ± 8.1	**190.2 ± 78.0**	27.4 ± 7.2	95.0 ± 16.3	**152.5 ± 41.4**
Fru6P	83.8 ± 24.2	56.4 ± 10.6	49.0 ± 21.5	62.9 ± 9.2	75.3 ± 15.7	52.2 ± 16.0
Glc6P	155.1 ± 75.7	135.0 ± 35.9	108.0 ± 69.1	112.2 ± 32.3	196.6 ± 39.9	79.3 ± 42.1
Glc1P	31.4 ± 4.1	24.4 ± 1.2	28.4 ± 1.5	26.4 ± 3.3	33.8 ± 5.4	28.6 ± 4.8
UDPG	105.3 ± 34.8	101.2 ± 7.7	114.2 ± 12.9	123.5 ± 19.0	111.1 ± 33.1	107.2 ± 28.6
**Glycerol-3-P**	12.8 ± 3.4	11.7 ± 2.6	**6.0 ± 2.9**	13.7 ± 3.3	20.0 ± 2.7	14.1 ± 4.3
ATP	25.6 ± 10.6	18.9 ± 5.5	21.8 ± 10.3	20.5 ± 7.2	24.2 ± 7.1	21.0 ± 6.5
AMP	22.6 ± 6.0	25.6 ± 3.0	33.6 ± 6.3	19.6 ± 6.7	26.9 ± 3.4	19.0 ± 10.9
Asn	162.7 ± 46.1	128.1 ± 36.4	129.2 ± 15.0	85.4 ± 21.1	121.6 ± 20.9	113.5 ± 31.4
**Phe**	62.4 ± 6.4	**44.5 ± 4.7**	**34.0 ± 3.8**	47.2 ± 5.2	47.1 ± 7.5	51.8 ± 4.8
**Tyr**	6.6 ± 1.2	5.3 ± 0.8	4.8 ± 1.1	5.4 ± 0.9	6.0 ± 1.4	**12.1 ± 1.7**
Trp	3.8 ± 0.7	3.5 ± 0.7	4.3 ± 1.2	3.1 ± 0.7	4.7 ± 1.5	7.6 ± 4.4

The plants were grown in soil under HL-conditions (PFD = 300 $\mu mol \cdot m^{-2} \cdot s^{-1}$) in the long-day. Samples were taken at the end of the dark period (**A**), the beginning (**B**), and middle of the light period (**C**). The data derive from the mean ± SE of n = 3 to 5 independent samples. Bold numbers indicate significantly increased (red) or decreased (green) contents.

Supplementary Table 5. Metabolite contents in leaves of wild-type and mutant plants based on GC-MS analyses.

A Middle of the dark period (5 h in the dark)

**Metabolite content**	**Ws-2**	***xpt-1***	***tpt-1/xpt-1***	**Col-0**	***tpt-2***	***tpt-2/xpt-1***
5 h in the dark			Arbitrary units·g^{-1} fw			
Glu	0.189 ± 0.046	0.180 ± 0.050	0.103 ± 0.036	0.165 ± 0.050	0.134 ± 0.032	0.024 ± 0.006
Asp	0.389 ± 0.075	0.363 ± 0.095	0.331 ± 0.066	0.205 ± 0.081	0.249 ± 0.060	0.076 ± 0.021
Ser	0.231 ± 0.072	0.254 ± 0.082	0.152 ± 0.039	0.020 ± 0.003	0.164 ± 0.028	0.226 ± 0.122
**Gly**	0.079 ± 0.032	**0.161 ± 0.051**	**0.002 ± 0.000**	0.011 ± 0.001	0.008 ± 0.004	0.010 ± 0.002
Thr	0.181 ± 0.059	0.173 ± 0.043	0.089 ± 0.017	0.099 ± 0.043	0.100 ± 0.027	0.155 ± 0.063
Pro	0.208 ± 0.082	0.143 ± 0.038	0.094 ± 0.023	0.035 ± 0.006	0.133 ± 0.031	0.068 ± 0.025
Phe	0.227 ± 0.067	0.173 ± 0.063	0.060 ± 0.010	0.182 ± 0.060	0.199 ± 0.033	0.090 ± 0.033
**Tyr**	0.236 ± 0.075	0.193 ± 0.055	0.219 ± 0.055	0.183 ± 0.019	0.291 ± 0.052	**0.509 ± 0.102**
Val	0.245 ± 0.039	0.245 ± 0.060	0.234 ± 0.080	0.147 ± 0.035	0.377 ± 0.059	0.296 ± 0.055
Leu	0.284 ± 0.097	0.291 ± 0.084	0.330 ± 0.121	0.243 ± 0.092	0.537 ± 0.091	0.306 ± 0.048
Ile	0.124 ± 0.047	0.130 ± 0.038	0.116 ± 0.021	0.045 ± 0.008	0.095 ± 0.027	0.171 ± 0.020
Lys	0.620 ± 0.149	0.516 ± 0.131	0.321 ± 0.138	0.312 ± 0.098	0.210 ± 0.056	0.377 ± 0.091
Met	0.213 ± 0.061	0.177 ± 0.065	0.070 ± 0.028	0.135 ± 0.061	0.186 ± 0.033	0.032 ± 0.010
**β-Ala**	0.175 ± 0.064	0.065 ± 0.027	0.099 ± 0.040	0.109 ± 0.029	0.354 ± 0.076	**0.677 ± 0.083**
Glycerol	0.351 ± 0.120	0.253 ± 0.077	0.323 ± 0.108	0.252 ± 0.079	0.324 ± 0.072	0.345 ± 0.101
**Mannitol**	0.167 ± 0.064	0.043 ± 0.012	0.167 ± 0.060	0.050 ± 0.018	0.167 ± 0.052	**0.295 ± 0.065**
Myoinositol	0.695 ± 0.086	0.663 ± 0.132	0.538 ± 0.102	0.333 ± 0.080	0.254 ± 0.043	0.423 ± 0.125
Sorbitol	0.153 ± 0.058	0.048 ± 0.009	0.088 ± 0.019	0.075 ± 0.030	0.150 ± 0.061	0.046 ± 0.017
Putrescine	0.414 ± 0.118	0.332 ± 0.086	0.222 ± 0.062	0.337 ± 0.099	0.297 ± 0.039	0.400 ± 0.063
(Iso) Citrate	0.300 ± 0.083	0.599 ± 0.243	0.445 ± 0.098	0.127 ± 0.028	0.292 ± 0.115	0.284 ± 0.068
**2-Oxoglutarate**	0.120 ± 0.022	0.186 ± 0.048	**0.299 ± 0.043**	0.077 ± 0.024	**0.250 ± 0.052**	**0.388 ± 0.069**
**Succinate**	0.154 ± 0.031	0.185 ± 0.075	0.194 ± 0.049	0.095 ± 0.018	0.400 ± 0.128	**0.807 ± 0.075**
**Fumarate**	0.458 ± 0.120	0.526 ± 0.112	0.221 ± 0.094	0.332 ± 0.076	0.206 ± 0.050	**0.080 ± 0.044**
Malate	0.201 ± 0.062	0.163 ± 0.046	0.139 ± 0.037	0.088 ± 0.019	0.200 ± 0.033	0.121 ± 0.041
Glycolate	0.231 ± 0.039	0.153 ± 0.014	0.228 ± 0.043	0.106 ± 0.055	0.193 ± 0.023	0.237 ± 0.064
Lactate	0.349 ± 0.113	0.428 ± 0.114	0.619 ± 0.112	0.323 ± 0.111	0.595 ± 0.091	0.377 ± 0.024
**Glycerate**	0.117 ± 0.016	0.157 ± 0.040	**0.470 ± 0.048**	0.018 ± 0.007	0.203 ± 0.019	**0.571 ± 0.142**
**Gluconate**	0.038 ± 0.013	0.025 ± 0.010	0.099 ± 0.042	0.012 ± 0.004	0.023 ± 0.009	**0.321 ± 0.041**
Maleate	0.312 ± 0.070	0.234 ± 0.070	0.272 ± 0.064	0.129 ± 0.032	0.222 ± 0.026	0.249 ± 0.053
**Malonate**	0.199 ± 0.063	0.086 ± 0.028	**0.327 ± 0.077**	0.146 ± 0.066	0.250 ± 0.072	**0.464 ± 0.075**
**Shikimate**	0.315 ± 0.045	0.370 ± 0.083	0.249 ± 0.047	0.332 ± 0.102	0.442 ± 0.072	**0.106 ± 0.041**
Quinate	0.168 ± 0.060	0.053 ± 0.021	0.197 ± 0.083	0.283 ± 0.151	0.165 ± 0.074	0.161 ± 0.062
**Glc**	0.333 ± 0.072	0.382 ± 0.069	0.393 ± 0.064	0.075 ± 0.037	0.285 ± 0.029	**0.621 ± 0.148**
**Fru**	0.111 ± 0.031	0.182 ± 0.034	**0.433 ± 0.060**	0.019 ± 0.015	0.186 ± 0.058	**0.571 ± 0.078**
Suc	0.416 ± 0.074	0.316 ± 0.083	0.215 ± 0.053	0.246 ± 0.048	0.370 ± 0.034	0.453 ± 0.145
**Maltose**	0.177 ± 0.010	0.208 ± 0.047	**0.499 ± 0.075**	0.211 ± 0.038	**0.872 ± 0.054**	**0.842 ± 0.061**
Mannose	0.460 ± 0.051	0.599 ± 0.113	0.460 ± 0.059	0.214 ± 0.070	0.362 ± 0.025	0.661 ± 0.112
Xylose	0.268 ± 0.056	0.420 ± 0.080	0.317 ± 0.057	0.212 ± 0.058	0.247 ± 0.018	0.387 ± 0.102

Supplementary Table 5 (continued)

B Beginning of the light period (1 h in the light)

**Metabolite content**	**Ws-2**	***xpt-1***	***tpt-1/xpt-1***	**Col-0**	***tpt-2***	***tpt-2/xpt-1***
1 h in the light			Arbitrary units·g^{-1} fw			
**Glu**	0.377 ± 0.050	0.330 ± 0.043	**0.045 ± 0.016**	0.243 ± 0.078	0.284 ± 0.074	0.206 ± 0.065
Asp	0.382 ± 0.142	0.301 ± 0.049	0.266 ± 0.046	0.261 ± 0.089	0.493 ± 0.116	0.504 ± 0.069
Ser	0.212 ± 0.074	0.225 ± 0.066	0.220 ± 0.086	0.097 ± 0.025	0.346 ± 0.086	0.212 ± 0.088
**Gly**	0.218 ± 0.039	0.252 ± 0.038	**0.098 ± 0.013**	0.088 ± 0.020	**0.141 ± 0.014**	**0.063 ± 0.013**
Thr	0.206 ± 0.057	0.191 ± 0.059	0.180 ± 0.077	0.151 ± 0.031	0.258 ± 0.049	0.213 ± 0.044
Pro	0.187 ± 0.042	0.107 ± 0.020	0.147 ± 0.065	0.085 ± 0.025	0.128 ± 0.030	0.198 ± 0.026
**Phe**	0.713 ± 0.092	0.612 ± 0.079	**0.090 ± 0.024**	0.549 ± 0.099	0.383 ± 0.063	0.301 ± 0.096
**Tyr**	0.379 ± 0.072	0.521 ± 0.133	0.385 ± 0.077	0.264 ± 0.032	0.391 ± 0.083	**0.721 ± 0.094**
**Val**	0.202 ± 0.033	**0.150 ± 0.052**	0.214 ± 0.061	0.155 ± 0.025	0.331 ± 0.080	**0.520 ± 0.028**
**Leu**	0.214 ± 0.051	**0.159 ± 0.053**	0.216 ± 0.091	0.169 ± 0.042	0.335 ± 0.116	**0.551 ± 0.077**
**Ile**	0.208 ± 0.034	**0.165 ± 0.044**	0.248 ± 0.081	0.198 ± 0.032	0.320 ± 0.058	**0.471 ± 0.056**
Lys	0.625 ± 0.078	0.373 ± 0.098	0.542 ± 0.174	0.382 ± 0.036	0.212 ± 0.093	0.664 ± 0.166
Met	0.395 ± 0.087	0.317 ± 0.061	0.225 ± 0.039	0.330 ± 0.072	0.523 ± 0.140	0.172 ± 0.039
**β-Ala**	0.111 ± 0.033	0.127 ± 0.051	**0.061 ± 0.028**	0.150 ± 0.040	0.247 ± 0.053	**0.833 ± 0.076**
Glycerol	0.390 ± 0.154	0.359 ± 0.079	0.221 ± 0.069	0.249 ± 0.093	0.345 ± 0.132	0.269 ± 0.079
Mannitol	0.155 ± 0.051	0.247 ± 0.107	0.235 ± 0.069	0.141 ± 0.041	0.129 ± 0.021	0.302 ± 0.082
Myoinositol	0.656 ± 0.089	0.579 ± 0.093	0.541 ± 0.074	0.197 ± 0.043	0.215 ± 0.058	0.359 ± 0.107
Sorbitol	0.089 ± 0.034	0.222 ± 0.117	0.038 ± 0.006	0.168 ± 0.074	0.037 ± 0.018	0.069 ± 0.021
Putrescine	0.281 ± 0.063	0.266 ± 0.068	0.267 ± 0.086	0.253 ± 0.066	0.353 ± 0.086	0.321 ± 0.074
(Iso) Citrate	0.373 ± 0.186	0.286 ± 0.114	0.290 ± 0.027	0.186 ± 0.062	0.296 ± 0.099	0.390 ± 0.105
**2-Oxoglutarate**	0.293 ± 0.068	0.309 ± 0.062	0.241 ± 0.010	0.126 ± 0.021	0.211 ± 0.043	**0.454 ± 0.108**
**Succinate**	0.135 ± 0.052	0.253 ± 0.920	0.121 ± 0.022	0.034 ± 0.009	**0.207 ± 0.057**	**0.682 ± 0.093**
**Fumarate**	0.463 ± 0.111	0.644 ± 0.118	**0.221 ± 0.055**	0.384 ± 0.072	0.216 ± 0.046	**0.107 ± 0.042**
Malate	0.208 ± 0.067	0.228 ± 0.038	0.168 ± 0.021	0.140 ± 0.028	0.248 ± 0.043	0.249 ± 0.095
Glycolate	0.399 ± 0.096	0.364 ± 0.049	0.333 ± 0.097	0.476 ± 0.139	0.337 ± 0.049	0.318 ± 0.090
Lactate	0.614 ± 0.150	0.457 ± 0.054	0.447 ± 0.095	0.387 ± 0.176	0.459 ± 0.120	0.318 ± 0.120
Glycerate	0.350 ± 0.069	0.399 ± 0.066	0.423 ± 0.054	0.137 ± 0.013	0.196 ± 0.049	0.381 ± 0.110
**Gluconate**	0.019 ± 0.008	0.009 ± 0.003	0.049 ± 0.013	0.010 ± 0.004	0.018 ± 0.007	**0.520 ± 0.048**
Maleate	0.361 ± 0.152	0.358 ± 0.057	0.262 ± 0.072	0.178 ± 0.061	0.218 ± 0.057	0.282 ± 0.075
Malonate	0.065 ± 0.007	0.078 ± 0.026	0.358 ± 0.109	0.050 ± 0.015	0.534 ± 0.224	0.448 ± 0.056
**Shikimate**	0.512 ± 0.095	0.515 ± 0.043	0.400 ± 0.027	0.344 ± 0.049	0.398 ± 0.139	**0.130 ± 0.031**
Quinate	0.228 ± 0.098	0.163 ± 0.104	0.022 ± 0.005	0.121 ± 0.052	0.021 ± 0.006	0.085 ± 0.030
Glc	0.528 ± 0.062	0.618 ± 0.116	0.505 ± 0.044	0.325 ± 0.051	0.366 ± 0.049	0.657 ± 0.133
Fru	0.705 ± 0.076	0.727 ± 0.076	0.430 ± 0.041	0.455 ± 0.070	0.302 ± 0.046	0.659 ± 0.115
**Suc**	0.425 ± 0.061	0.386 ± 0.102	0.128 ± 0.038	0.241 ± 0.035	0.345 ± 0.036	**0.594 ± 0.162**
**Maltose**	0.026 ± 0.006	0.041 ± 0.013	0.124 ± 0.012	0.020 ± 0.005	0.136 ± 0.051	**0.701 ± 0.105**
Mannose	0.326 ± 0.041	0.342 ± 0.078	0.214 ± 0.033	0.143 ± 0.030	0.157 ± 0.031	0.381 ± 0.093
Xylose	0.289 ± 0.036	0.410 ± 0.131	0.338 ± 0.056	0.203 ± 0.049	0.274 ± 0.039	0.419 ± 0.103

Supplementary Table 5 (continued)

C Middle of the light period (8 h in the light)

**Metabolite content**	**Ws-2**	***xpt-1***	***tpt-1/xpt-1***	**Col-0**	***tpt-2***	***tpt-2/xpt-1***
8 h in the light			Arbitrary units·g^{-1} fw			
**Glu**	0.423 ± 0.039	0.371 ± 0.038	**0.059 ± 0.026**	0.294 ± 0.081	0.249 ± 0.068	0.209 ± 0.090
Asp	0.549 ± 0.140	0.302 ± 0.038	0.374 ± 0.077	0.311 ± 0.040	0.314 ± 0.048	0.443 ± 0.156
Ser	0.536 ± 0.143	0.388 ± 0.164	0.386 ± 0.156	0.177 ± 0.023	0.363 ± 0.113	0.412 ± 0.149
**Gly**	0.716 ± 0.119	0.517 ± 0.064	**0.155 ± 0.030**	0.299 ± 0.056	0.303 ± 0.041	**0.100 ± 0.023**
Thr	0.502 ± 0.150	0.275 ± 0.096	0.355 ± 0.098	0.277 ± 0.036	0.359 ± 0.106	0.517 ± 0.164
**Pro**	0.648 ± 0.148	0.145 ± 0.043	0.155 ± 0.032	0.152 ± 0.038	0.255 ± 0.083	0.239 ± 0.075
**Phe**	0.660 ± 0.132	0.528 ± 0.063	**0.139 ± 0.045**	0.463 ± 0.076	0.442 ± 0.133	**0.194 ± 0.061**
Tyr	0.481 ± 0.103	0.392 ± 0.073	0.279 ± 0.043	0.278 ± 0.091	0.255 ± 0.065	0.617 ± 0.098
**Val**	0.459 ± 0.144	**0.088 ± 0.017**	**0.281 ± 0.024**	0.156 ± 0.037	**0.369 ± 0.054**	**0.627 ± 0.106**
**Leu**	0.468 ± 0.132	**0.093 ± 0.008**	**0.169 ± 0.037**	0.193 ± 0.037	**0.391 ± 0.091**	**0.672 ± 0.142**
**Ile**	0.487 ± 0.128	**0.123 ± 0.022**	**0.321 ± 0.032**	0.269 ± 0.043	**0.502 ± 0.090**	**0.689 ± 0.137**
Lys	0.713 ± 0.108	0.547 ± 0.086	0.581 ± 0.069	0.382 ± 0.025	0.304 ± 0.086	0.574 ± 0.156
**Met**	0.710 ± 0.130	0.410 ± 0.047	**0.241 ± 0.038**	0.466 ± 0.083	0.460 ± 0.136	**0.205 ± 0.087**
**β-Ala**	0.160 ± 0.024	**0.084 ± 0.015**	0.157 ± 0.059	0.243 ± 0.018	**0.436 ± 0.076**	**0.798 ± 0.075**
Glycerol	0.315 ± 0.096	0.237 ± 0.070	0.295 ± 0.074	0.331 ± 0.103	0.422 ± 0.091	0.618 ± 0.126
**Mannitol**	0.166 ± 0.039	0.132 ± 0.037	**0.373 ± 0.105**	0.293 ± 0.077	0.332 ± 0.085	**0.595 ± 0.157**
**Myoinositol**	0.739 ± 0.122	0.544 ± 0.068	0.709 ± 0.137	0.236 ± 0.061	0.153 ± 0.056	**0.413 ± 0.118**
Sorbitol	0.058 ± 0.023	0.035 ± 0.009	0.098 ± 0.023	0.110 ± 0.042	0.158 ± 0.055	0.074 ± 0.028
Putrescine	0.555 ± 0.133	0.411 ± 0.095	0.227 ± 0.043	0.497 ± 0.082	0.561 ± 0.023	0.474 ± 0.091
(Iso) Citrate	0.108 ± 0.036	0.169 ± 0.039	0.237 ± 0.058	0.155 ± 0.056	0.202 ± 0.061	0.464 ± 0.183
2-Oxoglutarate	0.630 ± 0.108	0.447 ± 0.063	0.358 ± 0.060	0.296 ± 0.024	0.453 ± 0.059	0.574 ± 0.127
**Succinate**	0.100 ± 0.025	0.166 ± 0.041	**0.304 ± 0.091**	0.187 ± 0.044	**0.345 ± 0.043**	**0.648 ± 0.084**
**Fumarate**	0.705 ± 0.105	0.790 ± 0.079	0.391 ± 0.128	0.572 ± 0.056	**0.280 ± 0.080**	**0.158 ± 0.078**
Malate	0.460 ± 0.146	0.285 ± 0.059	0.291 ± 0.076	0.263 ± 0.064	0.344 ± 0.115	0.462 ± 0.165
Glycolate	0.585 ± 0.119	0.382 ± 0.046	0.414 ± 0.062	0.421 ± 0.029	0.419 ± 0.066	0.531 ± 0.083
**Lactate**	0.448 ± 0.126	0.212 ± 0.086	0.567 ± 0.108	0.372 ± 0.062	0.485 ± 0.095	**0.726 ± 0.093**
Glycerate	0.526 ± 0.177	0.536 ± 0.045	0.486 ± 0.075	0.243 ± 0.052	0.404 ± 0.121	0.330 ± 0.100
**Gluconate**	0.010 ± 0.002	0.018 ± 0.008	0.041 ± 0.016	0.005 ± 0.001	0.007 ± 0.001	**0.659 ± 0.143**
Maleate	0.413 ± 0.148	0.359 ± 0.113	0.463 ± 0.095	0.313 ± 0.092	0.248 ± 0.073	0.373 ± 0.109
**Malonate**	0.124 ± 0.029	**0.087 ± 0.006**	**0.292 ± 0.039**	0.201 ± 0.072	0.268 ± 0.059	**0.753 ± 0.105**
Shikimate	0.822 ± 0.116	0.523 ± 0.048	0.442 ± 0.074	0.690 ± 0.132	0.630 ± 0.149	0.355 ± 0.114
**Quinate**	0.026 ± 0.003	**0.291 ± 0.085**	**0.045 ± 0.010**	0.018 ± 0.004	0.075 ± 0.029	**0.096 ± 0.027**
Glc	0.555 ± 0.119	0.615 ± 0.111	0.461 ± 0.070	0.399 ± 0.107	0.202 ± 0.039	0.537 ± 0.119
**Fru**	0.467 ± 0.111	0.524 ± 0.102	0.333 ± 0.032	0.178 ± 0.045	0.117 ± 0.018	**0.661 ± 0.097**
**Suc**	0.739 ± 0.127	0.528 ± 0.062	**0.213 ± 0.038**	0.416 ± 0.097	0.304 ± 0.074	0.423 ± 0.089
**Maltose**	0.047 ± 0.013	0.042 ± 0.011	**0.185 ± 0.022**	0.028 ± 0.006	0.092 ± 0.018	**0.484 ± 0.056**
Mannose	0.428 ± 0.129	0.460 ± 0.092	0.383 ± 0.023	0.244 ± 0.047	0.185 ± 0.053	0.415 ± 0.108
Xylose	0.533 ± 0.132	0.459 ± 0.060	0.462 ± 0.042	0.296 ± 0.033	0.252 ± 0.034	0.516 ± 0.134

The plants were grown in soil under HL-conditions (PFD = 300 µmol·m^{-2}·s^{-1}) in the long-day. Samples were taken at the end of the dark period (**A**), the beginning (**B**) and middle of the light period (**C**) The data derive from the mean ± SE of n = 3 to 5 independent samples. Bold numbers indicate significantly increased (red) or decreased (green) contents.

Supplementary Table 6. Contents of soluble amino acids in leaves of wild-type and mutant plants.

A 4 h in the dark (absolute)

Amino acid	Ws-2	*xpt-1*	*tpt-1*	*tpt-1/xpt-1*	Col-0	*tpt-2*	*tpt-2/xpt-1*	amiRNA: *XPT tpt-2 #3*	amiRNA: *XPT tpt-2 #4*	*adg1-1/tpt-2*
					μmol·g^{-1} dw					
**Glu**	10.76 ± 0.60	12.24 ± 1.10	12.19 ± 0.50	20.67 ± 1.42	7.97 ± 0.80	9.89 ± 1.14	14.66 ± 1.13	9.65 ± 0.81	7.21 ± 0.66	12.96 ± 1.18
**Gln**	108.17 ± 9.86	102.51 ± 3.65	73.66 ± 3.79	57.88 ± 1.59	103.12 ± 4.23	57.51 ± 4.34	71.25 ± 7.05	59.07 ± 3.88	48.60 ± 4.83	35.18 ± 2.13
**Asp**	2.06 ± 0.14	2.64 ± 0.37	1.94 ± 0.12	4.34 ± 0.38	1.45 ± 0.10	1.31 ± 0.12	2.13 ± 0.29	1.56 ± 0.19	1.01 ± 0.09	3.54 ± 0.28
**Asn**	14.15 ± 1.60	14.27 ± 0.92	8.03 ± 0.71	9.18 ± 0.20	5.99 ± 0.41	4.58 ± 0.53	8.20 ± 6.43	5.08 ± 0.34	3.89 ± 0.42	33.68 ± 1.98
**Ala**	13.12 ± 1.91	12.63 ± 1.36	8.42 ± 0.22	8.74 ± 0.38	5.11 ± 0.60	5.87 ± 0.39	23.90 ± 4.56	5.94 ± 0.39	5.54 ± 0.37	4.86 ± 0.72
**Ser**	18.71 ± 2.05	29.43 ± 2.04	13.20 ± 0.50	16.33 ± 1.04	29.80 ± 1.52	15.34 ± 3.47	19.50 ± 2.10	10.72 ± 1.14	9.23 ± 0.84	11.14 ± 0.89
**Gly**	22.77 ± 10.92	32.30 ± 6.60	1.26 ± 0.14	1.20 ± 0.09	3.43 ± 1.30	1.33 ± 0.17	1.67 ± 0.14	0.95 ± 0.13	0.72 ± 0.06	1.05 ± 0.05
**Thr**	2.81 ± 0.16	3.76 ± 0.58	2.00 ± 0.19	4.91 ± 0.31	4.15 ± 0.54	3.36 ± 0.23	6.53 ± 0.68	3.02 ± 0.15	2.68 ± 0.28	7.74 ± 0.83
**His**	1.39 ± 0.18	1.32 ± 0.15	0.85 ± 0.04	0.91 ± 0.06	0.87 ± 0.10	0.72 ± 0.05	0.97 ± 0.06	0.76 ± 0.06	0.60 ± 0.05	1.29 ± 0.03
**Arg**	2.10 ± 0.30	1.80 ± 0.23	0.78 ± 0.10	1.30 ± 0.12	11.40 ± 2.12	1.95 ± 0.39	3.79 ± 1.13	1.07 ± 0.13	1.01 ± 0.12	7.60 ± 0.53
**Phe**	0.79 ± 0.08	0.75 ± 0.06	0.83 ± 0.04	0.92 ± 0.06	0.48 ± 0.03	0.53 ± 0.04	1.06 ± 0.05	0.62 ± 0.04	0.48 ± 0.06	0.96 ± 0.06
**Tyr**	0.40 ± 0.03	0.38 ± 0.05	0.48 ± 0.02	0.62 ± 0.05	0.27 ± 0.02	0.33 ± 0.02	0.53 ± 0.05	0.36 ± 0.04	0.25 ± 0.02	0.76 ± 0.04
**Trp**	0.37 ± 0.03	0.38 ± 0.04	0.47 ± 0.03	0.55 ± 0.04	0.28 ± 0.02	0.36 ± 0.02	0.56 ± 0.04	0.41 ± 0.04	0.28 ± 0.03	0.68 ± 0.05
**Val**	1.98 ± 0.09	1.82 ± 0.18	1.93 ± 0.07	1.95 ± 0.09	1.31 ± 0.03	1.32 ± 0.06	2.41 ± 0.21	1.46 ± 0.07	1.26 ± 0.09	3.07 ± 0.13
**Leu**	0.50 ± 0.02	0.49 ± 0.06	0.64 ± 0.03	0.67 ± 0.04	0.79 ± 0.02	0.85 ± 0.09	1.79 ± 0.11	1.10 ± 0.04	0.90 ± 0.10	2.09 ± 0.07
**Ile**	0.52 ± 0.04	0.51 ± 0.06	0.58 ± 0.04	0.75 ± 0.05	0.46 ± 0.02	0.38 ± 0.01	0.76 ± 0.15	0.42 ± 0.03	0.32 ± 0.03	1.29 ± 0.02
**Lys**	0.47 ± 0.04	0.45 ± 0.06	0.59 ± 0.04	0.71 ± 0.06	0.73 ± 0.19	0.43 ± 0.02	0.75 ± 0.21	0.48 ± 0.06	0.33 ± 0.03	1.53 ± 0.20
∑	201.09 ± 25.17	217.68 ± 15.05	127.86 ± 5.95	131.62 ± 3.07	177.62 ± 4.81	106.04 ± 5.25	160.45 ± 7.34	102.67 ± 6.61	84.32 ± 7.41	129.42 ± 6.44

Supplementary Table 6 (continued)

B 8 h in the light (absolute**)**

Amino acid	Ws-2	*xpt-1*	*tpt-1*	*tpt-1/xpt-1*	Col-0	*tpt-2*	*tpt-2/xpt-1*	amiRNA: *XPT tpt-2* #3	amiRNA: *XPT tpt-2* #4	*adg1-1/tpt-2*
					µmol·g^{-1} dw					
**Glu**	14.60 ± 2.03	18.11 ± 1.10	23.15 ± 2.03	21.85 ± 2.77	20.87 ± 2.53	20.64 ± 1.94	22.17 ± 2.73	18.38 ± 0.93	18.69 ± 1.89	21.37 ± 1.71
**Gln**	70.81 ± 7.17	71.23 ± 4.82	61.56 ± 3.88	39.13 ± 6.44	96.96 ± 1.22	45.68 ± 2.80	50.92 ± 4.92	53.64 ± 4.63	53.36 ± 4.43	22.15 ± 2.63
**Asp**	2.33 ± 0.42	3.82 ± 0.60	4.32 ± 0.44	6.03 ± 0.77	1.43 ± 0.39	2.08 ± 0.26	4.15 ± 0.60	2.60 ± 0.20	2.69 ± 0.33	3.20 ± 0.40
**Asn**	11.49 ± 1.41	12.09 ± 0.61	12.14 ± 0.70	10.05 ± 1.42	7.58 ± 1.32	5.92 ± 0.87	8.38 ± 0.54	7.22 ± 0.46	7.08 ± 0.62	32.30 ± 3.18
**Ala**	13.52 ± 0.82	14.49 ± 0.59	10.77 ± 0.41	10.53 ± 2.59	4.78 ± 1.67	4.72 ± 0.49	27.89 ± 5.01	9.23 ± 0.70	8.89 ± 0.77	13.85 ± 0.90
**Ser**	18.80 ± 1.81	22.90 ± 1.20	35.05 ± 2.37	28.07 ± 2.89	49.04 ± 4.81	48.29 ± 5.99	24.75 ± 1.17	26.59 ± 0.79	24.76 ± 1.68	20.72 ± 1.98
**Gly**	36.81 ± 2.81	41.94 ± 2.70	20.31 ± 2.13	5.85 ± 1.15	17.76 ± 5.03	15.40 ± 0.90	5.13 ± 0.30	8.48 ± 1.17	8.81 ± 1.12	2.64 ± 0.27
**Thr**	3.15 ± 0.24	4.55 ± 0.19	4.61 ± 0.21	5.84 ± 0.45	5.81 ± 0.40	7.26 ± 0.52	7.23 ± 0.31	6.33 ± 0.28	5.98 ± 0.31	9.05 ± 1.02
**His**	0.71 ± 0.18	0.99 ± 0.33	1.03 ± 0.17	0.78 ± 0.10	1.38 ± 0.20	1.21 ± 0.10	1.22 ± 0.06	1.17 ± 0.06	1.15 ± 0.09	1.29 ± 0.05
**Arg**	2.37 ± 0.30	1.77 ± 0.32	1.80 ± 0.15	1.89 ± 0.34	7.54 ± 2.61	3.63 ± 0.89	4.79 ± 0.87	2.80 ± 0.15	2.56 ± 0.14	13.21 ± 2.88
**Phe**	0.76 ± 0.06	0.83 ± 0.06	1.07 ± 0.10	0.95 ± 0.08	0.61 ± 0.14	0.81 ± 0.08	1.16 ± 0.04	0.87 ± 0.04	0.85 ± 0.08	0.88 ± 0.07
**Tyr**	0.41 ± 0.05	0.43 ± 0.04	0.75 ± 0.09	0.65 ± 0.06	0.37 ± 0.03	0.45 ± 0.03	0.62 ± 0.04	0.44 ± 0.03	0.42 ± 0.04	0.70 ± 0.02
**Trp**	0.35 ± 0.04	0.38 ± 0.03	0.64 ± 0.08	0.63 ± 0.08	0.50 ± 0.14	0.45 ± 0.04	0.63 ± 0.04	0.46 ± 0.03	0.44 ± 0.05	0.64 ± 0.07
**Val**	1.42 ± 0.10	1.69 ± 0.06	2.06 ± 0.13	1.80 ± 0.18	1.05 ± 0.26	1.50 ± 0.11	2.64 ± 0.18	1.72 ± 0.09	1.70 ± 0.12	2.32 ± 0.14
**Leu**	0.43 ± 0.05	0.48 ± 0.03	0.70 ± 0.07	0.61 ± 0.05	0.99 ± 0.17	0.93 ± 0.09	2.00 ± 0.14	1.06 ± 0.07	1.02 ± 0.07	1.21 ± 0.09
**Ile**	0.61 ± 0.07	0.67 ± 0.05	1.04 ± 0.10	0.93 ± 0.07	0.61 ± 0.07	0.70 ± 0.04	0.97 ± 0.04	0.68 ± 0.02	0.66 ± 0.04	0.87 ± 0.06
**Lys**	0.50 ± 0.06	0.52 ± 0.05	0.92 ± 0.10	0.83 ± 0.07	0.60 ± 0.13	0.59 ± 0.05	0.88 ± 0.06	0.62 ± 0.04	0.54 ± 0.03	1.55 ± 0.05
$\sum$	179.08 ± 15.03	196.88 ± 8.64	181.92 ± 10.81	136.42 ± 17.13	217.78 ± 8.17	160.26 ± 9.83	165.52 ± 11.62	142.31 ± 8.57	139.61 ± 11.14	147.94 ± 12.55

Supplementary Table 6 (continued)

C 4 h in the dark (relative)

Amino acid	Ws-2	*xpt-1*	*tpt-1*	*tpt-1/xpt-1*	Col-0	*tpt-2*	*tpt-2/xpt-1*	amiRNA: *XPT tpt-2* #3	amiRNA: *XPT tpt-2* #4	*adg1-1/tpt-2*
					mol·% of Σ					
Glu	5.35 ± 0.30	5.62 ± 0.50	9.53 ± 0.39	15.70 ± 1.08	4.49 ± 0.45	9.33 ± 1.08	9.14 ± 0.71	9.40 ± 0.78	8.56 ± 0.78	10.01 ± 0.92
Gln	53.79 ± 4.91	47.09 ± 1.68	57.61 ± 2.96	43.97 ± .21	58.06 ± 2.38	54.23 ± 4.09	44.40 ± 4.39	57.54 ± 3.77	57.64 ± 5.73	27.18 ± 1.65
Asp	1.03 ± 0.07	1.21 ± 0.17	1.52 ± 0.10	3.30 ± 0.29	0.81 ± 0.06	1.24 ± 0.12	1.33 ± 0.18	1.52 ± 0.19	1.20 ± 0.11	2.73 ± 0.22
Asn	7.04 ± 0.79	6.55 ± 0.42	6.28 ± 0.56	6.98 ± 0.16	3.37 ± 0.23	4.32 ± 0.50	5.11 ± 4.01	4.94 ± 0.33	4.61 ± 0.50	26.03 ± 1.53
Ala	6.53 ± 0.95	5.80 ± 0.63	6.58 ± 0.17	6.64 ± 0.29	2.88 ± 0.34	5.54 ± 0.36	14.89 ± 2.84	5.78 ± 0.38	6.57 ± 0.44	3.76 ± 0.56
Ser	9.30 ± 1.02	13.52 ± 0.94	10.33 ± 0.39	12.41 ± 0.79	16.78 ± 0.86	14.46 ± 3.27	12.15 ± 1.31	10.44 ± 1.11	10.95 ± 1.00	8.61 ± 0.69
Gly	11.32 ± 5.43	14.84 ± 3.03	0.99 ± 0.11	0.91 ± 0.07	1.93 ± 0.73	1.25 ± 0.16	1.04 ± 0.09	0.93 ± 0.13	0.85 ± 0.07	0.81 ± 0.04
Thr	1.40 ± 0.08	1.73 ± 0.26	1.57 ± 0.15	3.73 ± 0.24	2.34 ± 0.30	3.16 ± 0.21	4.07 ± 0.42	2.94 ± 0.15	3.18 ± 0.33	5.98 ± 0.64
His	0.69 ± 0.09	0.61 ± 0.07	0.67 ± 0.03	0.69 ± 0.04	0.49 ± 0.06	0.68 ± 0.05	0.60 ± 0.04	0.74 ± 0.06	0.71 ± 0.06	0.99 ± 0.03
Arg	1.05 ± 0.15	0.83 ± 0.10	0.61 ± 0.07	0.99 ± 0.09	6.42 ± 1.19	1.84 ± 0.36	2.36 ± 0.71	1.04 ± 0.13	1.20 ± 0.14	5.87 ± 0.41
Phe	0.39 ± 0.04	0.35 ± 0.03	0.65 ± 0.03	0.70 ± 0.05	0.27 ± 0.02	0.50 ± 0.03	0.66 ± 0.03	0.61 ± 0.04	0.58 ± 0.07	0.74 ± 0.05
Tyr	0.20 ± 0.02	0.17 ± 0.02	0.38 ± 0.02	0.47 ± 0.04	0.15 ± 0.01	0.31 ± 0.02	0.33 ± 0.03	0.35 ± 0.03	0.29 ± 0.03	0.59 ± 0.03
Trp	0.19 ± 0.01	0.18 ± 0.02	0.36 ± 0.02	0.42 ± 0.03	0.16 ± 0.01	0.34 ± 0.02	0.35 ± 0.02	0.40 ± 0.04	0.33 ± 0.03	0.52 ± 0.04
Val	0.98 ± 0.04	0.84 ± 0.08	1.51 ± 0.06	1.48 ± 0.07	0.74 ± 0.02	1.25 ± 0.05	1.50 ± 0.13	1.42 ± 0.07	1.49 ± 0.11	2.37 ± 0.10
Leu	0.25 ± 0.01	0.23 ± 0.03	0.50 ± 0.02	0.51 ± 0.03	0.45 ± 0.01	0.80 ± 0.08	1.12 ± 0.07	1.07 ± 0.04	1.07 ± 0.12	1.61 ± 0.05
Ile	0.26 ± 0.02	0.24 ± 0.03	0.46 ± 0.03	0.57 ± 0.04	0.26 ± 0.01	0.35 ± 0.01	0.48 ± 0.09	0.41 ± 0.03	0.37 ± 0.03	1.00 ± 0.02
Lys	0.24 ± 0.02	0.20 ± 0.03	0.46 ± 0.03	0.54 ± 0.04	0.41 ± 0.11	0.40 ± 0.02	0.47 ± 0.13	0.47 ± 0.05	0.39 ± 0.04	1.18 ± 0.15
Σ	100.00 ± 12.52	100.00 ± 6.91	100.00 ± 4.65	100.00 ± 2.34	100.00 ± 2.71	100.00 ± 4.95	100.00 ± 4.57	100.00 ± 6.44	100.00 ± 8.79	100.00 ± 4.97

Supplementary Table 6 (continued)

D 8 h in the light (relative)

Amino acid	Ws-2	*xpt-1*	*tpt-1*	*tpt-1/xpt-1*	Col-0	*tpt-2*	*tpt-2/xpt-1*	amiRNA: *XPT tpt-2 #3*	amiRNA: *XPT tpt-2 #4*	*adg1-1/tpt-2*
	mol·% of ∑									
**Glu**	8.15 ± 1.13	9.20 ± 0.56	12.73 ± 1.12	16.02 ± 2.03	9.58 ± 16	12.88 ± 1.21	13.39 ± 1.65	12.92 ± 0.66	13.39 ± 1.35	14.45 ± 1.16
**Gln**	39.54 ± 4.00	36.18 ± 45	33.84 ± 2.13	28.68 ± 4.72	44.52 ± 0.56	28.50 ± 1.75	30.77 ± 2.97	37.70 ± 3.26	38.22 ± 3.17	14.97 ± 1.78
**Asp**	1.30 ± 0.23	1.94 ± 0.30	2.38 ± 0.24	4.42 ± 0.57	0.66 ± 0.18	1.30 ± 0.16	2.51 ± 0.36	1.82 ± 0.14	1.93 ± 0.24	2.16 ± 0.27
**Asn**	6.42 ± 0.79	6.14 ± 0.31	6.67 ± 0.38	7.37 ± 1.04	3.48 ± 0.60	3.70 ± 0.54	5.07 ± 0.33	5.08 ± 0.32	5.07 ± 0.44	21.83 ± 2.15
**Ala**	7.55 ± 0.46	7.36 ± 0.30	5.92 ± 0.23	7.72 ± 1.90	2.20 ± 0.77	2.95 ± 0.30	16.85 ± 3.02	6.49 ± 0.50	6.37 ± 0.55	9.36 ± 0.61
**Ser**	10.50 ± 1.01	11.63 ± 0.61	19.27 ± 1.30	20.57 ± 2.12	22.52 ± 2.21	30.13 ± 3.74	14.95 ± 0.71	18.68 ± 0.56	17.74 ± 1.20	14.01 ± 1.34
**Gly**	20.56 ± 1.57	21.30 ± 1.37	11.16 ± 1.17	4.29 ± 0.84	8.15 ± 2.31	9.61 ± 0.56	3.10 ± 0.18	5.96 ± 0.82	6.31 ± 0.80	1.79 ± 0.19
**Thr**	1.76 ± 0.14	2.31 ± 0.10	2.54 ± 0.11	4.28 ± 0.33	2.67 ± 0.18	4.53 ± 0.32	4.37 ± 0.18	4.45 ± 0.19	4.29 ± 0.22	6.12 ± 0.69
**His**	0.40 ± 0.10	0.50 ± 0.17	0.56 ± 0.10	0.57 ± 0.07	0.63 ± 0.09	0.76 ± 0.06	0.74 ± 0.04	0.82 ± 0.04	0.83 ± 0.06	0.88 ± 0.03
**Arg**	1.32 ± 0.17	0.90 ± 0.16	0.99 ± 0.08	1.39 ± 0.25	3.46 ± 1.20	2.26 ± 0.56	2.89 ± 0.52	1.97 ± 0.11	1.83 ± 0.10	8.93 ± 1.95
**Phe**	0.43 ± 0.04	0.42 ± 0.03	0.59 ± 0.05	0.69 ± 0.06	0.28 ± 0.06	0.51 ± 0.05	0.70 ± 0.03	0.61 ± 0.03	0.61 ± 0.06	0.59 ± 0.05
**Tyr**	0.23 ± 0.03	0.22 ± 0.02	0.41 ± 0.05	0.48 ± 0.04	0.17 ± 02	0.28 ± 0.02	0.37 ± 0.02	0.31 ± 0.02	0.30 ± 0.03	0.47 ± 0.02
**Trp**	0.20 ± 0.02	0.19 ± 0.02	0.35 ± 0.04	0.46 ± 0.06	0.23 ± 0.06	0.28 ± 0.02	0.38 ± 0.03	0.33 ± 0.02	0.32 ± 0.03	0.43 ± 0.04
**Val**	0.79 ± 0.06	0.86 ± 0.03	1.14 ± 0.07	1.32 ± 0.13	0.48 ± 0.12	0.94 ± 0.07	1.59 ± 0.11	1.21 ± 0.07	1.21 ± 0.09	1.57 ± 0.09
**Leu**	0.24 ± 0.03	0.24 ± 0.01	0.38 ± 0.04	0.45 ± 0.04	0.45 ± 0.08	0.58 ± 0.05	1.21 ± 0.08	0.74 ± 0.05	0.73 ± 0.05	0.82 ± 0.06
**Ile**	0.34 ± 0.04	0.34 ± 0.03	0.57 ± 0.06	0.68 ± 0.05	0.28 ± 0.03	0.44 ± 0.02	0.59 ± 0.02	0.48 ± 0.02	0.47 ± 0.03	0.59 ± 0.04
**Lys**	0.28 ± 0.04	0.26 ± 0.02	0.51 ± 0.06	0.61 ± 0.05	0.27 ± 0.06	0.37 ± 0.03	0.53 ± 0.03	0.44 ± 0.03	0.39 ± 0.02	1.05 ± 0.03
∑	100.00 ± 8.39	100.00 ± 4.39	100.00 ± 5.94	100.00 ± 12.55	100.00 ± 3.75	100.00 ± 6.14	100.00 ± 7.02	100.00 ± 6.02	100.00 ± 7.98	100.00 ± 8.48

The plants were grown in soil under HL-conditions (PDF = 300 $\mu mol \cdot m^{-2} \cdot s^{-1}$) in the long-day. Samples were taken either 4h in the dark (**A**, **C**) or 8h in the light (**B**, **D**). The absolute values are shown in (**A**) and (**C**), and the relative values (expressed as percent of ∑ amino acids) in (**D**) and (**E**). The data represents the mean ± SE of n = 4 to 5 independent replicates.
