# Supplementary material for: The Combined Loss of Triose Phosphate and Xylulose 5-Phosphate/Phosphate Translocators Leads to Severe Growth Retardation and Impaired Photosynthesis in Arabidopsis thaliana tpt/xpt Double Mutants
Source: Front Plant Sci. 2018 Oct 2;9:1331. doi: 10.3389/fpls.2018.01331 (PMC6175978; doi:10.3389/fpls.2018.01331)
Supplement: DATA SHEET S3 — Contains Supplementary Document 1, which describes the phenotypic analysis of xpt-1[2] single mutants and amiRNA:XPT lines in the Ws-2 or Col-0 background. It comprises 4 Figures and 5 Tables including 1 Table with a statistical analysis. [file Data_Sheet_3.pdf]

Supplementary Document 1

Loss of function mutants of the XPT lack any pronounced phenotype in *Arabidopsis thaliana*

Two mutant alleles of the *XPT* (*xpt-1* and *xpt-2*) as well as amiRNA:*XPT* lines in the Ws-2 and Col-0 backgrounds were analyzed in some detail for phenotypic differences compared to the respective wild-type lines (see main article, Figure 1).

1. Vegetative development

In Doc S1, Figure 1 examples of the individual lines are shown both in the cotyledon stage and 21 days after sowing. Apart from slightly larger cotyledons in the line *xpt-1* (see also Doc S1, Figure 4), there were no obvious phenotypic differences between the lines that could be attributed to the lesion in the XPT. This observation was supported by a growth analysis comprising four time points within 21 days after sowing, starting from the emergence of the first true leaves (Doc S1, Figure 2). Small differences that occurred were due to the different accessions, i.e. Ws-2, Col-0, or Col-3 rather than the impaired XPT in the individual backgrounds. Likewise growth rates of roots were not significantly affected by the absence or diminished XPT function compared to the wild-type plants (Doc S1, Table 1).

Leaf contents of Chl, carotenoids, or proteins lacked any significant differences between wild-type, mutant or amiRNA*:XPT* plants. Similarly, neither Chl *a/b*-ratios or specific fresh weights were differently affected in the individual lines (Doc S1, Table 2), suggesting that neither the photosynthetic machinery nor water contents of the leaves seemed to be influenced by the impaired XPT.

Photosynthetic characteristics determined by Imaging PAM fluorometry comprise information on the Chl *a* fluorescence yield of dark-adapted plants, i.e. F_v/F_m-ratios, and the derived relative ETR at a PFD similar to the growth light or at a saturating PFD, i.e. relative ETR_{700}. Although differences in certain parameters like F_v/F_m-ratios or ETR_{700} were rather small between the individual lines, some of these differences tended to be significant due to a low SE combined with a high n (i.e. n = 9) (Doc S1, Table 3)

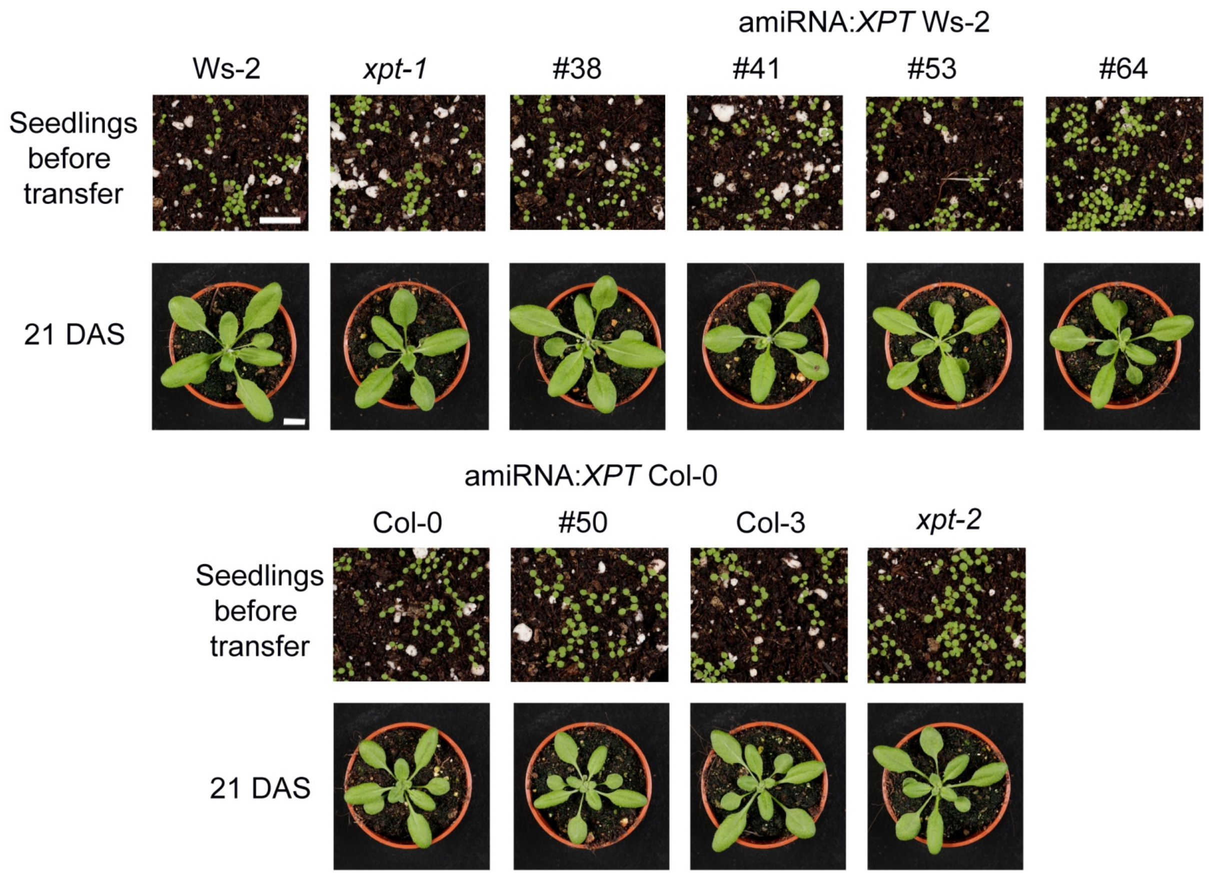

Doc S1, Figure 1. Phenotype of seedlings and leaf rosettes of plants impaired in the XPT compared to the wild type.
Mutants with lesions in the XPT (*xpt-1*, *xpt-2*), amiRNA*:XPT* lines and wild-type plants (Ws-2, Col-0, Col-3) were grown on soil in a temperature controlled growth cabinet under SL-conditions (i.e. a PFD of 150 $\mu mol \cdot ^{-2} \cdot s^{-1}$) in the long-day. The upper rows of each of the two panels represent seedlings at the cotyledon state shortly before transfer of the plants to single pots. The lower rows show examples of rosette phenotypes of the lines after 21 days of sowing (DAS). The size bars represent 1 cm.

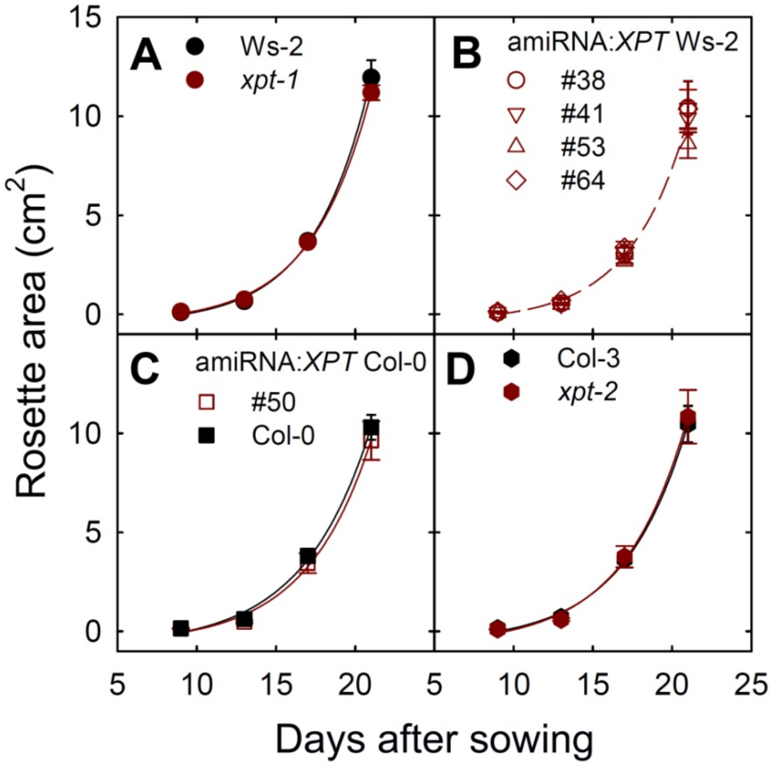

Doc S1, Figure 2. Time dependent increase in rosette areas of mutant and transgenic plants impaired in the XPT compared to the wild type.
Mutants with lesions in the XPT (*xpt-1*, *xpt-2*), amiRNA:*XPT* lines and wild-type plants (Ws-2, Col-0, Col-3) were grown in soil in a temperature controlled growth cabinet under SL-conditions (i.e. a PFD of 150 µmol·$^{-2}$·s^{-1}) in the long-day. In (**A**) the development of rosette areas of *xpt-1* and its respective wild type is shown and can be compared with those of amiRNA:*XPT* lines in the Ws-2 background (**B**). In (**C**) and (**D**) comparisons of rosette area developments are displayed for Col-0 versus an amiRNA:*XPT* line and for Col-3 versus *xpt-2,* respectively. The data represent the mean ± SE of n = 5 to 6 replicates. A statistical analysis of the data is contained in Doc S1, Table 5A.

2. Generative growth

In contrast to the vegetative growth characteristics, generative growth revealed significant changes in seed size (represented by the seed area [within the Ws-2 accession]) and weight (in all accessions) only in the line *xpt-1*, but not in any other line with an impaired XPT in the same accession (i.e. amiRNA:*XPT* Ws-2 lines) or in different accession like Col-0 (i.e. amiRNA:*XPT* Col-0) or Col-3 (i.e. *xpt-2*) (Doc S1, Table 4). Interestingly, in *xpt-1* the increase in seed weight was accompanied by shorter siliques that carried a smaller number of seeds per silique. Although the total number of siliques per plant was also increased in *xpt-1* (Doc S1, Table 4) the total seed weight per plant was not different compared to Ws-2 (i.e. an increase by a factor of 1.07).

As might be expected, the increased seed size of *xpt-1* was accompanied by higher contents of fatty acid and proteins per seed (Doc S1, Table 4). However, neither the composition of

fatty acids nor of proteins was significantly affected by the increase in their total amounts (Doc S1, Figure 3). Probably larger seeds give rise to slightly larger cotyledons in this line compared to its wild type (Doc S1, Figure 4)

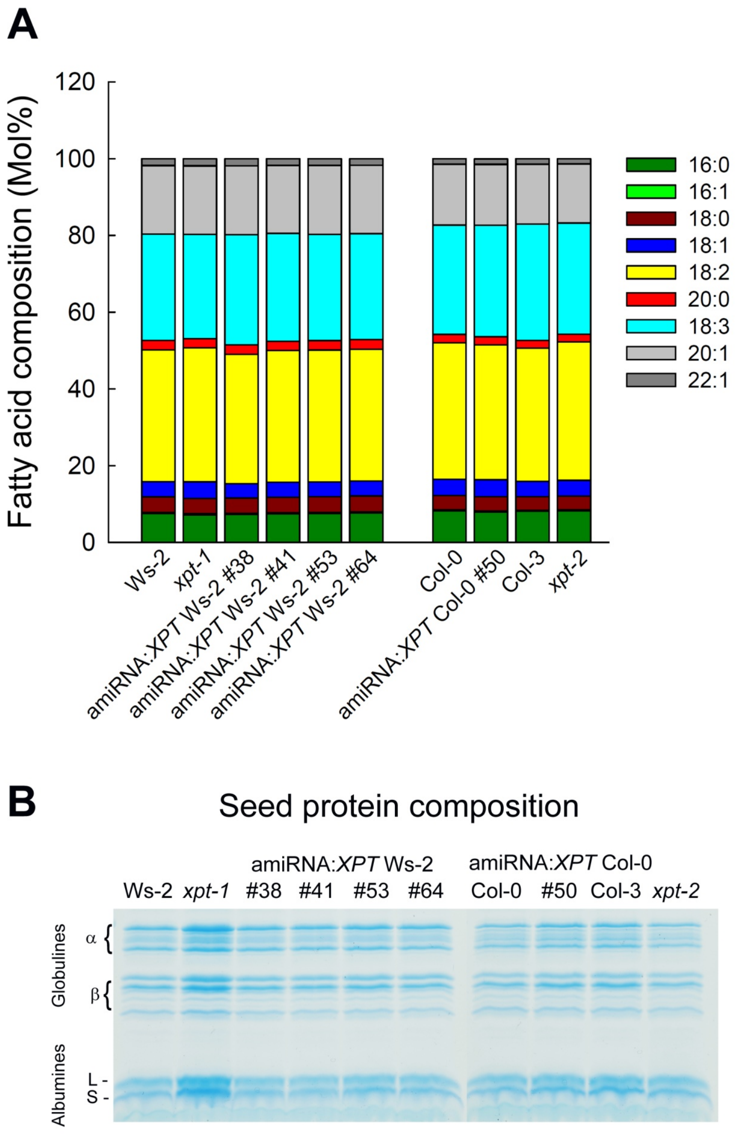

Doc S1, Figure 3. Composition of fatty acids and proteins in seeds of plants impaired in the XPT function.
(**A**) Relative distribution of individual fatty acid classes in XPT mutants (*xpt-1*, *xpt-2*), amiRNA:*XPT* lines and the respective wild-type plants (Ws-2, Col-0, and Col-3). For the sake of clarity error bars of n=5 replicates have been omitted. A statistical analysis of the data (one way ANOVA/Tuckey-Kramer) is contained in Doc S1, Table 5D. (**B**) Size distribution of seed proteins after separation on SDS-PAGE. The major storage protein, α and β globulines and L- and S-albumines are indicated.

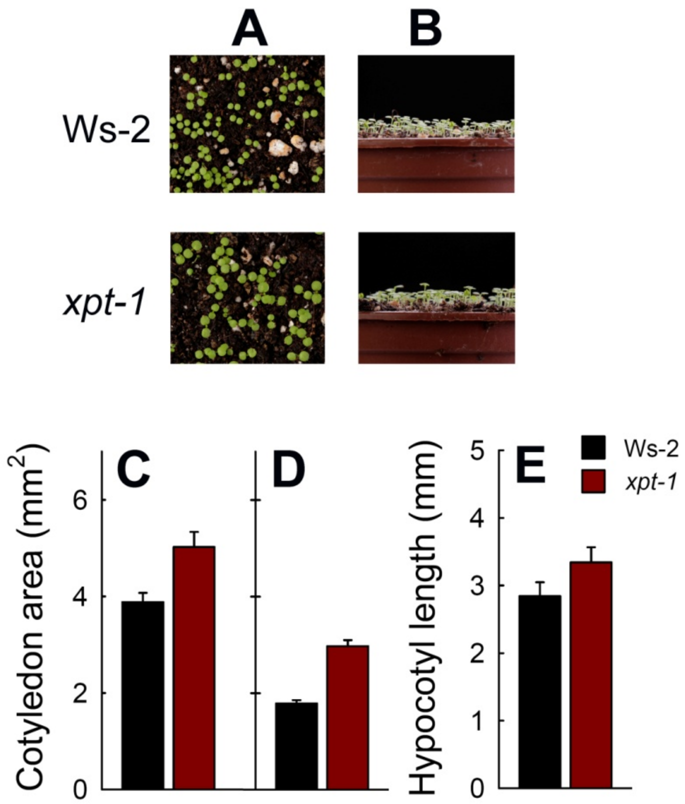

Doc S1, Figure 4. Seedling phenotype of the *xpt-1* mutant compared to the wild type.
(**A**) and (**B**) depict seedlings grown on soil under LL-conditions (30 µmol·$^{-2}$·s^{-1}) either from the top (cotyledon areas) or from the side (hypocotyl length). From such images the average cotyledon area has been calculated for seedling grown under SL- (**C**) or LL-conditions (**D**). The average hypocotyl length has been calculated for LL-conditions only (**E**).

3. Concluding remarks

A lesion in the XPT had no obvious effect on development and physiological performance of three different accessions of *A. thaliana* using both knockout mutants or amiRNA approaches. Moreover, apart from the line *xpt-1* generative parameters were also not significantly affected by a lesion in the XPT. It is likely that the impaired seed development is specific for the particular line and is not a characteristic of the accession Ws-2 as amiRNA:*XPT* Ws-2 plants lacked any such changes.

Doc S1, Table 1. Root growth of mutant and transgenic plants impaired in the XPT compared to the respective wild-type plants.

Plant line	Root length (cm)			Growth rate ($cm \cdot d^{-1}$)
	6 DAS	12 DAS	Δ	
Ws-2	0.77 ± 0.04	4.79 ± 0.20	4.02 ± 0.20	0.67 ± 0.03
xpt-1	1.04 ± 0.03	5.34 ± 0.28	4.31 ± 0.27	0.72 ± 0.05
amiRNA:*XPT* Ws-2 #38	0.73 ± 0.04	4.68 ± 0.29	3.95 ± 0.26	0.66 ± 0.04
amiRNA:*XPT* Ws-2 #41	0.88 ± 0.04	4.82 ± 0.33	3.94 ± 0.31	0.66 ± 0.05
amiRNA:*XPT* Ws-2 #53	0.81 ± 0.04	4.69 ± 0.27	3.88 ± 0.24	0.65 ± 0.04
amiRNA:*XPT* Ws-2 #64	0.98 ± 0.02	4.98 ± 0.27	4.00 ± 0.27	0.67 ± 0.05
Col-0	0.89 ± 0.04	4.89 ± 0.28	4.01 ± 0.27	0.67 ± 0.05
amiRNA:*XPT* Col-0 #50	1.07 ± 0.05	5.34 ± 0.49	4.27 ± 0.45	0.71 ± 0.08
Col-3	0.70 ± 0.06	4.65 ± 0.39	3.96 ± 0.35	0.66 ± 0.06
xpt-2	0.76 ± 0.05	4.54 ± 0.45	3.78 ± 0.41	0.63 ± 0.07

Mutants with lesions in the XPT (*xpt-1*, *xpt-2*), amiRNA:*XPT* lines and wild-type plants (Ws-2, Col-0, Col-3) were grown vertically on ½ strength MS agar plates in a temperature controlled growth cabinet under SL conditions (i.e. a PFD of 150 $\mu mol \cdot m^{-2} \cdot s^{-1}$) in the long-day. The root length was measured at days 6 and 12 after sowing (DAS) and the Δ-values for each root calculated. From the Δ-values the average growth rates were calculated. The data represent the mean ± SE of n=12 replicates. A statistical analysis of the data is contained in Doc S1, Table 5A.

Doc S1, Table 2. Pigment and protein content as well as Chl *a/b*-ratios and specific fresh weights of rosette leaves of mutant and transgenic plants impaired in the XPT compared to the respective wild-type plants.

Plant line	Chl content	Carotenoid content	Chl *a/b*-ratio	Protein content	Specific fw
	(mg·m^{-2})			(g·m^{-2})	
Ws-2	183.37 ± 6.47	30.94 ± 0.50	3.17 ± 0.12	2.97 ± 0.16	162.91 ± 3.46
xpt-1	189.26 ± 1.84	30.25 ± 0.61	3.35 ± 0.09	2.70 ± 0.15	160.55 ± 3.77
amiRNA:*XPT* Ws-2 #38	185.93 ± 12.29	31.10 ± 1.60	3.28 ± 0.18	3.22 ± 0.67	161.15 ± 25.96
amiRNA:*XPT* Ws-2 #41	190.13 ± 8.74	32.16 ± 0.63	3.29 ± 0.10	4.45 ± 0.70	200.92 ± 21.33
amiRNA:*XPT* Ws-2 #53	179.30 ± 6.72	32.32 ± 1.35	3.69 ± 0.10	4.20 ± 0.80	159.77 ± 23.83
amiRNA:*XPT* Ws-2 #64	180.28 ± 4.04	29.30 ± 1.03	3.14 ± 0.13	3.25 ± 0.23	119.42 ± 4.55
Col-0	176.93 ± 2.42	27.31 ± 1.59	3.53 ± 0.20	2.48 ± 0.18	151.30 ± 2.98
amiRNA:*XPT* Col-0 #50	182.35 ± 2.48	28.92 ± 1.31	3.42 ± 0.14	2.96 ± 0.21	158.93 ± 4.73
Col-3	184.22 ± 3.04	29.45 ± 1.18	3.38 ± 0.04	2.80 ± 0.17	160.59 ± 5.86
xpt-2	183.69 ± 6.47	30.24 ± 0.99	3.43 ± 0.12	2.41 ± 0.11	163.72 ± 5.10

Mutants with lesions in the XPT (*xpt-1*, *xpt-2*), amiRNA:*XPT* lines and wild-type plants (Ws-2, Col-0, Col-3) were grown on soil in a temperature controlled growth cabinet under SL-conditions (i.e. a PFD of 150 µmol·m^{-2}·s^{-1}) in the long-day for 21 days after sowing. The leaves of the individual lines were harvested at the middle of the light period. The data represent the mean ± SE of n = 5 replicates with the exception of the specific fw with 10 replicates. A statistical analysis of the data is contained in Doc S1, Table 5B.

Doc S1, Table 3. Photosynthesis parameters derived from Chl *a* fluorescence determined by an Imaging PAM fluorometer with rosette leaves of mutant and transgenic plants impaired in the XPT compared to the respective wild-type plants.

Plant line	F_v/F_m-ratio	Relative $ETR_{(146)}$	Relative $ETR_{(700)}$
Ws-2	0.770 ± 0.002	24.5 ± 0.2	65.1 ± 1.3
xpt-1	0.754 ± 0.003	23.8 ± 0.3	66.9 ± 2.1
amiRNA:*XPT* Ws-2 #38	0.749 ± 0.000	24.4 ± 0.5	61.7 ± 2.4
amiRNA:*XPT* Ws-2 #41	0.760 ± 0.002	24.4 ± 0.4	63.7 ± 1.9
amiRNA:*XPT* Ws-2 #53	0.758 ± 0.002	23.7 ± 0.4	64.5 ± 1.5
amiRNA:*XPT* Ws-2 #64	0.762 ± 0.003	24.7 ± 0.3	74.7 ± 2.2
Col-0	0.767 ± 0.001	25.6 ± 0.4	69.8 ± 1.8
amiRNA:*XPT* Col-0 #50	0.771 ± 0.002	26.9 ± 0.2	69.8 ± 1.8
Col-3	0.775 ± 0.001	27.9 ± 0.2	74.0 ± 1.8
xpt-2	0.765 ± 0.002	27.5 ± 0.3	74.1 ± 2.2

Mutants with lesions in the XPT (*xpt-1*, *xpt-2*), amiRNA:*XPT* lines and wild-type plants (Ws-2, Col-0, Col-3) were grown in soil in a temperature controlled growth cabinet under SL-conditions (i.e. a PFD of 150 $\mu mol \cdot^{-2} \cdot s^{-1}$) in the long-day for 21 days after sowing. After dark adaptation of the plants for about 30 min, photosynthesis was induced with an actinic light of 146 $\mu mol \cdot m^{-2} \cdot s^{-1}$ for 5 min. Immediately after the induction of photosynthesis a light dependency of relative ETR was determined ending at the highest PFD of 700 $\mu mol \cdot m^{-2} \cdot s^{-1}$. The data represent the mean ± SE of n = 9 replicates. A statistical analysis of the data is contained in Doc S1, Table 5B.

Doc S1, Table 4. Generative growth parameters including contents of fatty acids and protein in seeds of mutant and transgenic plants impaired in the XPT compared to the respective wild-type plants.

Plant line	Silique length (mm)	Seeds per silique	Siliques per plant	Seed area (mm²)	Seed weight	Seed FA (µg per seed)	Seed protein
Ws-2	14.70 ± 0.32	59.07 ± 2.96	503.2 ± 25.5	0.1048 ± 0.0005	19.13 ± 0.47	9.66 ± 0.39	1.57 ± 0.14
xpt-1	13.30 ± 0.31	43.73 ± 2.96	555.0 ± 51.7	0.1104 ± 0.0009	25.10 ± 0.37	11.67 ±0.37	2.46 ± 0.09
amiRNA:*XPT* Ws-2 #38	13.87 ± 0.39	59.00 ± 2.68	487.4 ± 32.7	0.1006 ± 0.0005	18.30 ± 0.04	9.10 ± 0.43	1.43 ± 0.14
amiRNA:*XPT* Ws-2 #41	14.33 ± 0.32	62.40 ± 2.29	472.8 ± 31.7	0.1007 ± 0.0005	18.15 ± 0.42	8.81 ±0.22	1.35 ± 0.06
amiRNA:*XPT* Ws-2 #53	14.50 ± 0.36	61.00 ± 2.96	499.4 ± 31.4	0.1017 ± 0.0005	19.00 ± 0.21	9.22 ± 0.76	1.43 ± 0.09
amiRNA:*XPT* Ws-2 #64	14.40 ± 0.32	61.27 ± 2.54	487.8 ± 24.4	0.0961 ± 0.0004	18.90 ± 0.25	8.77 ± 0.21	1.57 ± 0.05
Col-0	14.70 ± 0.24	61.47 ± 1.64	326.0 ± 17.1	0.1082 ± 0.0004	19.43 ± 0.72	9.53 ± 0.12	1.21 ± 0.05
amiRNA:*XPT* Col-0 #50	13.73 ± 0.43	48.67 ± 4.41	349.8 ± 23.8	0.1111 ± 0.0006	21.43 ± 0.42	9.74 ± 0.23	1.22 ± 0.15
Col-3	15.03 ± 0.25	59.80 ± 1.25	303.8 ± 23.9	0.1111 ± 0.0007	20.18 ± 0.31	8.83 ± 0.19	1.26 ± 0.13
xpt-2	14.80 ± 0.32	61.00 ± 1.74	286.0 ± 13.7	0.1098 ± 0.0005	19.03 ± 0.25	8.37 ± 0.29	1.27 ± 0.12

Mutants with lesions in the XPT (*xpt-1*, *xpt-2*), amiRNA:*XPT* lines and wild-type plants (Ws-2, Col-0, Col-3) were grown in soil in a temperature controlled growth cabinet under SL-conditions (i.e. a PFD of 150 µmol$\cdot^{-2}\cdot$s^{-1}) in the long day for 43 days after sowing. The data represent the mean ± SE. The number of replicates was n = 15 for \`silique length´ and \`seeds per silique´, n = 5 for \`siliques per plant´, n = 430 to 530 for \`seed area´, n = 4 for \`seed weight´ (i.e. batches of 100 seeds), and n = 5 for \`seed FA´ and \`seed protein´. A statistical analysis of the data is contained in Doc S1, Table 5C.

Doc S1, Table 5. Statistical analysis (ANOVA/Tukey-Kramer) of physiological growth parameters of wild-type, mutant, and transgenic plants defective in the XPT. The data in (**A**), (**B**), (**C**), and (**D**) are contained in Doc1, Figure 2 and Table 1; Doc1, Tables 2 and 3; Doc1, Table 4; and Doc1, Figure 3 and Table 4, respectively. The biotypes are denoted, ***a*** = Col-0, ***b*** = Ws-2, ***c*** = *xpt-1*, ***d*** = amiRNA:XPT Ws-2 #38, ***e*** = amiRNA:XPT Ws-2 #41, ***f*** = amiRNA:XPT Ws-2 #53, ***g*** = amiRNA:XPT Ws-2 #64, ***h*** = amiRNA:XPT Col-0 #50, ***i*** = Col-3, and ***j*** = *xpt-2.* The significance levels of P < 0.05 or P < 0.01 are indicated by light or dark blue colors.

A

7 Comparisons

**Vegetative growth**	***b vs a***	***c vs a***	***d vs a***	***e vs a***	***f vs a***	***g vs a***	***c vs b***	***d vs b***	***e vs b***	***f vs b***	***g vs b***	***d vs c***	***e vs c***	***f vs c***	***g vs c***	***e vs d***	***f vs d***	***g vs d***	***f vs e***	***g vs e***	***f vs g***
**Rosettes**																					
9 DAS																					
13 DAS																					
17 DAS																					
21 DAS																					
**Roots**																					
6 DAG																					
12 DAG																					
∆ root growth																					
Daily root growth																					

5 Comparisons

	b vs a	***h vs a***	***i vs a***	***j vs a***	***h vs b***	***i vs b***	***j vs b***	***i vs h***	***j vs h***	***i vs j***
**Rosettes**										
9 DAS										
13 DAS										
17 DAS										
21 DAS										
**Roots**										
6 DAG										
12 DAG										
∆ root growth										
Daily root growth										

Doc S1, Table 5 (continued)

B

7 Comparisons

**Physiological parameters**	***b vs a***	***c vs a***	***d vs a***	***e vs a***	***f vs a***	***g vs a***	***c vs b***	***d vs b***	***e vs b***	***f vs b***	***g vs b***	***d vs c***	***e vs c***	***f vs c***	***g vs c***	***e vs d***	***f vs d***	***g vs d***	***f vs e***	***g vs e***	***f vs g***
**Leaf constituents**																					
Chl content																					
Carotenoid content																					
Chl *a*/*b*-ratio																					
Protein content																					
Specific fw																					
**PS parameters**																					
F_v/F_m-ratio																					
Relative $ETR_{(146)}$																					
Relative $ETR_{(700)}$																					

5 Comparisons

**Physiological parameters**	***b vs a***	***h vs a***	***i vs a***	***j vs a***	***h vs b***	***i vs b***	***j vs b***	***i vs h***	***j vs h***	***i vs j***
**Leaf constituents**										
Chl content										
Carotenoid content										
Chl *a*/*b*-ratio										
Protein content										
Specific fw										
**PS parameters**										
F_v/F_m-ratio										
Relative $ETR_{(146)}$										
Relative $ETR_{(700)}$										

a = Col-0, ***b*** = Ws-2, ***c*** = *xpt-1*, ***d*** = amiRNA:XPT Ws-2 #38, ***e*** = amiRNA:XPT Ws-2 #41, ***f*** = amiRNA:XPT Ws-2 #53, ***g*** = amiRNA:XPT Ws-2 #64, ***h*** = amiRNA:XPT Col-0 #50, ***i*** = Col-3, and ***j*** = *xpt-2.*

Doc S1, Table 5 (continued)

C

7 Comparisons

Generative growth	*b vs a*	*c vs a*	*d vs a*	*e vs a*	*f vs a*	*g vs a*	*c vs b*	*d vs b*	*e vs b*	*f vs b*	*g vs b*	*d vs c*	*e vs c*	*f vs c*	*g vs c*	*e vs d*	*f vs d*	*g vs d*	*f vs e*	*g vs e*	*f vs g*
Silique length																					
Seeds per silique																					
Siliques per plant																					
Seed area																					
Seed weight																					

5 Comparisons

Generative growth	*b vs a*	*h vs a*	*i vs a*	*j vs a*	*h vs b*	*i vs b*	*j vs b*	*i vs h*	*j vs h*	*i vs j*
Silique length										
Seeds per silique										
Siliques per plant										
Seed area										
Seed weight										

a = Col-0, ***b*** = Ws-2, ***c*** = *xpt-1*, ***d*** = amiRNA:XPT Ws-2 #38, ***e*** = amiRNA:XPT Ws-2 #41, ***f*** = amiRNA:XPT Ws-2 #53, ***g*** = amiRNA:XPT Ws-2 #64, ***h*** = amiRNA:XPT Col-0 #50, ***i*** = Col-3, and ***j*** = *xpt-2.*

Doc S1, Table 5 (continued)

D

7 Comparisons

Seed parameters	*b vs a*	*c vs a*	*d vs a*	*e vs a*	*f vs a*	*g vs a*	*c vs b*	*d vs b*	*e vs b*	*f vs b*	*g vs b*	*d vs c*	*e vs c*	*f vs c*	*g vs c*	*e vs d*	*f vs d*	*g vs d*	*f vs e*	*g vs e*	*f vs g*
**Seed constituents**																					
Poteins																					
Fatty acids																					
**FA composition**																					
16:0																					
16:1																					
18:0																					
18:1																					
18:2																					
20:0																					
18:3																					
20:1																					
22:0																					
22:1																					

5 Comparisons

Seed parameters	*b vs a*	*h vs a*	*i vs a*	*j vs a*	*h vs b*	*i vs b*	*j vs b*	*i vs h*	*j vs h*	*i vs j*
**Seed constituents**										
Poteins										
Fatty acids										
**FA composition**										
16:0										
16:1										
18:0										
18:1										
18:2										
20:0										
18:3										
20:1										
22:0										
22:1										

a = Col-0, ***b*** = Ws-2, ***c*** = *xpt-1*, ***d*** = amiRNA:XPT Ws-2 #38, ***e*** = amiRNA:XPT Ws-2 #41, ***f*** = amiRNA:XPT Ws-2 #53, ***g*** = amiRNA:XPT Ws-2 #64, ***h*** = amiRNA:XPT Col-0 #50, ***i*** = Col-3, and ***j*** = *xpt-2.*
