# Supplementary material for: The Combined Loss of Triose Phosphate and Xylulose 5-Phosphate/Phosphate Translocators Leads to Severe Growth Retardation and Impaired Photosynthesis in Arabidopsis thaliana tpt/xpt Double Mutants
Source: Front Plant Sci. 2018 Oct 2;9:1331. doi: 10.3389/fpls.2018.01331 (PMC6175978; doi:10.3389/fpls.2018.01331)
Supplement: DATE SHEET S4 — Contains Supplementary Document 2 which comprises 13 Tables with statistical analyses of the data. [file Data_Sheet_4.pdf]

Supplementary Document 2

Statistical analyses

Doc S2 Table 1. Statistical analysis (ANOVA/Tukey-Kramer) of growth parameters of wild-type and mutant plants. Growth rates of leaf rosettes were determined for plants grown in soil under SL-conditions (PDF = 150 µmol·m^{-2}·s^{-1}) in the long-day (Figure 2C, main article). Root growth was assessed for plants grown on 1/2MS agar (Figure 2D, main article). For the generative growth parameters plants were grown as described in Table 1 (main article). The biotypes are denoted, ***a*** = Ws-2, ***b*** = *xpt-1*, ***c*** = Col-0, ***d*** = *tpt-2*, ***e*** =*tpt-2/xpt-1*, ***f*** = amiRNA:*XPT tpt-2* #3, and ***g*** = amiRNA:*XPT tpt-2* #4. DAS = days after sowing. The significance levels of P < 0.05 or P < 0.01 are indicated by light or dark blue colors.

7 comparisons

Growth parameter	*b vs a*	*c vs a*	*d vs a*	*e vs a*	*f vs a*	*g vs a*	*c vs b*	*d vs b*	*e vs b*	*f vs b*	*g vs b*	*d vs c*	*e vs c*	*f vs c*	*g vs c*	*e vs d*	*f vs d*	*g vs d*	*f vs e*	*g vs e*	*g vs f*
**Rosette area**																					
9 DAS									light	light											
13 DAS									dark	dark	dark		light	light		light	light				
17 DAS		light		dark	light	light		light	dark	dark	dark	dark	dark	dark	dark	dark					
21 DAS			light	dark	dark	dark		light	dark	dark	dark	dark	dark	dark	dark	dark	dark	dark			
**Root length**																					
3 DAS							dark	dark	dark	light	dark										
6 DAS			dark	dark	dark	dark	dark	dark	dark	dark	dark		dark	light	dark	light					
9 DAS		light	dark	dark	dark	dark	dark	dark	dark	dark	dark		dark	dark	dark	dark		dark			
12 DAS		dark	dark	dark	dark	dark	dark	dark	dark	dark	dark		dark	dark	dark	dark		dark			
**Generative growth**																					
Inflorescence height	dark			dark			dark	dark	dark	dark	dark		dark			dark			dark	dark	
Number of sliques	light	dark	dark	dark	dark	dark	dark	dark	dark	dark	dark		dark			dark			dark	light	
Seed weight per plant		light	dark	dark	dark	dark			dark	light	dark		dark		dark	dark		light	dark		
Seed weight per 100 seeds	dark						dark	dark	dark	dark	dark										
Seed number per plant	dark	dark	dark	dark	dark	dark			dark				dark		dark	dark		light	dark		
Seed area	dark		dark	light		dark	dark	dark	dark	dark	dark	dark	dark		dark	dark	dark	dark	dark	dark	dark

Doc S2 Table 2. Statistical analysis (ANOVA/Tukey-Kramer) of Chl *a* fluorescence parameters of wild-type and mutant plants impaired in XPT and TPT as well as starch biosynthesis. The growth conditions and experimental setup is described in Supplementary Table 2. The biotypes are denoted, ***a*** = Ws-2, ***b*** = Col-0, ***c*** = *tpt-2*, ***d*** = *tpt-2/xpt-1*, ***e*** = amiRNA:*XPT tpt-2* #3, ***f*** = amiRNA:*XPT tpt-2* #4, ***g*** = *adg1-1/tpt-2*, ***h*** = *xpt-1*, ***i*** = *tpt-1, and **j*** = *tpt-1/xpt-1.* The significance levels of $P < 0.05$ or $P < 0.01$ are indicated by light or dark blue colors.

7 Comparisons

PS Parameters	*b vs a*	*c vs a*	*d vs a*	*e vs a*	*f vs a*	*g vs a*	*c vs b*	*d vs b*	*e vs b*	*f vs b*	*g vs b*	*d vs c*	*e vs c*	*f vs c*	*g vs c*	*e vs d*	*f vs d*	*g vs d*	*f vs e*	*g vs e*	*f vs g*
**Long-day HL**																					
F_v/F_m			dark blue		light blue	dark blue		dark blue		light blue	dark blue	dark blue			dark blue	dark blue	dark blue	dark blue		dark blue	dark blue
ΦPSII			dark blue		dark blue	dark blue		dark blue		dark blue	dark blue	dark blue		dark blue	dark blue	dark blue			dark blue	dark blue	dark blue
Relative $ETR_{(700)}$			dark blue		dark blue	dark blue		dark blue		dark blue	dark blue	dark blue		dark blue	dark blue	dark blue			dark blue	dark blue	

4 Comparisons

PS Parameters	*h vs a*	*i vs a*	*j vs a*	*i vs h*	*j vs h*	*i vs j*
**Long-day HL**						
F_v/F_m			dark blue	light blue	dark blue	dark blue
ΦPSII		light blue	dark blue	light blue	dark blue	dark blue
Relative $ETR_{(700)}$		dark blue	dark blue	dark blue	dark blue	dark blue

Doc S2 Table 3. Statistical analysis (ANOVA/Tukey-Kramer) of rosette leaf parameters, i.e. photosynthetic pigments and protein contents as well as specific fresh weights. Plants were grown under HL- (PFD = 300 µmol·m^{-2}·s^{-1}; Tabel 3, main article) or LL-conditions (PFD = 30 µmol·m^{-2}·s^{-1}; Supplementary Table 3). The biotypes are denoted, ***a*** = Ws-2, ***b*** = Col-0, ***c*** = *tpt-2*, ***d*** = *tpt-2/xpt-1*, ***e*** = amiRNA:*XPT tpt-2* #3, ***f*** = amiRNA:*XPT tpt-2* #4, ***g*** = *adg1-1/tpt-2,* ***h*** = *xpt-1,* ***i*** = *tpt-1, and* ***j*** = *tpt-1/xpt-1.* The significance levels of P < 0.05 or P < 0.01 are indicated by light or dark blue colors.

7 Comparisons

Parameters	*b vs a*	*c vs a*	*d vs a*	*e vs a*	*f vs a*	*g vs a*	*c vs b*	*d vs b*	*e vs b*	*f vs b*	*g vs b*	*d vs c*	*e vs c*	*f vs c*	*g vs c*	*e vs d*	*f vs d*	*g vs d*	*f vs e*	*g vs e*	*g vs f*
**A High light**																					
Chl																					
Carotenoids																					
Chl *a/b*-ratio																					
Protein																					
Spec. fw																					
**B Low light**																					
Chl																					
Carotenoids																					
Chl *a/b*-ratio																					
Protein																					
Spec. fw																					

4 Comparisons

Parameters	*h vs a*	*i vs a*	*j vs a*	*i vs h*	*j vs h*	*j vs i*
**A High light**						
Chl						
Carotenoids						
Chl *a/b*-ratio						
Protein						
Spec. fw						
**B Low light**						
Chl						
Carotenoids						
Chl *a/b*-ratio						
Protein						
Spec. fw						

Doc S2 Table 4. Statistical analysis (ANOVA/Tukey-Kramer) of carbohydrate contents in rosette leaves of wild-type and mutant plants. The plants were grown in soil under HL-conditions (PFD = 300 µmol·m^{-2}·s^{-1}) in the long-day (Figure 4, main article). The biotypes are denoted, ***a*** = Ws-2, ***b*** = *xpt-1*, ***c*** = *tpt-1*, ***d*** = *tpt-1/xpt-1*, ***e*** = Col-0, ***f*** = *tpt-2*, ***g*** = *tpt-2/xpt-1*, and ***h*** = *adg1-1/tpt-2*. The significance levels of P < 0.05 or P < 0.01 are indicated by light or dark blue colors.

8 Comparisons

Time course	*b vs a*	*c vs a*	*d vs a*	*e vs a*	*f vs a*	*g vs a*	*h vs a*	*c vs b*	*d vs b*	*e vs b*	*f vs b*	*g vs b*	*h vs b*	*d vs c*	*e vs c*	*f vs c*	*g vs c*	*h vs c*	*e vs d*	*f vs d*	*g vs d*	*h vs d*	*f vs e*	*g vs e*	*h vs e*	*g vs f*	*h vs f*	*h vs g*
**A Starch**																												
1 h																												
4 h																												
8 h																												
12 h																												
15 h																												
17 h																												
20 h																												
23 h																												
**B Σ Sugars**																												
1 h																												
4 h																												
8 h																												
12 h																												
15 h																												
17 h																												
20 h																												
23 h																												

Doc S2 Table4 (continued)

Time course	*b vs a*	*c vs a*	*d vs a*	*e vs a*	*f vs a*	*g vs a*	*h vs a*	*c vs b*	*d vs b*	*e vs b*	*f vs b*	*g vs b*	*h vs b*	*d vs c*	*e vs c*	*f vs c*	*g vs c*	*h vs c*	*e vs d*	*f vs d*	*g vs d*	*h vs d*	*f vs e*	*g vs e*	*h vs e*	*g vs f*	*h vs f*	*h vs g*
**C Glc**																												
1 h																												
4 h																												
8 h																												
12 h																												
15 h																												
17 h																												
20 h																												
23 h																												
**D Fru**																												
1 h																												
4 h																												
8 h																												
12 h																												
15 h																												
17 h																												
20 h																												
23 h																												

a = Ws-2, ***b*** = *xpt-1*, ***c*** = *tpt-1*, ***d*** = *tpt-1/xpt-1*, ***e*** = Col-0, ***f*** = *tpt-2*, ***g*** = *tpt-2/xpt-1*, and ***h*** = *adg1-1/tpt-2*

Doc S2 Table4 (continued)

Time course	*b vs a*	*c vs a*	*d vs a*	*e vs a*	*f vs a*	*g vs a*	*h vs a*	*c vs b*	*d vs b*	*e vs b*	*f vs b*	*g vs b*	*h vs b*	*d vs c*	*e vs c*	*f vs c*	*g vs c*	*h vs c*	*e vs d*	*f vs d*	*g vs d*	*h vs d*	*f vs e*	*g vs e*	*h vs e*	*g vs f*	*h vs f*	*h vs g*
**E Suc**																												
1 h																												
4 h																												
8 h																												
12 h																												
15 h																												
17 h																												
20 h																												
23 h																												

a = Ws-2, ***b*** = *xpt-1*, ***c*** = *tpt-1*, ***d*** = *tpt-1/xpt-1*, ***e*** = Col-0, ***f*** = *tpt-2*, ***g*** = *tpt-2/xpt-1*, and ***h*** = *adg1-1/tpt-2*

Doc S2 Table 5. Statistical analysis (ANOVA/Tukey-Kramer) of metabolite contents in leaves of wild-type and mutant plants. The plants were grown in soil under HL-conditions (PFD = 300 µmol·m^{-2}·s^{-1}) in the long-day (see Figure 5, and Supplementary Table 5). Samples were taken at the middle of the dark period (**A**), or the beginning (**B**), and middle of the light period (**C**). The biotypes are denoted, ***a*** = Ws-2, ***b*** = *xpt-1,* ***c*** = *tpt-1/xpt-1*, ***d*** = Col-0, ***e*** = *tpt-2*, and ***f*** = *tpt-2/xpt-1*. The significance levels of P < 0.05 or P < 0.01 are indicated by light or dark blue colors.

6 Comparisons

A Middle of the dark period (5 h in the dark)

**Metabolites**	*b vs a*	*c vs a*	*d vs a*	*e vs a*	*f vs a*	*c vs b*	*d vs b*	*e vs b*	*f vs b*	*d vs c*	*e vs c*	*f vs c*	*e vs d*	*f vs d*	*e vs f*
3-PGA				light				dark			dark		dark		light
DHAP															
S-7-P		dark				dark				dark	dark	dark			
Ri5P															
Xu5P+Ru5P		dark			dark	dark			dark	dark	dark			dark	dark
Fru6P															
Glc6P															
Glc1P															
UDPG															
Glycerol-3-P															
ATP															
AMP															
Asn					light										
Phe					dark				dark			dark		dark	dark
Tyr					dark				dark			dark		dark	dark
Trp					light				light					dark	light

B Beginning of the light period (1 h in the light)

	b vs a	*c vs a*	*d vs a*	*e vs a*	*f vs a*	*c vs b*	*d vs b*	*e vs b*	*f vs b*	*d vs c*	*e vs c*	*f vs c*	*e vs d*	*f vs d*	*e vs f*
3-PGA															
DHAP															
S-7-P															
Ri5P															
Xu5P+Ru5P		dark				dark				dark				light	
Fru6P															
Glc6P															
Glc1P															
UDPG															
Glycerol-3-P															
ATP															
AMP															
Asn															
Phe		light													
Tyr					dark				dark			dark		dark	dark
Trp					dark				dark			dark		dark	dark

Doc S2 Table 5 (continued)

C Middle of the light period (8 h in the light)

	b vs a	***c vs a***	***d vs a***	***e vs a***	***f vs a***	***c vs b***	***d vs b***	***e vs b***	***f vs b***	***d vs c***	***e vs c***	***f vs c***	***e vs d***	***f vs d***	***e vs f***
3-PGA															
DHAP															
S-7-P															
Ri5P															
Xu5P+Ru5P															
Fru6P															
Glc6P															
Glc1P															
UDPG															
Glycerol-3-P															
ATP															
AMP															
Asn															
Phe															
Tyr															
Trp															

a = Ws-2, ***b*** = *xpt-1*, ***c*** = *tpt-1/xpt-1*, ***d*** = Col-0, ***e*** = *tpt-2*, and ***f*** = *tpt-2/xpt-1*.

Doc S2 Table 6 Statistical analysis (ANOVA/Tukey-Kramer) of metabolite contents in leaves of wild-type and mutant plants. The plants were grown in soil under HL-conditions (PFD = 300 µmol·m^{-2}·s^{-1}) in the long day (see Figure 5, and Supplementary Table 5). Samples were taken at the middle of the dark period (**A**), or the beginning (**B**) and middle of the light period (**C**). The biotypes are denoted, ***a*** = Ws-2, ***b*** = *xpt-1, **c*** = *tpt-1/xpt-1*, ***d*** = Col-0, ***e*** = *tpt-2*, and ***f*** = *tpt-2/xpt-1*. The significance levels of P < 0.05 or P < 0.01 are indicated by light or dark blue colors.

A Middle of the dark period (5 h in the dark)

6 Comparisons

**Metabolites**	*b vs a*	*c vs a*	*d vs a*	*e vs a*	*f vs a*	*c vs b*	*d vs b*	*e vs b*	*f vs b*	*d vs c*	*e vs c*	*f vs c*	*e vs d*	*f vs d*	*e vs f*
Glu															
Asp					*										
Ser															
Gly						**	*	**	**						
Thr															
Pro															
Phe															
Tyr									*			*		*	
Val															
Leu															
Ile															
Lys															
Met															
β-Ala					**			*	**			**		**	**
Glycerol															
Mannitol									*					*	
Myoinositol				*											
Sorbitol															
Putrescine															
(Iso) Citrate															
2-Oxoglutarate					**					*				**	
Succinate					**				**			**		**	**
Fumarate									*						
Malate															
Glycolate															
Lactate															
Glycerate		**			**	*			**	**				**	**
Gluconate					**				**			**		**	**
Maleate															
Malonate									*					*	
Shikimate															*
Quinate															
Glc														**	
Fru		**			**	*			**	**				**	**
Suc															
Maltose		**		**	**	**		**	**	**	**	**	**	**	
Mannose							*							**	
Xylose															

Doc S2 Table 6 (continued)

B Beginning of the light period (1 h in the light)

**Metabolite**	***b* vs *a***	***c* vs *a***	***d* vs *a***	***e* vs *a***	***f* vs *a***	***c* vs *b***	***d* vs *b***	***e* vs *b***	***f* vs *b***	***d* vs *c***	***e* vs *c***	***f* vs *c***	***e* vs *d***	***f* vs *d***	***e* vs *f***
Glu															
Asp															
Ser															
Gly															
Thr															
Pro															
Phe															
Tyr															
Val															
Leu															
Ile															
Lys															
Met															
β-Ala															
Glycerol															
Mannitol															
Myoinositol															
Sorbitol															
Putrescine															
(Iso) Citrate															
2-Oxoglutarate															
Succinate															
Fumarate															
Malate															
Glycolate															
Lactate															
Glycerate															
Gluconate															
Maleate															
Malonate															
Shikimate															
Quinate															
Glc															
Fru															
Suc															
Maltose															
Mannose															
Xylose															

a = Ws-2, ***b*** = *xpt-1,* ***c*** = *tpt-1/xpt-1*, ***d*** = Col-0, ***e*** = *tpt-2*, and ***f*** = *tpt-2/xpt-1*.

Doc S2 Table 6 (continued)

C Middle of the light period (8 h in the light)

**Metabolites**	*b vs a*	*c vs a*	*d vs a*	*e vs a*	*f vs a*	*c vs b*	*d vs b*	*e vs b*	*f vs b*	*d vs c*	*e vs c*	*f vs c*	*e vs d*	*f vs d*	*e vs f*
Glu															
Asp															
Ser															
Gly															
Thr															
Pro															
Phe															
Tyr															
Val															
Leu															
Ile															
Lys															
Met															
β-Ala															
Glycerol															
Mannitol															
Myoinositol															
Sorbitol															
Putrescine															
(Iso) Citrate															
2-Oxoglutarate															
Succinate															
Fumarate															
Malate															
Glycolate															
Lactate															
Glycerate															
Gluconate															
Maleate															
Malonate															
Shikimate															
Quinate															
Glc															
Fru															
Suc															
Maltose															
Mannose															
Xylose															

a = Ws-2, ***b*** = *xpt-1*, ***c*** = *tpt-1/xpt-1*, ***d*** = Col-0, ***e*** = *tpt-2*, and ***f*** = *tpt-2/xpt-1*.

Doc S2 Table 7. Statistical analysis (ANOVA/Tukey-Kramer) of soluble amino acid contents in leaves of wild-type and mutant plants. The plants were grown in soil under HL-conditions (PFD = 300 µmol·m^{-2}·s^{-1}) in the long-day (Figure 6C, main article). Samples were taken either 4 h in the dark (**A**, **C**) or 8 h in the light (**B**, **D**). The data represents the mean ± SE of n = 4 to 5 independent replicates. The biotypes are denoted, ***a*** = Ws-2, ***b*** = Col-0, ***c*** = *tpt-2*, ***d*** = *tpt-2/xpt-1*, ***e*** = amiRNA:*XPT tpt-2* #3, ***f*** = amiRNA:*XPT tpt-2* #4, ***g*** = *adg1-1/tpt-2*, ***h*** = *xpt-1*, ***i*** = *tpt-1*, and ***j*** = *tpt-1/xpt-1*. The significance levels of P < 0.05 or P < 0.01 are indicated by light or dark blue colors.

A 4 h dark (absolute)

7 Comparisons

	b vs a	*c vs a*	*d vs a*	*e vs a*	*f vs a*	*g vs a*	*c vs b*	*d vs b*	*e vs b*	*f vs b*	*g vs b*	*d vs c*	*e vs c*	*f vs c*	*g vs c*	*e vs d*	*f vs d*	*g vs d*	*f vs e*	*g vs e*	*f vs g*
**Amino acids**																					
Glu								dark			light	light				light	dark				dark
Gln		dark	dark	dark	dark	dark	dark	dark	dark	dark	dark							dark			
Asp					dark	dark					dark	light			dark		dark	dark		dark	dark
Asn						dark					dark				dark			dark		dark	dark
Ala	light		dark					dark				dark				dark	dark	dark			
Ser	light						dark	light	dark	dark	dark						light				
Gly	light	dark	light	dark	light	light															
Thr			dark			dark		dark			dark	dark			dark	dark	dark			dark	dark
His	dark	dark		dark	dark										dark					light	dark
Arg	dark					light	dark	dark	dark	dark					light					dark	dark
Phe	dark	light	light		dark			dark			dark	dark			dark	dark	dark			dark	dark
Tyr						dark		dark			dark	dark			dark	light	dark	dark		dark	dark
Trp			light			dark		dark			dark	dark			dark	light	dark			dark	dark
Val	dark	dark		light	dark	dark		dark			dark	dark			dark	dark	dark	dark		dark	dark
Leu		light	dark	dark	light	dark		dark	light		dark	dark			dark	dark	dark			dark	dark
Ile						dark		light			dark	dark			dark	dark	dark	dark		dark	dark
Lys						dark					dark				dark			light		dark	dark
Σ		dark		dark	dark	dark	dark		dark	dark		light				dark	dark				

4 comparisons

	h vs a	*i vs a*	*j vs a*	*i vs h*	*j vs h*	*i vs j*
**Amino acids**						
Glu			dark		dark	dark
Gln		dark	dark	dark	dark	
Asp			dark		dark	dark
Asn		dark	dark	dark	dark	
Ala		light				
Ser	dark			dark	dark	
Gly				dark	dark	
Thr			dark	light		dark
His		light	light			
Arg		dark	light	dark		
Phe						
Tyr			dark		dark	
Trp			light		light	
Val						
Leu					light	
Ile			light		light	
Lys			light		light	
Σ		dark	light	dark	dark	

Doc S2 Table 7. (continued)

B 8 h light (absolute)

	b vs a	*c vs a*	*d vs a*	*e vs a*	*f vs a*	*g vs a*	*c vs b*	*d vs b*	*e vs b*	*f vs b*	*g vs b*	*d vs c*	*e vs c*	*f vs c*	*g vs c*	*e vs d*	*f vs d*	*g vs d*	*f vs e*	*g vs e*	*f vs g*
**Amino acids**																					
Glu																					
Gln																					
Asp																					
Asn																					
Ala																					
Ser																					
Gly																					
Thr																					
His																					
Arg																					
Phe																					
Tyr																					
Trp																					
Val																					
Leu																					
Ile																					
Lys																					
$\sum$																					

	h vs a	*i vs a*	*j vs a*	*i vs h*	*j vs h*	*i vs j*
**Amino acids**						
Glu						
Gln						
Asp						
Asn						
Ala						
Ser						
Gly						
Thr						
His						
Arg						
Phe						
Tyr						
Trp						
Val						
Leu						
Ile						
Lys						
$\sum$						

a = Ws-2, ***b*** = Col-0, ***c*** = *tpt-2*, ***d*** = *tpt-2/xpt-1*, ***e*** = amiRNA:*XPT tpt-2* #3, ***f*** = amiRNA:*XPT tpt-2* #4, ***g*** = *adg1-1/tpt-2*, ***h*** = *xpt-1*, ***i*** = *tpt-1*, and ***j*** = *tpt-1/xpt-1*.

Doc S2 Table 7. (continued)

C 4 h dark (relative)

	b vs a	*c vs a*	*d vs a*	*e vs a*	*f vs a*	*g vs a*	*c vs b*	*d vs b*	*e vs b*	*f vs b*	*g vs b*	*d vs c*	*e vs c*	*f vs c*	*g vs c*	*e vs d*	*f vs d*	*g vs d*	*f vs e*	*g vs e*	*f vs g*
**Amino acids**																					
Glu																					
Gln																					
Asp																					
Asn																					
Ala																					
Ser																					
Gly																					
Thr																					
His																					
Arg																					
Phe																					
Tyr																					
Trp																					
Val																					
Leu																					
Ile																					
Lys																					

	h vs a	*i vs a*	*j vs a*	*i vs h*	*j vs h*	*i vs j*
**Amino acids**						
Glu						
Gln						
Asp						
Asn						
Ala						
Ser						
Gly						
Thr						
His						
Arg						
Phe						
Tyr						
Trp						
Val						
Leu						
Ile						
Lys						

a = Ws-2, ***b*** = Col-0, ***c*** = *tpt-2*, ***d*** = *tpt-2/xpt-1*, ***e*** = amiRNA:*XPT tpt-2* #3, ***f*** = amiRNA:*XPT tpt-2* #4, ***g*** = *adg1-1/tpt-2*, ***h*** = *xpt-1*, ***i*** = *tpt-1*, and ***j*** = *tpt-1/xpt-1*.

Doc S2 Table 7. (continued)

D 8 h light (relative)

	b *vs* a	c *vs* a	d *vs* a	e *vs* a	f *vs* a	g *vs* a	c *vs* b	d *vs* b	e *vs* b	f *vs* b	g *vs* b	d *vs* c	e *vs* c	f *vs* c	g *vs* c	e *vs* d	f *vs* d	g *vs* d	f *vs* e	g *vs* e	f *vs* g	h *vs* a	i *vs* a	j *vs* a	i *vs* h	j *vs* h	i *vs* j
**Amino acids**																											
Glu																											
Gln																											
Asp																											
Asn																											
Ala																											
Ser																											
Gly																											
Thr																											
His																											
Arg																											
Phe																											
Tyr																											
Trp																											
Val																											
Leu																											
Ile																											
Lys																											

a = Ws-2, ***b*** = Col-0, ***c*** = *tpt-2*, ***d*** = *tpt-2/xpt-1*, ***e*** = amiRNA:*XPT tpt-2* #3, ***f*** = amiRNA:*XPT tpt-2* #4, ***g*** = *adg1-1/tpt-2*, ***h*** = *xpt-1*, ***i*** = *tpt-1*, and ***j*** = *tpt-1/xpt-1*.

Doc S2 Table 8. Statistical analysis (ANOVA/Tukey-Kramer) of elemental analyses (N, C) in rosette leaves as well as fw/dw-ratios of wild-type and mutant plants. The plants were grown in soil under HL-conditions (PFD = 300 µmol·m^{-2}·s^{-1}) in the long-day (Table 3, main article). The biotypes are denoted, ***a*** = Ws-2, ***b*** = *xpt-1,* ***c*** = *tpt-1*, ***d*** = *tpt-1/xpt-1*, ***e*** = Col-0, ***f*** = *tpt-2*, ***g*** = *tpt-2/xpt-1*, and ***h*** = *adg1-1/tpt-2.* The significance levels of $P < 0.05$ or $P < 0.01$ are indicated by light or dark blue colors.

8 Comparisons

**Element contents and ratios**	***b vs a***	***c vs a***	***d vs a***	***e vs a***	***f vs a***	***g vs a***	***h vs a***	***c vs b***	***d vs b***	***e vs b***	***f vs b***	***g vs b***	***h vs b***	***d vs c***	***e vs c***	***f vs c***	***g vs c***	***h vs c***	***e vs d***	***f vs d***	***g vs d***	***h vs d***	***f vs e***	***g vs e***	***h vs e***	***g vs f***	***h vs f***	***h vs g***
fw/dw-ratio																												
N (%)																												
C (%)																												
C/N-ratio																												

Doc S2 Table 9. Statistical analysis (ANOVA/Tukey-Kramer) of feeding Suc, Gln or both on rosette growth of wild-type and mutant plants. Plants were grown under HL-conditions (i.e. a PFD of 300 µmol·m^{-2}·s^{-1}) in the long-day on 1/2 Ms agar plates in the absence or presence of 50 mM Suc, 2 mM Gln, or both. under HL. (Figure 7, main article; Supplementary Figures 2 and 3). The biotypes are denoted, ***a*** = Ws-2, ***b*** = *tpt-1/xpt-1,* ***c*** = Col-0, ***d*** = *tpt-2/xpt-1*, ***e*** = *gpt2-1/tpt-2/xpt-1*, and ***f*** = *adg1-1/tpt-2.* DAS = days after sowing. The significance levels of P < 0.05 or P < 0.01 are indicated by light or dark blue colors.

6 Comparisons

Time course rosette growth	*b vs a*	*c vs a*	*d vs a*	*e vs a*	*f vs a*	*c vs b*	*d vs b*	*e vs b*	*f vs b*	*d vs c*	*e vs c*	*f vs c*	*e vs d*	*f vs d*	*f vs e*
**½ MS**															
11 DAS															
15 DAS															
19 DAS															
22 DAS															
**+Gln**															
11 DAS															
15 DAS															
19 DAS															
22 DAS															

6 Comparisons

Time course rosette growth	*b vs a*	*c vs a*	*d vs a*	*e vs a*	*f vs a*	*c vs b*	*d vs b*	*e vs b*	*f vs b*	*d vs c*	*e vs c*	*f vs c*	*e vs d*	*f vs d*	*f vs e*
**+Suc**															
11 DAS															
15 DAS															
19 DAS															
22 DAS															
**+Suc + Gln**															
11 DAS															
15 DAS															
19 DAS															
22 DAS															

Doc S2 Table 10. Statistical analysis (ANOVA/Tukey-Kramer) of feeding Suc, Gln or both on rosette growth of wild-type and mutant plants. Plants were grown under HL-conditions (i.e. a PFD of 300 µmol·m^{-2}·s^{-1}) in the long-day on 1/2 Ms agar plates in the absence or presence of 50 mM Suc, 2 mM Gln, or both. under HL. (Figure 7, main article; Supplementary Figures 2 and 3). The conditions are denoted, ***a*** = 1/2 MS, ***b*** = 50 mM Suc, ***c*** = 2 mM Gln, and ***d*** = 50 mM Suc and 2 mM Gln. DAS = days after sowing. The significance levels of P < 0.05 or P < 0.01 are indicated by light or dark blue colors.

4 Comparisons

Time of growth	*b vs a*	*c vs a*	*d vs a*	*c vs b*	*d vs b*	*d vs c*
**Ws-2**						
11 DAS			[dark blue]			
15 DAS			[light blue]			[light blue]
19 DAS						
22 DAS						
tpt-2/xpt-1						
11 DAS	[dark blue]		[dark blue]	[dark blue]		[dark blue]
15 DAS	[dark blue]		[dark blue]	[dark blue]		[dark blue]
19 DAS	[dark blue]		[dark blue]	[dark blue]		[dark blue]
22 DAS	[dark blue]		[dark blue]	[dark blue]		[dark blue]

4 Comparisons

Time of growth	*b vs a*	*c vs a*	*d vs a*	*c vs b*	*d vs b*	*d vs c*
tpt-1/xpt-1						
11 DAS	[light blue]		[dark blue]			[dark blue]
15 DAS	[light blue]		[dark blue]		[light blue]	[dark blue]
19 DAS			[dark blue]			[light blue]
22 DAS			[dark blue]			[light blue]
gpt2-1/tpt-2/xpt-1						
11 DAS	[dark blue]		[dark blue]	[dark blue]		[dark blue]
15 DAS	[dark blue]		[dark blue]	[dark blue]		[dark blue]
19 DAS	[dark blue]		[dark blue]	[dark blue]		[dark blue]
22 DAS	[dark blue]		[dark blue]	[dark blue]		[dark blue]

4 Comparisons

Time of growth	*b vs a*	*c vs a*	*d vs a*	*c vs b*	*d vs b*	*d vs c*
**Col-0**						
11 DAS						
15 DAS			[light blue]			[light blue]
19 DAS						
22 DAS						
adg1-1/tpt-2						
11 DAS	[dark blue]		[dark blue]	[dark blue]		[dark blue]
15 DAS	[dark blue]		[dark blue]	[dark blue]		[dark blue]
19 DAS	[dark blue]		[dark blue]	[dark blue]		[dark blue]
22 DAS	[dark blue]		[dark blue]	[dark blue]	[light blue]	[dark blue]

Doc S2 Table 11. Statistical analysis (ANOVA/Tukey-Kramer) of feeding Suc, Gln or both on modulated Chl *a* fluorescence parameters in wild-type and mutant plants. Plants were grown under HL-conditions (i.e. a PFD of 300 µmol·m^{-2}·s^{-1}) in the long-day on 1/2 Ms agar plates in the absence or presence of 50 mM Suc, 2 mM Gln, or both. (Figure 7, main article; Supplementary Figure 4). The biotypes are denoted, ***a*** = Ws-2, ***b*** = *tpt-1/xpt-1,* ***c*** = Col-0, ***d*** = *tpt-2/xpt-1*, ***e*** = *gpt2-1/tpt-2/xpt-1*, and ***f*** = *adg1-1/tpt-2*. The significance levels of P < 0.05 or P < 0.01 are indicated by light or dark blue colors.

6 Comparisons

PS-parameters	*b vs a*	*c vs a*	*d vs a*	*e vs a*	*f vs a*	*c vs b*	*d vs b*	*e vs b*	*f vs b*	*d vs c*	*e vs c*	*f vs c*	*e vs d*	*f vs d*	*f vs e*
**½ MS**															
F_v/F_m															
ΦPSII															
**+Gln**															
F_v/F_m															
ΦPSII															

6 Comparisons

PS-parameters	*b vs a*	*c vs a*	*d vs a*	*e vs a*	*f vs a*	*c vs b*	*d vs b*	*e vs b*	*f vs b*	*d vs c*	*e vs c*	*f vs c*	*e vs d*	*f vs d*	*f vs e*
**+Suc**															
F_v/F_m															
ΦPSII															
**+Suc + Gln**															
F_v/F_m															
ΦPSII															

Doc S2 Table 12. Statistical analysis (ANOVA/Tukey-Kramer) of feeding Suc, Gln or both on modulated Chl *a* fluorescence parameters in wild-type and mutant plants. Plants were grown under HL-conditions (i.e. a PFD of 300 µmol·m^{-2}·s^{-1}) in the long-day on 1/2 Ms agar plates in the absence or presence of 50 mM Suc, 2 mM Gln, or both. (Figure 7, main article; Supplementary Figure 4). The conditions are denoted, ***a*** = 1/2 MS, ***b*** = 50 mM Suc, ***c*** = 2 mM Gln, and ***d*** = 50 mM Suc and 2 mM Gln. The significance levels of P < 0.05 or P < 0.01 are indicated by light or dark blue colors.

4 Comparisons

PS parameters	*b vs a*	*c vs a*	*d vs a*	*c vs b*	*d vs b*	*d vs c*
**Ws-2**						
F_v/F_m	dark blue	dark blue	dark blue	dark blue		dark blue
ΦPSII	light blue	dark blue	dark blue	dark blue	dark blue	
tpt-2/xpt-1						
F_v/F_m	dark blue	dark blue	dark blue	dark blue		dark blue
ΦPSII	dark blue		dark blue	dark blue		

4 Comparisons

PS parameters	*b vs a*	*c vs a*	*d vs a*	*c vs b*	*d vs b*	*d vs c*
tpt-1/xpt-1						
F_v/F_m	dark blue		dark blue			
ΦPSII	light blue			light blue		
gpt2-1/tpt-2/xpt-1						
F_v/F_m	dark blue					
ΦPSII					light blue	

4 Comparisons

PS parameters	*b vs a*	*c vs a*	*d vs a*	*c vs b*	*d vs b*	*d vs c*
**Col-0**						
F_v/F_m	light blue		dark blue		light blue	dark blue
ΦPSII		dark blue	dark blue	dark blue	dark blue	
adg1-1/tpt-2						
F_v/F_m	dark blue	dark blue	dark blue	dark blue		dark blue
ΦPSII	dark blue		dark blue	dark blue		dark blue

Doc S2 Table 13. Statistical analysis (ANOVA/Tukey-Kramer) of spectroscopic analysis of thylakoid membranes isolated from wild-type and mutants plants. Plants were grown in soil under HL-conditions (PFD = 300 µmol·m^{-2}·s^{-1}) in the long-day. Functional components of PSII, PSI and the Cyt b_6/f complex were determined by spectroscopic methods using thylakoids isolated from the plants and were either referred to a leaf area- (Figure 8B, main article) or Chl basis (Supplementary Figure 5). The biotypes are denoted, ***a*** = Col-0, ***b*** = *tpt-2,* ***c*** = *tpt-2/xpt-1*, ***d*** = amiRNA:*XPT tpt-2* #4, ***e*** = Ws-2, ***f*** = *xpt-1*, ***g*** = *tpt-1*, and ***h*** = *tpt-1/xpt-1.* The significance levels of P < 0.05 or P < 0.01 are indicated by light or dark blue colors.

8 Comparisons

**PS complexes**	***b vs a***	***c vs a***	***d vs a***	***e vs a***	***f vs a***	***g vs a***	***h vs a***	***c vs b***	***d vs b***	***e vs b***	***f vs b***	***g vs b***	***h vs b***	***d vs c***	***e vs c***	***f vs c***	***g vs c***	***h vs c***	***e vs d***	***f vs d***	***g vs d***	***h vs d***	***f vs e***	***g vs e***	***h vs e***	***g vs f***	***h vs f***	***h vs g***
**Leaf area basis**																												
PSII																												
Cyt b_6/f																												
PSI																												
**Chl basis**																												
Chl *a/b*-ratio																												
PSII																												
Cyt b_6/f																												
PSI																												
